# A biallelic multiple nucleotide length polymorphism explains functional causality at 5p15.33 prostate cancer risk locus

**DOI:** 10.1038/s41467-023-40616-z

**Published:** 2023-08-23

**Authors:** Sandor Spisak, Viktoria Tisza, Pier Vitale Nuzzo, Ji-Heui Seo, Balint Pataki, Dezso Ribli, Zsofia Sztupinszki, Connor Bell, Mersedeh Rohanizadegan, David R. Stillman, Sarah Abou Alaiwi, Alan H. Bartels, Marton Papp, Anamay Shetty, Forough Abbasi, Xianzhi Lin, Kate Lawrenson, Simon A. Gayther, Mark Pomerantz, Sylvan Baca, Norbert Solymosi, Istvan Csabai, Zoltan Szallasi, Alexander Gusev, Matthew L. Freedman

**Affiliations:** 1 Department of Medical Oncology, Dana-Farber Cancer Institute, Boston, MA 02215 USA; 2 Center for Functional Cancer Epigenetics, Dana-Farber Cancer Institute, Boston, MA 02215 USA; 3 Computational Health Informatics Program (CHIP) Boston Children’s Hospital Harvard Medical School, Boston, MA 02215 USA; 4 Institute of Enzymology, Research Centre for Natural Sciences, Budapest, 1117 Hungary; 5 Department of Internal Medicine, School of Medicine, University of Genoa, Genoa, Lgo R. Benzi 10, 16132 Italy; 6 Department of Physics of Complex Systems, ELTE Eötvös Loránd University, Pázmány P. s. 1A, Budapest, 1117 Hungary; 7 Centre for Bioinformatics, University of Veterinary Medicine, Istvan str. 2, Budapest, 1078 Hungary; 8 Division of Genetics, Brigham & Women’s Hospital, Boston, MA USA; 9 Women’s Cancer Program, Samuel Oschin Comprehensive Cancer Institute, Cedars-Sinai Medical Center, Los Angeles, CA 90048 USA; 10 Division of Gynecologic Oncology, Department of Obstetrics and Gynecology, Cedars-Sinai Medical Center, Los Angeles, CA 90048 USA; 11 Center for Bioinformatics and Functional Genomics, Department of Biomedical Science, Cedars-Sinai Medical Center, Los Angeles, CA 90048 USA; 12 The Eli and Edythe L. Broad Institute, Cambridge, MA 02142 USA; 13 Department of Bioinformatics, Forensic and Insurance Medicine Semmelweis University, Budapest, Hungary; 14 Danish Cancer Society Research Center, Strandboulevarden 49, 2100 Copenhagen, Denmark; 15 National Korányi Institute of Pulmonology, Budapest, 1112 Hungary

**Keywords:** Cancer epigenetics, Cancer genomics, Prostate cancer

## Abstract

To date, single-nucleotide polymorphisms (SNPs) have been the most intensively investigated class of polymorphisms in genome wide associations studies (GWAS), however, other classes such as insertion-deletion or multiple nucleotide length polymorphism (MNLPs) may also confer disease risk. Multiple reports have shown that the 5p15.33 prostate cancer risk region is a particularly strong expression quantitative trait locus (eQTL) for Iroquois Homeobox 4 (*IRX4)* transcripts. Here, we demonstrate using epigenome and genome editing that a biallelic (21 and 47 base pairs (bp)) MNLP is the causal variant regulating *IRX4* transcript levels. In LNCaP prostate cancer cells (homozygous for the 21 bp short allele), a single copy knock-in of the 47 bp long allele potently alters the chromatin state, enabling de novo functional binding of the androgen receptor (*AR*) associated with increased chromatin accessibility, Histone 3 lysine 27 acetylation (H3K27ac), and ~3-fold upregulation of *IRX4* expression. We further show that an MNLP is amongst the strongest candidate susceptibility variants at two additional prostate cancer risk loci. We estimated that at least 5% of prostate cancer risk loci could be explained by functional non-SNP causal variants, which may have broader implications for other cancers GWAS. More generally, our results underscore the importance of investigating other classes of inherited variation as causal mediators of human traits.

## Abstract (web summary)

Here, the authors functionally characterize a complex genetic variant relevant in prostate cancer that regulates IRX4 expression through epigenetic activation. This work highlights the significance of non-single nucleotide polymorphism causal variants in explaining disease risk.

## Introduction

Genome Wide Association Studies (GWAS) have identified thousands of risk loci across a variety of human traits including prostate cancer (PCa). To date, ~150 prostate cancer risk loci have been identified at genome wide levels of significance (*p* < 5 × 10^−8^)^1^. The vast majority of risk-associated variants are located in non-protein coding regions, complicating the mechanistic understanding of these variants because there is no genetic code for the non-coding genome. Imputation and genetic fine mapping are often integrated with epigenetic features as first steps towards prioritizing candidate causal variants. These candidate risk variants can undergo functional evaluation using genome editing technology to establish a causal role in the trait^2–4^.

To date, most GWAS studies have focused on SNPs due to their high prevalence and the technical feasibility in measuring genotypes. While SNPs can be assayed in a simple and high throughput manner to obtain highly accurate genotypes, accurate genotyping and functional characterization of complex polymorphisms remained challenging. The annotation and assessment of the biological significance of other classes of polymorphisms, including insertions and deletions (INDELs), multiple-nucleotide variants (MNVs), and multiple nucleotide length polymorphisms (MNLPs) has proven more challenging. Resequencing data from published studies showed that INDEL variants (1–100 bp) constitute up to 18% genetic polymorphisms^5–7^ and, importantly, over 90% of these variants were confirmed by independent studies. Greater than 99% of these variants localize to the non-coding genome and the functional contribution of these variants to human disease remains unknown^8–12^.

A recent study demonstrated that somatic INDELs are among the least well-characterized genetic variants due to challenges with interpreting short-read DNA sequences^13^. Detailed sequence analysis of epigenetically active regions from 102 different cell lines combined with advances in computational analyses such as multiple DNA alignment algorithms revealed that INDEL variants in the non-coding genome have the potential to form active enhancers and influence oncogene activity. Other recent studies revealed the difficulties of identifying MNVs due to the miss annotation and lack of comprehensive computational approaches^14–16^.

A study conducted by Jiang et al. analyzing the bovine genome has demonstrated the existence of MNLPs, which involve variations of 5–18 nucleotides in length and exhibit low sequence identity and different promoter activities between alleles in the UCN3 and CRHR2 genes^17^. The discovery of MNLPs adds to the complexity of mammalian genomes and has the potential to impact their evolutionary, functional, and phenotypic features. In a relevant research, Nguyen et al. suggest that MNLPs are a novel class of genetic polymorphism that may have important biological implications in the human genome as well^18^.

Several lines of evidence have demonstrated that trait-associated variants are enriched in cis-regulatory elements, which influence the expression of nearby or distant target genes^19–22^. This observation leads to the hypothesis that trait-associated variants alter transcription factor (TF) binding and chromatin signals that ultimately impact target gene expression. Based on this framework, it has become de rigueur to intersect candidate causal variants with epigenetic marks to prioritize polymorphisms for functional evaluation^23^. However, it is not uncommon for risk loci to show no overlapping epigenetic features, suggesting that there are alternative genetic mechanisms underlying disease risk in these regions. One such locus is the 5p15.33 cytogenetic region, where the regions associated with prostate cancer risk localizes to a small (~6 kb) region of linkage disequilibrium containing six strongly correlated candidate causal variants 7 kb upstream of the *IRX4* promoter^24,25^. Multiple studies show that this region exhibits one of the strongest expression quantitative trait locus (eQTLs) associations with the IRX4 TF as a candidate target gene in prostate tissue^18,26,27^.

In the current study, we implicate a previously reported MNLP^18^ as a causal PCa risk variant and show that INDELs or MNLPs are candidate causal variants at two additional loci. These results demonstrate the importance of considering other classes of polymorphisms for explaining the functional mechanisms underlying trait-associated loci discovered through GWAS.

## Results

### Identification of a risk associated MNLP with epigenetic activity

**Fig. 1:**
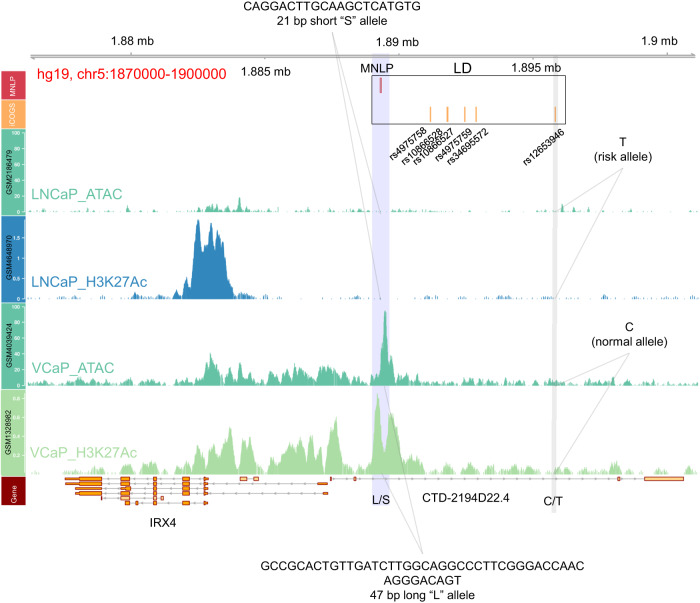
A germline biallelic MNLP variant associated with active epigenetic marks in a genotype dependent manner in human PCa cell line samples. Annotated 5p15.33 PCa risk and *IRX4* genomic locus (hg19, chr5:1870000-1905000). Top track (red) indicates location of the MNLP variant. Orange track indicates six GWAS SNPs significantly associated with PCa risk. The black rectangle containing the 6 SNPs and the MNLP indicates the linkage (LD) among these variants. The 3rd (teal) and 4th (aqua) tracks show chromatin accessibility (ATAC-seq) and H3K27ac ChIP-seq signals from the LNCaP PCa cell line, respectively. These tracks represent the S MNLP variant linked to the T risk allele at the rs12653946 leading SNP (highlighted in gray). The 5th (teal) and 6th (light green) tracks display chromatin accessibility and H3K27ac signals from the VCaP PCa cell line, corresponding to the L MNLP allele and the C protective allele at rs12653946, respectively. The bottom track indicates the physical positions of *IRX4* and *CTD-2194D22.4*. Notably, only the presence of the L allele is associated with epigenetic activity (highlighted in blue). The sequences of the S and L alleles are indicated at the top and bottom, respectively. Data source for tracks 3–6 are listed in Supplementary Data 5.

GWAS identified rs12653946 as the most significantly associated SNP at the chromosome 5p15.33 prostate cancer (PCa) risk locus^1,25,28^. This variant is an eQTL for the *IRX4* gene where the T risk allele is significantly associated with lower *IRX4* expression and increased risk of prostate cancer^18,26,27^. Rs12653946 is in linkage disequilibrium (LD) with five other SNPs; together this set of 6 SNPs represents all the plausible candidate causal variants that would be identified using current post-GWAS functional approaches (Fig. 1). However, none of these variants intersect epigenetic features in PCa cell lines, including LNCaP and VCaP (Fig. 1). A prior study identified a novel MNLP, which is in LD with the above listed SNPs (Fig. 1) and showed the strongest association with PCa susceptibility in a Japanese population (*p* = 2.18 × 10^−11^)^18^. This variant has two alleles: a 21 bp short and a 47 bp long allele (hereafter designated as “S” and “L” allele from here on) which correspond to two previously annotated polymorphisms, rs745614767 and rs386684493, respectively (Fig. 1 and Supplementary Fig. S1a). Notably, the L allele displays open chromatin regions as determined by transposase accessible chromatin sequencing (ATAC-seq) and an active state as shown by H3K27ac chromatin immunoprecipitation (ChIP-seq) signals (Fig. 1). The S allele does not possess epigenetic activity. These data suggest that the L allele variant possesses regulatory potential not present at the other candidate causal variants.

### Accurate genotyping of the MNLP alleles in PCa cell lines and human samples

We noted that most studies erroneously annotated and genotyped the MNLP region because of its complexity (Supplementary Fig. S1b). Therefore, we genotyped cell lines and human samples to create an accurate reference sequence (Supplementary Data 1) and genotyping platform (Supplementary Methods) for our further analyses. Genotyping the MNLP in four PCa cell lines by amplicon sequencing confirmed the existence of the S and L alleles (Supplementary Fig. S1c). This analysis revealed, that LNCaP is homozygous for the S allele, VCaP and PC-3 are homozygous for the L allele, and 22Rv1 is heterozygous (Supplementary Fig. S1d). We performed deep amplicon sequencing in pooled human germline samples (*n* = 62) and in an additional cohort of 56 individual clinical samples (Fig. 1e, f) (“Methods” section); these analyses confirmed the existence of a single biallelic MNLP variant (Supplementary Fig. S1a) with L and S alleles in the human population.

### Genotyping the MNLP in TCGA samples

Using the S and L allele-specific reference genomes (Supplementary Data 1), we genotyped this region in 1310 TCGA germline samples. As expected, the MNLP is linked with the rs12653946 SNP with an LD value of (*D*′ = 0.72, *r*^2^ = 0.76; Supplementary Fig. 2a, b). Notably, the C protective allele of rs12653946 correlates with the L allele whereas the T risk allele correlates with the S allele of the MNLP (Fig. 1 and Supplementary Fig. 2c). These data suggest that the L allele may confer a protective effect against the development of prostate cancer.

### Recombinant individuals implicate the MNLP as the causal eQTL for *IRX4* expression

**Fig. 2:**
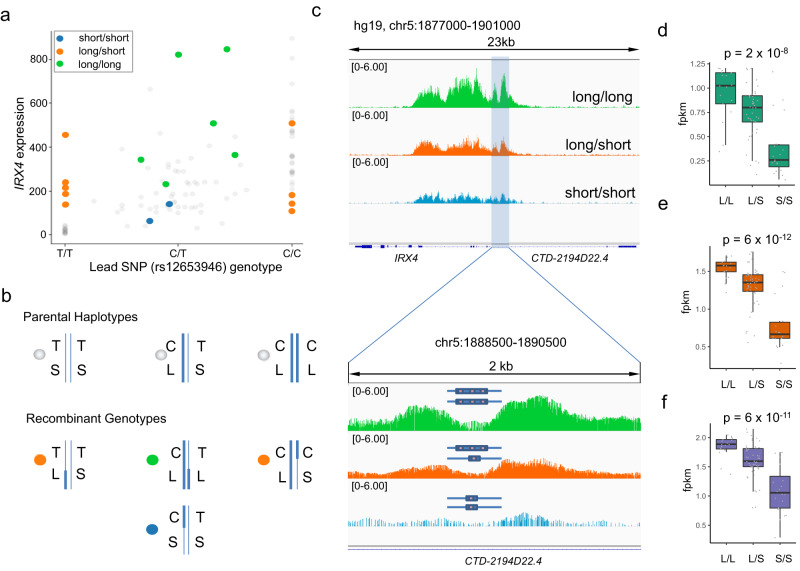
Germline complex variant (MNLP) regulates *IRX4* expression. **a** Categorical scatter plot showing correlation between SNP genotype, complex variant genotype and *IRX4* expression levels in TCGA PRAD samples (*n* = 121). All non-recombinant samples (*n* = 103) marked by transparent gray dots. The following recombinant (*n* = 18) cases were identified and color coded: heterozygous MNLP (L/S=orange) with homozygous SNP (T/T (*n* = 6) or C/C (*n* = 4)) and homozygous MNLP (L/L=green) (*n* = 6) or (S/S=light blue) (*n* = 2) with heterozygous SNP. Note, some dots may overlap, see recombinant categories on Supplementary Fig. S2b. Deeper explanation of the dots color codes are shown on panel **b**. **b** Homozygous and heterozygous parental haplotypes (without recombination), indicated by gray dots (upper panel). Bottom panel shows the possible recombinant genotypes (recombination between MNLP and rs12653946 index SNP), labeled by orange, blue, and green dots. Blue lines are illustrating one or the other haplotypes and the existing recombination events between MNLP and rs12653946 index SNP. **c** Aggregated H3K27ac signal plot from human prostate tumor samples (*n* = 27) at the MNLP position across 3 genotypes. The MNLP region indicated by vertical light blue highlight, genotypes are color coded; L/L = green (*n* = 6), L/S = orange (*n* = 18), and S/S=light blue (*n* = 3). Human tissue data shows genotype-dependent chromatin activity at the MNLP region, the presence of L allele associates with higher H3K27ac signal. **d** *IRX4* expression levels in the function of MNLP genotypes using the GSE130408 data set. **e** H3K27ac ChIP-seq signal intensitieat chr5:1888500-1890500 (hg19) in the function of MNLP genotypes using the GSE130408 data set. **f** AR ChIP-seq signal intensities at chr5:1888500-1890500 (hg19) in the function of MNLP genotypes using the GSE130408 data set. For boxplots (panel d-f), lower and upper hinges indicate 25th and 75th percentiles; whiskers extend to 1.5 x the inter-quartile ranges (IQR). *P* values are for Pearson correlation between fpkm and genotype. Data source for panel **c** and **d**–**f** is listed in Supplementary Data 5.

Next, we investigated the relationship between germline MNLP status and *IRX4* expression levels using paired prostate samples (PRAD cohort, *n* = 121) from TCGA. Each sample was genotyped at the rs12653946 and the MNLP statistics were determined based on sequencing coverage patterns which resulted nine possible genotype combinations (Supplementary Fig. 1f, g). We observed 7 out of the 9 possible genotype categories. Out of the 121 prostate samples, 18 displayed recombination events between the MNLP and rs12653946 position (Fig. 2a, b and Supplementary Fig. S2b). The recombinant individuals allowed us to isolate the effects of the SNP and MNLP on *IRX4* expression. For example, the non-recombinant homozygous T/T variants are associated with low expression of *IRX4*^18,26,27^. However, in recombinant T/T individuals harboring the L MNLP allele, *IRX4* expression was elevated compared to non-recombinant individuals (Fig. 2a). The recombinant individuals demonstrated that the MNLP status has a stronger impact on *IRX4* expression levels than rs12653946 (Fig. 2a and Supplementary Fig. S2d, e).

### Differential epigenomic activity among MNLP genotypes

Since the MNLP variant is strongly correlated with *IRX4* levels, has epigenetic activity, and lies outside of the *IRX4* promoter, we posited that it was an enhancer. To assess enhancer activity in this region, we re-analyzed our H3K27ac ChIP-seq data derived from 26 prostate cancer samples^29^. The results revealed that the L MNLP allele (in LD with the protective C allele) is associated with greater H3K27ac signal compared to the S allele (in LD with the T risk allele; Fig. 2c and Supplementary Fig. 2f, g). Using an independent dataset from our previous work (GSE130408), we demonstrated and quantitated the allele specific IRX4 expression, H3K27ac signal, and AR binding^30^. We observed increased IRX4 expression, higher H3K27ac signal, and stronger AR binding in the presence of the L allele (Fig. 2d–f). To demonstrate allele-specific chromatin activity, we analyzed raw data of a representative heterozygous (L/S) sample at the MNLP position. We demonstrated approximately ten times enrichment of the L allele-specific reads compared to the S allele reads from H3K27ac ChIP-seq data (Supplementary Fig. 2h–j). These results suggest a model whereby the L allele creates an enhancer that positively regulates *IRX4* expression.

### Genome and epigenome editing confirm that the MNLP is an *IRX4* enhancer

**Fig. 3:**
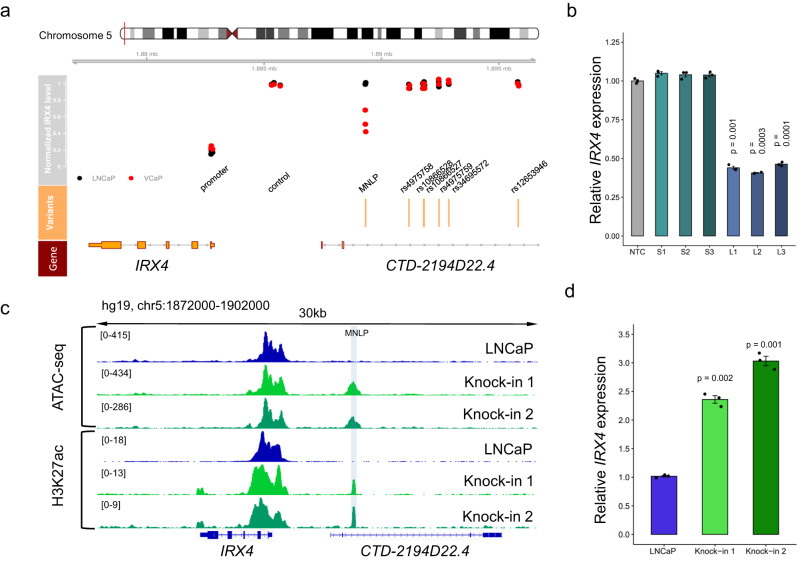
Functional evaluation of the MNLP and correlated SNP variants by CRISPRi reveals the L allele regulatory effect on *IRX4* level and L allele knock-in in LNCaP cells generates an active enhancer for *IRX4*. **a** Annotated 5p15.33 PCa risk region and *IRX4* genomic locus (hg19, chr5:1870000-1905000). Bottom track (red) shows *IRX4* genomic region, middle track (orange) marks the correlated SNPs and MNLP positions. Upper track (gray) shows the mRNA expression levels of *IRX4* after CRISPRi, mediated inhibition of indicated variants; each dot represents a unique gRNA with black dots indicating *IRX4* expression in LNCaP (homozygous for S allele) and red dots indicate expression in VCaP (homozygous for L allele). To control CRISPRi inhibition effect on the *IRX4* expression, three *IRX4* promoter targeting gRNA were used as a positive control. As a negative control, three guide RNA were used ~3 kb upstream at the promoter region. Guide RNA locations and individual measurements of all three replicates are listed in Supplementary Fig. S3. **b** CRISPRi experiment in PC-3/*AR* PCa cell line demonstrates the L allele regulatory role in IRX4 expression. Suppressing the L allele by specific gRNAs showing ~ two-fold decrease on the IRX4 level. The suppression experiments were independently repeated three times (*n* = 3), and the average values are shown on the bars, while individual values are represented by dots. Error bars indicate the standard deviation of the three biological replicates. A two-sided t-test was used to calculate statistical significance. **c** Genome editing of LNCaP cells using HDR leading to knock-in of the L allele variant. H3K27Ac CHIP-seq at *IRX4* genomic locus (hg19, chr5:1872000-1902000) in two individual knock-in clones and parental LNCaP cells. Light blue highlighted are shows the enhancer position. Knock-in 1 and Knock-in 2 clones both carrying a single copy of the L allele confer high epigenetic activity both measured by ATAC-seq and H2K27ac ChIP-seq in this region compared to the parental cell line (lack of L allele). **d** *IRX4* mRNA expression levels in LNCaP parental and two knock-in clones. Knock-in 1 and Knock-in 2 clones both carrying a single copy of the L allele showing 2.4- and 3-fold *IRX4* level increase compared to the parental cell line (lack of L allele). Bars represent the IRX4 expression levels using three technical replicates (individual dots) from each clone and control samples. A two-sided *t*-test was used to calculate statistical significance, error bars represent standard deviation.

To functionally interrogate the six SNPs and the MNLP variant, we suppressed regulatory activity at these locations using CRISPRi technology and determined its impact on *IRX4* expression (Supplementary Fig. 3 and Supplementary Data 6). Targeting the L allele using CRISPRi technology in the homozygous VCaP cell line significantly decreased *IRX4* expression by ~50% whereas suppressing the SNPs had no effect (Fig. 3a and Supplementary Fig. 3a). Targeting the S allele with CRISPRi in the homozygous LNCaP cell line had no effect on *IRX4* expression (Fig. 3a and Supplementary Fig. 3a). To further confirm enhancer activity, we repeated the CRISPRi experiment with PC-3/AR cell line which is homozygous for the L allele and stably expressing AR. Consistently with the previous results, we observed IRX4 suppression using the L allele targeting gRNAs and no effect with the S allele targeting gRNAs (Fig. 3b). These data indicate that the L allele variant increases *IRX4* expression by increasing enhancer activity.

### Allelic knock-in of the L allele increases chromatin accessibility and *IRX4* expression

To directly test the impact of the L allele on enhancer activity, we precisely introduced the L allele by homology-directed repair (HDR) into LNCaP cells and created isogenic cell lines using the CAUSEL pipeline^3^. Two independent LNCaP clones, each carrying one copy of the L allele, were generated (Supplementary Fig. S4). Compared to the parental LNCaP line, clones carrying the L allele position had significantly increased epigenetic activity, as measured by ATAC-seq and H3K27ac ChIP-seq, respectively (Fig. 3c). Consistent with these results, *IRX4* gene expression levels were increased by up to 3-fold in engineered clones (Fig. 3d). Using our isogenic cell lines, these data demonstrate that the L allele causally induces increased epigenomic and transcriptional activity. Deep amplicon sequencing verification confirmed the correct L allele integration, no additional off-target mutations were observed in the isogenic clones (Supplementary Fig. S4b).

### AR is a key regulatory TF at this locus

Increased binding of pioneer factors and TFs promotes chromatin accessibility and recruitment of chromatin-modifying enzymes to enable gene regulation. We sought to identify which *trans-*acting factor bound to the L allele (Fig. 1) to regulate *IRX4* expression. The Cistrome data browser (Cistrome DB), a compendium of epigenetic datasets, showed that eight prostate-relevant candidate TFs may bind to the coordinates spanning the MNLP variant^31,32^. We observed ERG, AR, FOXA1, GABPA, ETV1, NR3C1, MYC, and HOXB13 binding to this region in VCaP (homozygous for the L allele) (Supplementary Fig. S5a). The S allele has a predicted ETV1 binding site. This prediction was confirmed showing ETV1 binding in the MNLP region in the LNCaP cell line (homozygous for the S allele) (Supplementary Fig. S5b, c).

**Fig. 4:**
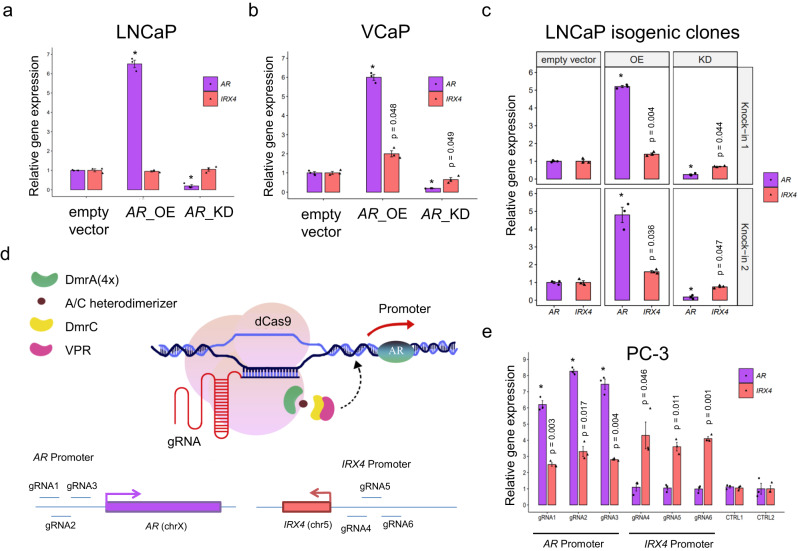
L MNLP variant modulates *IRX4* expression by encoding a functional AR binding site. **a** *IRX4* and *AR* mRNA expression levels were assessed in LNCaP cells (S MNLP variant) through RT-PCR following transient overexpression of *AR* cDNA (*AR*_OE) and knock-down of *AR* using shRNA (*AR*_KD), compared to an empty vector control. Modulating the *AR* level did not have any significant impact on *IRX4* expression. **b** *IRX4* and *AR* mRNA expression in VCaP (L MNLP variant) cells following transient overexpression *AR* cDNA and shRNA-mediated knock down of *AR* by RT-PCR. Altering the *AR* level has influenced *IRX4* expression. **c** *IRX4* and *AR* mRNA expression in L allele knock-in LNCaP clones following transient overexpression AR cDNA and shRNA-mediated knock down of AR by RT-PCR. After introducing the L allele in LNCaP cells manipulation of the AR level has influenced *IRX4* expression. **d** Schematic showing CRISPRa regulatory model and experimental design to modulate *IRX4* level by altering the AR level in PC-3 cells homozygous for the L MNLP variant. For AR activation, CRISPRa is recruited by three different gRNAs targeting the AR promoter. For a control experiment, *IRX4* promoter was targeted by three different gRNAs. All results were calculated by data normalized to non-human targeting gRNA. **e** *IRX4* and *AR* mRNA expression in PC3 (AR-) cells following CRISPRa using indicated gRNAs by RT-PCR. Data expressed as mean of three biological replicates. Error bars represent standard deviation. All experiments (overexpression, knock-down and activation, panel **a**–**c** and **e**) were repeated three times independently (*n* = 3), with the average values indicated on the bars and individual values represented by dots. Error bars indicate the standard deviation of the three biological replicates. A two-sided *t*-test was used to calculate statistical significance. *p < 0.05.

In order to experimentally identify *trans-*acting factors driving *IRX4* transcription at this *cis-*element, we performed transcription factor knock down (KD) and overexpression (OE) of candidate TFs in the modified clones. Five TFs were selected based on the Cistrome DB analysis outlined above (Supplementary Fig. S5a); while ERG is the top candidate, LNCaP and normal prostate do not express ERG, therefore it was not evaluated in these analyses. We observed that AR OE significantly activated *IRX4* expression, conversely AR KD suppressed it in VCaP parental cell line and modified L LNCaP clones, but not the LNCaP parental cell line (Fig. 4a–c). Manipulation of *HOXB13*, *FOXA1*, *ETV1*, and *NKX3-1* expression in LNCaP parental cell line, LNCaP L allele knock-in clones and VCaP cell line had no measurable impact on the *IRX4* expression (Supplementary Fig. S6).

**Fig. 5:**
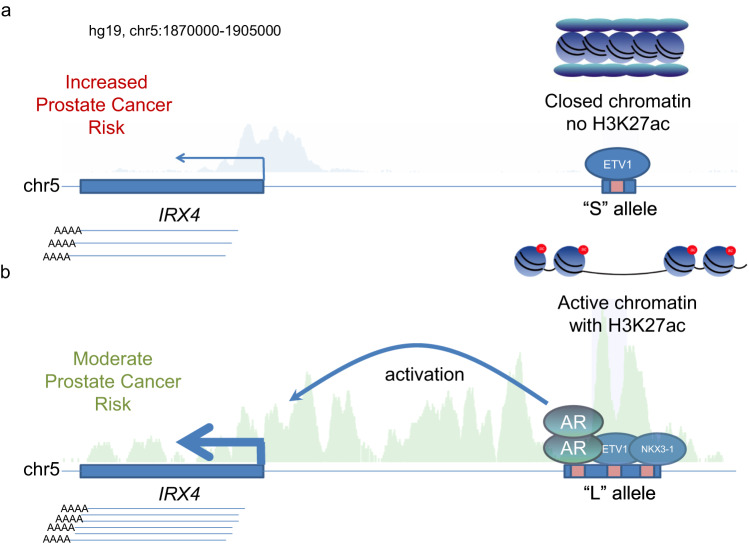
Regulatory model at the chromosome 5p15.33 PCa risk region. Visualization of the *IRX4* genomic region (hg19, chr5:1870000-1905000) and demonstration of the molecular background of the genotype-dependent *IRX4* regulation. **a** The S allele has no regulatory effect on *IRX4* level. ETV1 binds here, but this binding alone is not able to open the chromatin and initiate enhancer activation at the MNLP position. Therefore, *IRX4* transcript level remains at basic level due to the promoter activity. This condition has higher susceptibility for PCa. **b** In the presence of the L allele, elevated *IRX4* transcript level can be observed. AR binding initiate enhancer activation at the MNLP position which leads to elevated *IRX4* transcript level. This condition has lower risk to develop PCa.

These results indicated that the AR is a key regulator which directly influence *IRX4* expression. Supporting this hypothesis, androgen stimulation induced *IRX4* levels whereas AR antagonists decreased *IRX4* levels in VCaP cells (GSE135879)^33^ (Supplementary Fig. S7a). PC-3 is an AR negative cell line that is homozygous for the L allele (Supplementary Fig. S1d). ChIP-seq data from AR overexpressing PC-3 cells showed evidence of AR binding at the L allele^34^ (Supplementary Fig. S7b). To further validate the functional importance of the AR-IRX4 axis, we used CRISPR activation (CRISPRa)^35^ to upregulate AR in the PC-3 cell line by targeting the AR promoter with dCas9 fused to the VP64 transcriptional activator (Fig. 4d), which led to concomitant induction of *AR* and *IRX4* expression (Fig. 4e). Of note, CRISPRa of the *IRX4* promoter increased *IRX4* transcriptional levels without impacting *AR* expression. These data indicate that AR directly drives *IRX4* expression via enhancer activation through binding to the L allelic variant (Fig. 5). Modulating the AR level is evidently influencing *IRX4* level in the presence of the L allele (Fig. 4c and Supplementary Fig. S7a).

### *IRX4* functional analysis

Next, we analyzed cancer phenotypes as a function of *IRX4* level. *IRX4* is a tissue-specific TF that expresses in skin, esophagus, prostate, heart, and breast at relatively low expression level (Supplementary Fig. S7c). To investigate whether *IRX4* functionally impacts cell proliferation, we altered its expression in LNCaP cell line. Cell proliferation assays showed that *IRX4* expression level did not influence cell proliferation in LNCaP cells (Supplementary Fig. S7d, e). As an alternative approach, we performed competition assays between cell lines with varying expression levels of *IRX4* and the parental LNCaP cell population. These experiments confirmed that altered *IRX4* expression had no significant impact on cell growth in vitro (Supplementary Fig. S7d, e).

We further examined what cellular processes are affected by *IRX4* using RNA-Seq analysis (“Methods” section). We identified; 197 differentially expressed genes (52 up and 145 down) in the control vs. KD comparison, 85 differentially expressed (48 up and 37 down) in the control vs. OE comparison and 241 differentially expressed genes (154 up and 87 down) in the KD vs. OE comparison (Supplementary Fig. S8a–c and Supplementary Data 2a–c). Gene expression profiling by RNA-Seq revealed that *IRX4* overexpression induces developmental, maturation, and differentiation processes along the different (CTRL vs. OE, CTRL vs. KD and KD vs. OE) comparisons using gene ontology (GO) analysis. To further analyze the effect of IRX4 level manipulations, we determined the sequentially altered genes along the three conditions (KD > CTRL > OE and KD < CTRL < OE). We identified 187 sequentially altered genes (Supplementary Fig. S8d and Supplementary Data 2d), from which 37 showed significant alteration (*p* < 0.05 and logFC > |1 |) between the KD vs. OE comparison (Supplementary Fig. S8e and Supplementary Data 2e). Gene ontology (GO) analysis confirmed the developmental role for both increasing and decreasing gene sets (Supplementary Fig. S8f, g). These data may help to guide future functional experiments.

### Other PCa risk loci harbor complex variants as candidate causal variants

**Fig. 6:**
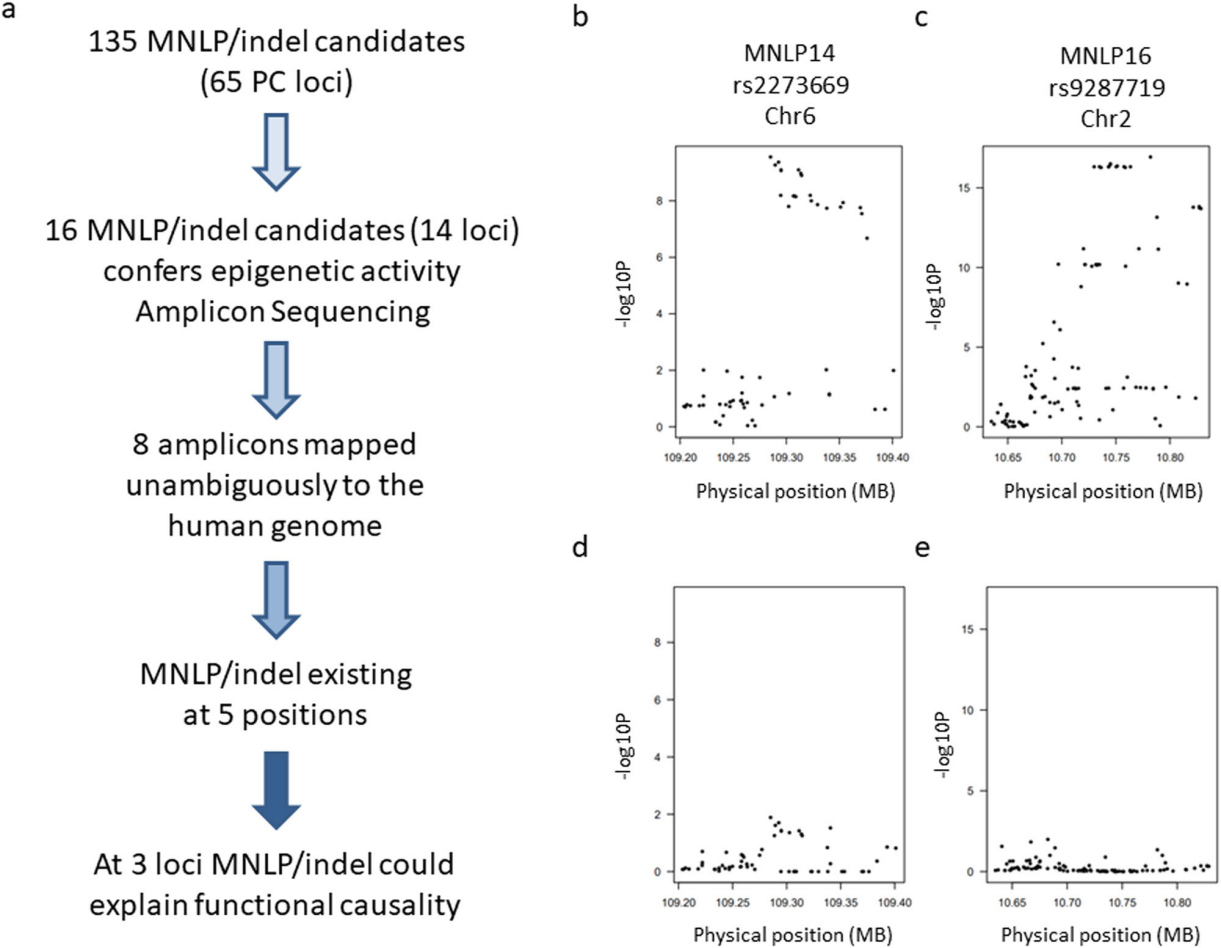
Systematic analysis of complex variants revealed two potential loci where novel correlated complex variant could explain PCa risk. **a** Potential functional correlated complex variant identification pipeline based on coverage-based analysis of TCGA samples. **b** GWAS Manhattan plot of the rs2273669 related PCa risk region on chromosome 6. **c** GWAS Manhattan plot of the rs9287719 related PCa risk region on chromosome 2. Genome-wide significant associations indicated by individual dots above *P* = 5 × 10^−8^. **d** GWAS data MNLP conditioning neutralizes the rs2273669 effects, demonstrating the MNLP14 genotypes power. **e** GWAS data MNLP conditioning neutralizes the rs9287719 effects, demonstrating the MNLP16 genotypes power. GWAS identified correlated SNPs explain PCa risk at genome-wide significance showing 2 different risk regions (**b**, **c)**. Conditioning GWAS associations in the locus on the corresponding identified correlated complex variant in the risk region explained all significant GWAS associations (**d**, **e**), supporting the potential causal variant role of the correlated complex variant. P values indicated for different approaches (Supplementary Fig. S9j).

Similar to the *IRX4* locus, we hypothesized that complex variants may explain disease risk at other PCa risk loci. Using our computational pipeline (see methods) we analyzed 146 PCa risk loci^1^ to identify candidate complex variants (“Methods” section, Supplementary Methods). We identified 135 computationally predicted complex variants belonging to 65 different PCa risk regions (Supplementary Data 3). We selected 16 candidate complex variants for further validation by deep amplicon sequencing (Supplementary Data 4 and S6) belong to 14 PCa risk loci and identified 3 loci, where complex variant could explain functional causality (Supplementary Fig. S9). Nine out of the 16 amplicons mapped unambiguously to the human reference genome (Supplementary Fig. 10) and 5 of those sequenced regions showed biallelic complex variants, whereas the remaining 4 regions contained SNPs or no genetic variants. We built a new reference genome for these 5 candidate complex variants and genotyped TCGA samples by realignment (see Supplementary Methods for the details). In addition to the *IRX4* locus (MNLP3), we observed two loci, one on chr6 (rs2273669) and one on chr2 (rs9287719) where disease risk was associated with an epigenetically active correlated complex variant (MNLP14 and MNLP16, respectively) (Fig. 6). Amplicon sequencing confirmed deletion allele sizes; 27 bp (MNLP3), 10 bp (MNLP14), and 25 bp (MNLP16) (Supplementary Fig. S9a–c). Correlations between the leading SNP genotype and complex variants at the corresponding locus are shown in (Supplementary Fig. S9d–f). Using Cistrome db data sets, epigenetic analysis revealed, that MNLP3 and MNLP16 has prostate relevant TF binding (AR, FOXA1) at the complex variant region and all three complex variants showed MYC binding (Supplementary Fig. S9g–i).

To investigate if the identified novel complex variants could account for the PCa risk association signal, we trained predictive genetic models for each complex variant and inferred the expected association with PCa risk using summary data from a recent large association study (see “Methods” section). This approach is conceptually similar to Transcriptome-Wide Association Studies (TWAS) where the predicted phenotype is the complex variant genotype rather than gene expression^36^. All five complex variants yielded significantly accurate predictive models, as assessed by cross-validation of the predictor, and were predicted into the PCa GWAS data. Three out of the five complex variants achieved genome-wide significant associations in the PCa GWAS – Del3 (*P* = 3e-23), Del14 (*P* = 6e-09), and Del 16 (*P* = 5e-17) – and were statistically equivalent to the top GWAS SNP. Separate statistical analyses^37^ confirmed colocalization between the complex variant and the GWAS variant for all three complex variants (probability of colocalization > 0.90), evidence that the GWAS association and in the complex variant are explained by the same causal genetic mechanism (see “Methods” section). Thus, these complex variants are leading candidate causal variants for these loci.

## Discussion

GWAS determine statistical associations between genomic variants and phenotypes. While single nucleotide polymorphisms (SNPs) are most commonly assayed, many classes of polymorphisms exist in the human genome – from single nucleotide polymorphisms to complex variants and Megabase size copy number variations^38^. SNPs can be accurately identified by the widely used shotgun short-read sequencing approaches, however other variant classes such as complex variants, especially in the range of 50–100 bp, often cannot be resolved reliably by this technology^39^. In fact, up to 18% of human germline diversity may consist of complex variants of <100 bp. A recent study indicated that germline genomic structural variants (SVs) may be the causal variant for at least 3.5–6.8% of eQTLs^40^. Estimating the true impact of germline SVs, such as complex variants in the range of 10–100 bp, will require the accurate evaluation and functional annotation of the genome^41^. If a SNP is in linkage disequilibrium (LD) with a difficult-to-detect polymorphism that is driving the trait, then causal variant identification will be problematic.

The PCa risk SNP, rs12653946, along with other correlated SNPs, is a strong eQTL for *IRX4* expression levels in prostate tissue^18,26,27^. Intersecting epigenetic data with trait loci often indicate functional *cis-*regulatory elements^13,24^. However, in this case, no chromatin features colocalized with any SNP (Fig. 1). This risk locus, 5p15.33, has been recently resequenced^18^ by Sanger sequencing and revealed that this region harbored a biallelic complex variant (21 bp and a 47 bp) located 6.5 kb from the top risk variant SNP. The dbSNP Build 151 (2017) database described 44 different polymorphisms (19 complex variants and 25 SNPs) at the region encompassing the MNLP position thus highlighting complexity in annotating polymorphisms in this region (Supplementary Fig. S1b). After resequencing this region in four PCa cell lines, a pooled Coriell sample set (NA13405DNA) and individual clinical samples, we also observed only two alleles (21 bp S and a 47 bp L), and no evidence of other SNPs in this area (Supplementary Methods). Consistent with our hypothesis that the MNLP region was erroneously annotated, the most recent dbSNP Build 155 (2021) indicates only two variants (rs530534670 and rs199577062) in this region (Supplementary Methods Fig. SM1a). We further suspected that rs530534670 and rs199577062 may serve as proxies for the MNLP genotypes, but the true polymorphism is the MNLP with the L and S alleles^42^. We proved by genotyping of 1000 Genome Project samples and a nucleotide level analysis that rs530534670 and rs199577062 are part of the L and S alleles and serve as surrogates for the S and L allele genotypes. We provided in-depth explanation for this phenomenon in the Supplementary Methods section.

In contrast to the SNPs, multiple chromatin features overlapped the complex variant. The two MNLP alleles showed markedly different epigenomic activity by H3K27ac chromQTL analysis (Fig. 2c). CRISPR interference (CRISPRi) of the L allele in the VCaP prostate cancer cell line (homozygous for the L allele) reduced *IRX4* expression (Fig. 3a) and introducing the L allele into a cell line only carrying S alleles opening the chromatin region and the enhancer activation (increased H3K27ac signal) increase *IRX4* expression (Fig. 3c, d). In contrast, CRISPRi at the SNPs did not influence *IRX4* expression (Fig. 3a and Supplementary Fig. S3). We further elucidated that the complex variant variants regulate *IRX4* expression through the AR TF (Fig. 4).

We also investigated whether complex variants could explain the increased risk of other PCa risk loci identified by GWAS studies (Fig. 6 and Supplementary Fig. S10). We identified three risk regions for which fine-mapped SNPs did not overlap with any TF ChIP-seq data from the Cistrome data base. In addition, we found two regions where the top SNP showed no epigenetic activity, but they were highly correlated with a complex variant showing epigenomic activity. These data suggest that other PCa risk loci may exist where a correlated complex variant is a plausible functional variant.

Our computational and sequence analysis confirmed and validated that all three complex variants (Del3, Del14, and Del16) are biallelic (Supplementary Fig. S10a–f), Overlapping analysis with dbSNP151 entries revealed, that these complex variants are overlapping with many previously reported genetic variants (Supplementary Figs. S1a and S10e, f, and Supplementary Data 3 and S4). These are presumably rare or poorly annotated variants that require further investigation.

While we clarified the functionally causal *cis-*regulatory mechanism of *IRX4* expression (Fig. 5), the biological relevance of this gene in driving prostate carcinogenesis remains incomplete. *IRX4* has been considered a putative tumor suppressor based on the observation that the SNP risk locus is associated with lower *IRX4* expression. Furthermore, Nguyen et al. found that suppressing *IRX4* expression leads to increased proliferation in LNCaP cells. We could not confirm this observation, which may be due to either differences in cell biological manipulations or the fact that we used a different clone of the LNCaP cells^43^. Our findings are consistent with those reported in the Depmap (https://depmap.org/portal/). Proliferation only measures a single cancer-related phenotype so, if *IRX4* is involved in tumorigenesis, it could be acting through mechanisms other than proliferation. Since *IRX4* is known to play an important role in cell differentiation^44^, prostate tissue-dependent manipulation in transgenic animals may provide more relevant information about the role of this gene in prostate tumorigenesis.

This work represents one of the first examples of describing a functionally causal complex variant at a GWAS risk locus. Our strategy can be applied for the investigation of other risk loci. Using our preliminary analysis, we estimated 5% (3 associated INDELs/67 tested loci) as a lower bound of the PCa risk loci that may have functional correlated complex variants. Long-range sequencing methods are currently used to improve the annotation of the human genome^39^. Combining sequencing data from more sensitive platforms that can accurately detect more complex polymorphisms will be essential to identifying the full breadth of functionally relevant variants in the genome. Once these updated human genome annotations become available, it will be important to revisit risk loci to investigate if other variant classes can account for the causal mechanism. High confidence genomic variants could be integrated with epigenomic data and functional hypotheses of risk loci could be updated accordingly as we have presented in this paper.

## Methods

### Publicly available data used in this study for data visualization

All publicly available data used in this study are listed in Supplementary Data 5.

### Sanger Sequencing of human prostate cancer cell line

Genomic DNA was isolated from LNCaP, VCaP, 22Rv1, and PC-3 prostate cancer cell lines using Mini Elute DNA kit (Qiagen) according to the manufacturer’s instruction. Hundred nanogram of each DNA samples were amplified using high fidelity (Phusion DNA polymerase, Thermo Fisher Scientific) DNA polymerase in 50 ul final reaction volume using 500 nM per each o458 and o459 oligonucleotides (Supplementary Data 6). PCR products were separated on 2% agarose gel. Corresponding fragments (Supplementary Fig. S1c) were purified (Monarch DNA Gel Extraction Kit, New England Biolabs) and submitted for Sanger sequencing service (Genewiz, recently Azenta) using o458 and o459 primers separately, to confirm genotypes from both directions. Chromatograms were analyzed and visualized by SnapGene wiever software (Supplementary Fig. 1c, d).

### Next-generation deep amplicon sequencing of pooled germline DNA samples

Pooled germline genomic DNA sample (NA13405DNA, sample pool (*n* = 62), CEPH Collection DNA pool: Amish, Utah and Venezuelan Pedigrees, (males (31) and females (31))) was purchased from Coriell and 100 ng was used to amplify (Phusion DNA polymerase, Thermo Fisher Scientific) the MNLP region using the o460 and o461 (Illumina sequencing platform compatible o458 and o459) oligonucleotide combination (Supplementary Data 6). Amplicons were purified (QIAquick PCR Purification Kit, Qiagen) and sent for deep amplicon sequencing to DFCI-MBCF. Sequencing was performed on Mini-Seq instrument (Illumina), 1 M reads were requested using 150 PE sequencing chemistry. Most frequent read types were determined and analyzed L and S allele frequencies were calculated (Supplementary Fig. S1e).

### Next generation deep amplicon sequencing of individual clinical samples

Germline genomic DNA samples (56) from patients with radical prostatectomy were requested from the Dana–Farber Cancer Institute (DFCI) Gelb Center biobank and database as part of DFCI protocols 01-045 and 09-171 and approved by the DFCI/Harvard Cancer Center institutional review board and ethical committee. Hundred nanogram of each DNA samples were amplified using high fidelity (Phusion, Thermo Fisher Scientific) DNA polymerase in 50 μl final reaction volume using 500 nM per each o458 and o459 oligonucleotides (Supplementary Data 6). Amplicons were purified (QIAquick PCR Purification Kit, Qiagen) and sent for deep amplicon sequencing to DFCI-MBCF. Sequencing was performed on Mini-Seq instrument (Illumina) using 150 PE sequencing chemistry. Each amplicon was barcoded and fastq files were deconvoluted by DFCI-MBCF. For each amplicon 10.000 reads were requested. Most frequent read types were determined and analyzed L and S allele frequencies were calculated (Supplementary Fig. S1f).

### Cell culture

LNCaP (ATCC Cat# CRL-1740), VCaP (ATCC Cat# CRL-2876), PC-3 (ATCC Cat# CRL-7934), and 22Rv1 (ATCC Cat# CRL-2505) prostate cell lines were requested from ATCC. LNCaP, 22RV1, and PC-3 were cultivated in RPMI-1640 medium containing 10% FBS and 1% pen/strep (Life Technologies), VCaP in DMEM supplemented with 10% FBS and 1% pen/strep (Life Technologies®) Trypsin 0.05%, 0.25%, and 0.5% was used to detach cells from the tissue culture plastic dish. All cells were grown at 37 °C with 5% CO_2_. Cells were passaged a maximum of 20 times. Mycoplasma contamination was checked at least once in a month (PCR Mycoplasma Detection Kit, ABM). Cell line and single-cell clone identities were verified by STR analysis.

### H3K27ac ChIP-seq from human tissue specimens

Fresh-frozen radical prostatectomy specimens were selected from the Dana–Farber Cancer Institute (DFCI) Gelb Center biobank and database as part of DFCI protocols 01-045 and 09-171 and approved by the DFCI/Harvard Cancer Center institutional review board and ethical committee. Areas estimated to be enriched >70% for prostate tumor tissue or normal prostate epithelium were isolated for analysis using hematoxylin and eosin-stained slides from each case reviewed by a genitourinary pathologist. A 2 mm^2^ frozen core was pulverized using the Covaris CryoPrep system. Tissue was then fixed using 1% formaldehyde with methanol for 18 min at 37 °C and quenched with 2 M glycine. Chromatin was sheared using Covaris E220 ultrasonicator into a range of 300–500 bp in size. Sonicated chromatin was incubated overnight with 6 μg of antibody—H3K27ac (Diagenode Cat# C15410196) and bound to protein A and protein G beads (Life Technologies). A fraction of the sample was not exposed to antibody and was used as control (input). IP samples were reverse cross-linked and were treated with RNase and proteinase K. Extracted ChIP DNA was quantified (Qubit fluorometer, Life Technologies) and DNA sequencing libraries were prepared (ThruPLEX-FD Prep kit, Rubicon Genomics). Libraries were sequenced on Illumina platform using 75-bp read technology at Dana-Farber Cancer Institute Molecular Biology Core Facility (DFCI-MBCF).

### H3K27Ac ChIP in LNCaP cells

H3K27Ac ChIP in LNCaP cells was performed as previously described^45^. Briefly, ten million cells were fixed using 1% formaldehyde (Thermo Fisher Scientific) for 10 min at room temperature. Chromatin was sheared in ice-cold lysis buffer (50 mM Tris, 10 mM EDTA, 1% SDS with protease inhibitor) to 300–500 base pairs using the Covaris E210 sonicator. The sample was incubated with 1 μg H3K27Ac antibody (Diagenode, C15410196, Denville, NJ) coupled with protein A and protein G beads (Life Technologies, Carlsbad, CA) at 4 °C overnight. The chromatin was washed with RIPA washing buffer (0.05 M HEPES pH 7.6, 1 mM EDTA, 0.7% Na Deoxycholate, 1% NP-40, 0.5 M LiCl). After decrosslinking, IP DNA as well as its input were extracted using QIAGEN Qiaquick columns, and sequencing libraries prepared using the ThruPLEX-FD Prep Kit (Rubicon Genomics, Ann Arbor, MI). Libraries were sequenced using 75-base pair single reads on Illumina platform at DFCI-MBCF.

### ChIP-seq analysis and data visualization

The ChiLin pipeline 2.0.0^46^ was used for quality control and pre-processing of the data. We used Burrows-Wheeler Aligner (BWA Version: 0.7.17-r1188) as a read mapping tool, and Model-based Analysis of ChIP-Seq (MACS2)^47^ (v2.1.0.20140616) as a peak caller using default parameters using R environment (4.0.1.). The Gviz Bioconductor package was used^48^ for ChIP-Seq signal visualization. TF binding plots were obtained with Toolkit (version 1.0.0) available in Cistrome Data Browser^31^.

### Assay of transposase-accessible chromatin sequencing (ATAC-seq)

ATAC-seq was performed using 50,000 cells of LNCaP parental cell line and L allele knock-in clones each as previously described^49^; 50,000 isolated nuclei underwent tagmentation using the enzyme and buffer from the Nextera Library Prep Kit (Illumina). The tagmented DNA was subsequently purified with the MinElute PCR purification kit (Qiagen), amplified with 10 PCR cycles, and purified using Agencourt AMPure SPRI beads (Beckman Coulter). Library QC and 150 SE was performed at DFCI-MBCF.

### Sample information

Prostate tissue was collected from 27 patients with localized primary prostate adenocarcinoma. H3k27ac chromatin immunoprecipitation sequencing (ChIP-Seq) on these samples, as well as germline SNP genotyping from blood. Germline variants were phased and imputed to the Haplotype Reference Consortium panel. Mapping and aligning were performed using bwa; allele-specific reads were processed according to the WASP pipeline^50^ to remove mapping bias; peaks were identified using the MACS2 software. Allele-specific read counts were generated by the GATK ASEReadCounter^51^.

### Allele-specific analysis

We tested for allele-specific signal using a haplotype beta-binomial test that accounts for read overdispersion. Beta-binomial overdispersion parameters were estimated for each individual/experiment from the aligned allele-specific counts and were found to be consistently low (Normal: mean = 4.90E-04, sd = 0.001350884, *n* = 37; Tumor: mean = 2.66E-03, sd = 0.004844898, *n* = 38). Due to the negligible amount of overdispersion we did not model local structural changes. For each peak and individual, haplotype-specific read counts were merged across all heterozygous read-carrying sites in the peak for a single measure of allele specificity. Every SNP within 100 kb of the peak center and containing at least one heterozygous individual was then tested for allelic imbalance. All heterozygous individuals were tested together under the expectation of a consistent allele-specific effect. Each test was performed once for samples from normal, tumor, both, as well as a differential test between tumor and normal. Finally, peaks were considered imbalanced in each of these four test categories if any of the variants tested for that peak exhibited allele-specific signal at a 10% FDR.

### Transfection

Cells were plated a day before transfection to reach 70–80% confluency at the day of transfection. Cells were transfected with 1 µg of plasmid DNA, or with combinations of plasmid DNA and 100 pM HDR template oligos by 4D-Nucleofector^TM^x Kit (Lonza) using 20 µl Nucleocuvette^TM^ Strips. Cell numbers, buffers, programs and HDR oligo sequences are listed in Supplementary Data 1. Cells were immediately resuspended in 100 µl culturing media and seeded into 1.5 ml pre-warmed culturing media in 24 well tissue culture plate.

### Single-cell cloning

LNCaP cells were filtrated (CellTrics 10um, Sysmex, USA) and plated 3 days after transfection into 20% FBS containing media with 1000, or 2000 cells per 10 cm dish (Corning) previously incubated with FNC Coating Mix® as described by the manufacturer (AthenaES). After 14-28 days, the formed colonies were picked and plated into 384 well tissue culture plate (Corning). After 1–2 days, when the colonies were attached to the plate, they were detached with 0.05% Trypsin (Gibco) and incubated for 2 min at 37 °C. After vigorous shaking and brief centrifugation at 1000×*g* the plate was incubated to regenerate colonies. Media was changed two times weekly on the plates.

### Single-cell clone genotyping

All regenerated clones were subjected for genotype screening by direct PCR method. Cells were detached by adding 20 µl of trypsin per each well and incubated for 2 min at 37 °C, then it was quenched by 40 µl media. Samples were mixed well and 30 µl of cell suspension transferred into 384 well PCR plates, and pelleted by centrifugation for 3 min at 3000 g, and the supernatant removed. Cells were then resuspended in 20 µl lysis buffer (950 µl lysis buffer + 50 µl DNA release solution) Phire Tissue Direct PCR Master Mix (Thermo Fisher Scientific) and denatured for 5 min at 99 °C. In all, 1 µl of cell lysate was directly used for PCR amplifications in 15 µl final volume, using o458 and o459 oligonucleotide combination. PCR products size were analyzed on agarose gel. L allele containing products were further analyzed by deep amplicon sequencing.

### Long “L” allele knock-in and surrounding region verification by sequencing

Two clones were identified with perfect L allele knock-in by deep sequencing analysis. From these cell lysates a 1248 bp region were amplified centered by the MNLP position using Phire Tissue Direct PCR Master Mix (Thermo Fisher Scientific) and o483/o484 oligonucleotide combination (Supplementary Data 1). The correct size of the PCR product was analyzed on agarose gel and the rest of the PCR product was purified (QIAquick PCR Purification Kit, Qiagen) and then subjected for Sanger sequencing from both and using the o483 and o484 in two separate reactions. The presence of intact “tccg” and “gcgtc” “border sequences” (Supplementary Fig. S4b, indicated by gray lowercase letters, right next the MNLP alleles) furthermore correct upstream and downstream sequences were identified confirming the ideal allelic replacement without unwanted genomic alterations.

### Detecting possible off-target effects

Potential off-target events for the S allele targeting gRNA (S2, Supplementary Data 1) were identified using the Cas-OFFinder (http://www.rgenome.net/cas-offinder/) algorithm, allowing 2 bp mismatch, 1 bp RNA-bulge and 1 bp DNA-bulge. In total, 5 possible off-target events were predicted, locating in 3 different chromosomal regions (on chr12, chr13, and chr19). These regions were amplified by (o512/o513, o514/o515, and o516/o517 oligonucleotide combination, Supplementary Data 6) using parental LNCaP and isogenic clones cell lysates, and subjected for Sanger sequencing from both end. The sequencing results confirmed the lack of unwanted genome editing events and intact genomic regions in both L allele knoc-in clones.

### Amplicon sequencing and genotyping of the single-cell clones

Sequencing and genotyping strategy was performed as we previously demonstrated^3^. Direct lysis and amplification were performed of the target regions using Phire master mix and lysis buffer (Thermo Fisher Scientific). Amplicons were barcoded using a second round of PCR. Amplicons were pooled, purified, quantitated, and sequenced by DFCI-MBCF.

### CRISPR/dCas9-mediated repression and gene expression analysis

In order to create stable dCas9-KRAB expressing cell line LNCaP cells were infected with lenti-KRAB-dCas9-blast (Addgene, #89567) and selected with 6 µg/ml blasticidin for two weeks. gRNAs were designed according to the “NGG” protospacer adjacent motive (PAM) restriction and gRNA efficiency score was calculated and ranked. Non-human genome targeting negative control and *IRX4* promoter targeting positive control gRNAs were also selected. gRNA cassettes were synthetized (Integrated DNA Technologies) and cloned into lentiGuide-Puro (Addgene, #52963) vector. All gRNA sequences are listed in Supplementary Data 1. LNCaP cells stably expressing KRAB-dCas9 were then subsequently infected with gRNA vectors and selected with 2 µg/ml puromycin for five days.

Short (S) and Long (L) allele targeting gRNAs were (Supplementary Data) individually cloned into lenti-EF1a-dCas9-KRAB-Puro vector (Addgene #99372), then 3 million VCaP cells were transiently transfected using these constructions (BTX, Harvard Apparatus). After one day regeneration, cells were subjected to puromycin (2 µg/ml) selection. After 72 h antibiotic selection cells were harvested for gene expression analysis.

For CRISPRi experiment in PC-3 cell lines the same method was used as described above for the LNCaP cell lines. Upfront, stable AR expressing PC-3 cell line (PC-3/AR) was created using lenti-CMV-AR-Hygro vector, applying 2 weeks of hygromycin selection (200 µg/ml).

### CRISPR/dCas9-mediated gene activation analysis

For the gene activation experiments 500,000 PC-3 cells were co-transfected with a mixture of 1,000 ng of dCas9-DmrA4x fusions plasmids, 500 ng of DmrC-p65 plasmid (Addgene, #104564), and 500 ng of gRNA plasmids (Addgene, #65777) targeting the *AR* and *IRX4* promoter regions (Supplementary Data 1) using 20 ul strip with EN-150 program on a Lonza 4-D Nucleofector X Unit with the SF Cell Line Kit (Lonza). Cells were plated into 24 well plates and complete media containing 500 μM A/C heterodimerizer (Takara Clontech) was changed 24 h after transfection. Cells were harvested 36 h post transfection for RNA isolation and gene expression analysis.

### Gene expression analysis by qRT-PCR

For qRT-PCR 500 ng total RNA (Macherey- Nagel) was reverse transcribed (High Capacity Reverse transcription kit, LifeTechnologies) and cDNA was diluted (20x). SYBR Green assay was performed on Light Cycler 480 instrument (2x Probe Master Mix, Roche). All primer sequences are listed in Supplementary Data 1. Relative gene expression was calculated based on the ddCT method^52^. Each sample was measured by two biological and technical replicates. GAPDH1 gene was used as housekeeping genes to normalize the samples.

### Cell proliferation assays

Cell viability was quantified by measuring cellular ATP content using Presto Blue Viability assay (Thermo Fisher Scientific) according to the manufacturer’s instructions. All experiments were performed in triplicate in 96-well plates. Fluorescence signal at 560/590 nm was detected by Synergy2 plate reader (BioTek) using Gen5 (3.6.19.) software.

### Competitive cell growth assay

Flow sorting based competitive cell growth assay was performed as previously described^53^. LNCaP cells and LNCaP GFP stably expressing cells were transduced with *IRX4* ORF and *IRX4* targeting constructions followed by selection with blasticidin or puromycin, respectively. Cells were mixed in a 1:1 ratio and plated in a 12-well plate, to ensure that differences were not due to the GFP reporter activity. Cells were passaged every 3 days and relative ratios of cells were determined at indicated time points using FACS analysis. Averages of three-three replicates were plotted of each time point and Student *t*-test were performed.

### RNA-Seq analysis

Lenti viral *IRX4* over expression (OE) and knock-down (KD) was performed compared to vector control (VC) samples in LNCaP cell lines. For *IRX4* over expression MGC Human *IRX4* Sequence-Verified cDNA (CloneId:9020494) (Dharmacon, MHS6278-213243544) was cloned into pLVX-M-puro (Addgene, #125839) BamHI/EcoRI site. For suppression of *IRX4* level, IRX4#sh1 (Supplementary Data 6) was selected from Genetic Perturbation Platform (https://portals.broadinstitute.org/gpp/public/gene/search) cloned into pLKO.1 TRC cloning vector (Addgene, #10878). Puromycin selection (2 µg/μl) was performed for 10 days. For RNA-Seq analysis total RNA was extracted (Qiagen) from biological duplicates. Library preparation and RNA sequencing was performed by Novogene, Inc., using 200 ng high-quality input total RNA per sample. Differential expression analysis was performed using the DESeq2 R package. The resulting P-values were adjusted using Benjamini and Hochberg’s approach for controlling the False Discovery Rate (FDR). Genes with an adjusted *P*-value < 0.05 and log_2_FC > |1| found by DESeq2 were assigned as differentially expressed and listed in Supplementary Data 2a–e.

### Reporting summary

Further information on research design is available in the Nature Portfolio Reporting Summary linked to this article.

### Supplementary information

Supplementary Information

Description of Additional Supplementary Files

Supplementary Data 1-6

Reporting Summary

## Data Availability

Data sets generated in this study have been deposited in the Gene Expression Omnibus (GEO) database under accession code GSE231751 of super series including RNA-seq (GSE231747), ChIP-seq (GSE231747) and ATAC-seq (GSE231750) data. Sequencing reads are aligned to the human genome build hg19. Further information and requests for resources and reagents should be directed to and will be fulfilled by the lead contact, Matthew Freedman (matthew_freedman@dfci.harvard.edu).
