# Author Correction: A biallelic multiple nucleotide length polymorphism explains functional causality at 5p15.33 prostate cancer risk locus

**DOI:** 10.1038/s41467-023-42515-9

**Published:** 2023-10-19

**Authors:** Sandor Spisak, Viktoria Tisza, Pier Vitale Nuzzo, Ji-Heui Seo, Balint Pataki, Dezso Ribli, Zsofia Sztupinszki, Connor Bell, Mersedeh Rohanizadegan, David R. Stillman, Sarah Abou Alaiwi, Alan H. Bartels, Marton Papp, Anamay Shetty, Forough Abbasi, Xianzhi Lin, Kate Lawrenson, Simon A. Gayther, Mark Pomerantz, Sylvan Baca, Norbert Solymosi, Istvan Csabai, Zoltan Szallasi, Alexander Gusev, Matthew L. Freedman

**Affiliations:** 1 Department of Medical Oncology, Dana-Farber Cancer Institute, Boston, MA 02215 USA; 2 Center for Functional Cancer Epigenetics, Dana-Farber Cancer Institute, Boston, MA 02215 USA; 3 Computational Health Informatics Program (CHIP) Boston Children’s Hospital Harvard Medical School, Boston, MA 02215 USA; 4 Institute of Enzymology, Research Centre for Natural Sciences, Budapest, 1117 Hungary; 5 Department of Internal Medicine, School of Medicine, University of Genoa, Genoa, Lgo R. Benzi 10, 16132 Italy; 6 Department of Physics of Complex Systems, ELTE Eötvös Loránd University, Pázmány P. s. 1A, Budapest, 1117 Hungary; 7 Centre for Bioinformatics, University of Veterinary Medicine, Istvan str. 2, Budapest, 1078 Hungary; 8 Division of Genetics, Brigham & Women’s Hospital, Boston, MA USA; 9 Women’s Cancer Program, Samuel Oschin Comprehensive Cancer Institute, Cedars-Sinai Medical Center, Los Angeles, CA 90048 USA; 10 Division of Gynecologic Oncology, Department of Obstetrics and Gynecology, Cedars-Sinai Medical Center, Los Angeles, CA 90048 USA; 11 Center for Bioinformatics and Functional Genomics, Department of Biomedical Science, Cedars-Sinai Medical Center, Los Angeles, CA 90048 USA; 12 The Eli and Edythe L. Broad Institute, Cambridge, MA 02142 USA; 13 Department of Bioinformatics, Forensic and Insurance Medicine Semmelweis University, Budapest, Hungary; 14 Danish Cancer Society Research Center, Strandboulevarden 49, 2100 Copenhagen, Denmark; 15 National Korányi Institute of Pulmonology, Budapest, 1112 Hungary

**Keywords:** Cancer epigenetics, Cancer genomics, Prostate cancer

**Correction to**: *Nature Communications* 10.1038/s41467-023-40616-z, published online 23 Aug 2023

In this article, the author’s name Alan H. Bartels was incorrectly written as Alan B. Bartels. The original article has been corrected.

