# Supplementary material for: A biallelic multiple nucleotide length polymorphism explains functional causality at 5p15.33 prostate cancer risk locus
Source: Nat Commun. 2023 Aug 23;14:5118. doi: 10.1038/s41467-023-40616-z (PMC10447552; doi:10.1038/s41467-023-40616-z)
Supplement: Supplementary file 1 — Supplementary Information [file 41467_2023_40616_MOESM1_ESM.pdf]

# Supplementary Information

for

# A biallelic multiple nucleotide length polymorphism explains functional causality at 5p15.33 prostate cancer risk locus

Spisak et. al.

## Table of Content

Supplementary Figures……………………………………..………………………2

Supplementary Methods……………………………..……………………………32

Supplementary Methods References...……………………………..…………...…40

Supplementary Methods Figures……………………..…..…………………………41

# Supplementary Figures

**Supplementary Figure S1. | Variant analysis at MNLP position.**

**a,** The MNLP has a 21 bp S and 47 bp L allele. However, the S allele is not a simple deletion variation of the L allele, what reflects the complexity of this biallelic variant.

**b,** The region containing the MNLP position proved challenging to annotate for polymorphisms due to the complexity identified by the dbSNP Build 151 (2017) database, which documented 44 distinct variations consisting of 19 MNLPs/INDELs and 25 SNPs.

**c,** Genotyping the MNLP by Sanger-sequencing in four different PCa cell lines. The MNLP region was amplified (o458/o459)(**Supplementary Data S6**), and the PCR products were separated on agarose gel. Due to the allelic size differences (21 bp vs. 47 bp), amplicon size shifts (163 bp and 189 bp) were observed, correspond to the S and L alleles, respectively. PCR fragments were isolated from the agarose gel and subjected for Sanger-sequencing. The 163 bp fragments observed in LNCaP and 22Rv1 cell lines, consistently carried the S allele, while the 189 bp amplicon (observed VCaP, 22Rv1 and PC-3 cell lines) exclusively carried the L allele, supporting the existence of the biallelic MNLP.

**d**, PCa cell line MNLP genotypes. LNCaP is a tetraploid cell line, homozygous for the S (4) allele. VCaP is a triploid cell line, homozygous for the L (3) allele. 22Rv1 heterozygous (L/S) and PC-3 is homozygous (L/L) diploid cell lines.

**e,** Read percentage of L and S MNLP variant by deep amplicon sequencing of pooled germline DNA samples (n=54).

**f**, Number of samples with indicated genotype of L and S MNLP variant by deep amplicon sequencing of pooled germline DNA samples (n=56) as well as allelic frequency.

a

## Biallelic MNLP

**21 bp short (S) allele (rs745614767)**

tccgt-------------------CAGGACTTGCAAGCTCATGTG-------gcgtc

tccgtGCCGCACTGTTGATCTTGG**CAGG**CC**CTT**CGG**G**AC**CAA**CAGGGACAGTgcgtc

**47 bp long (L) allele (rs386684493)**

* rs745614767= short allele

** rs386684493 = long allele

b

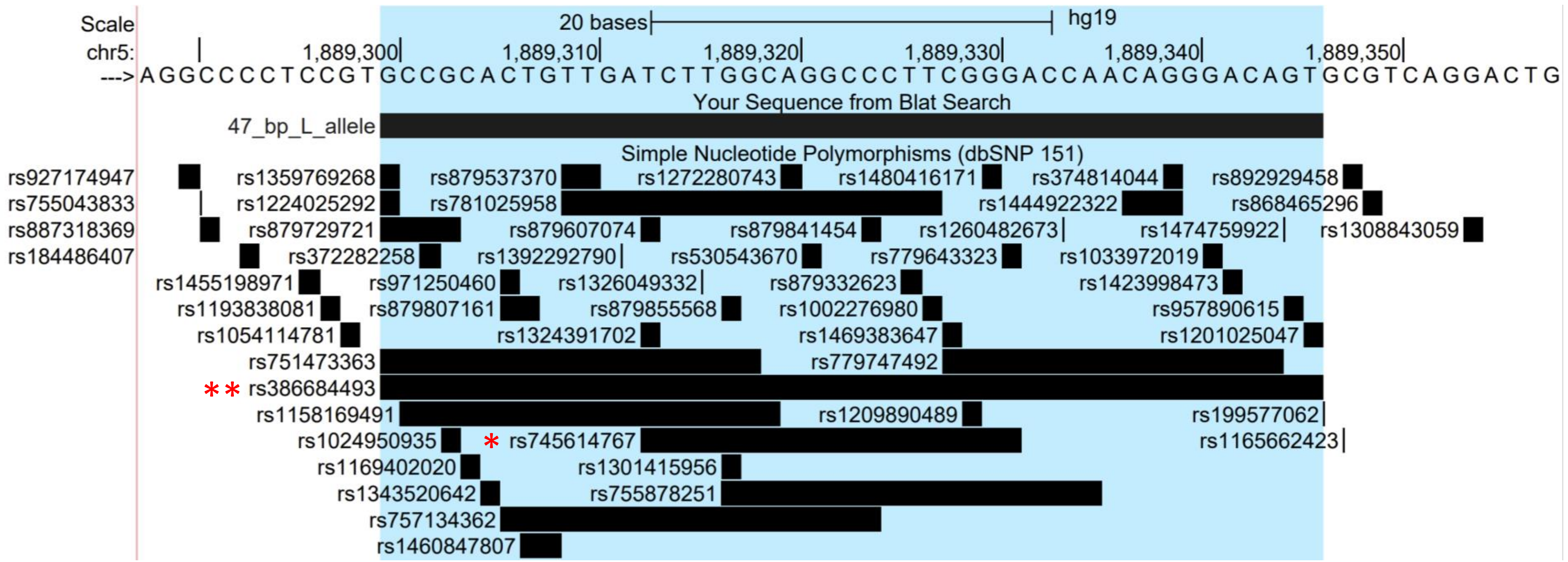

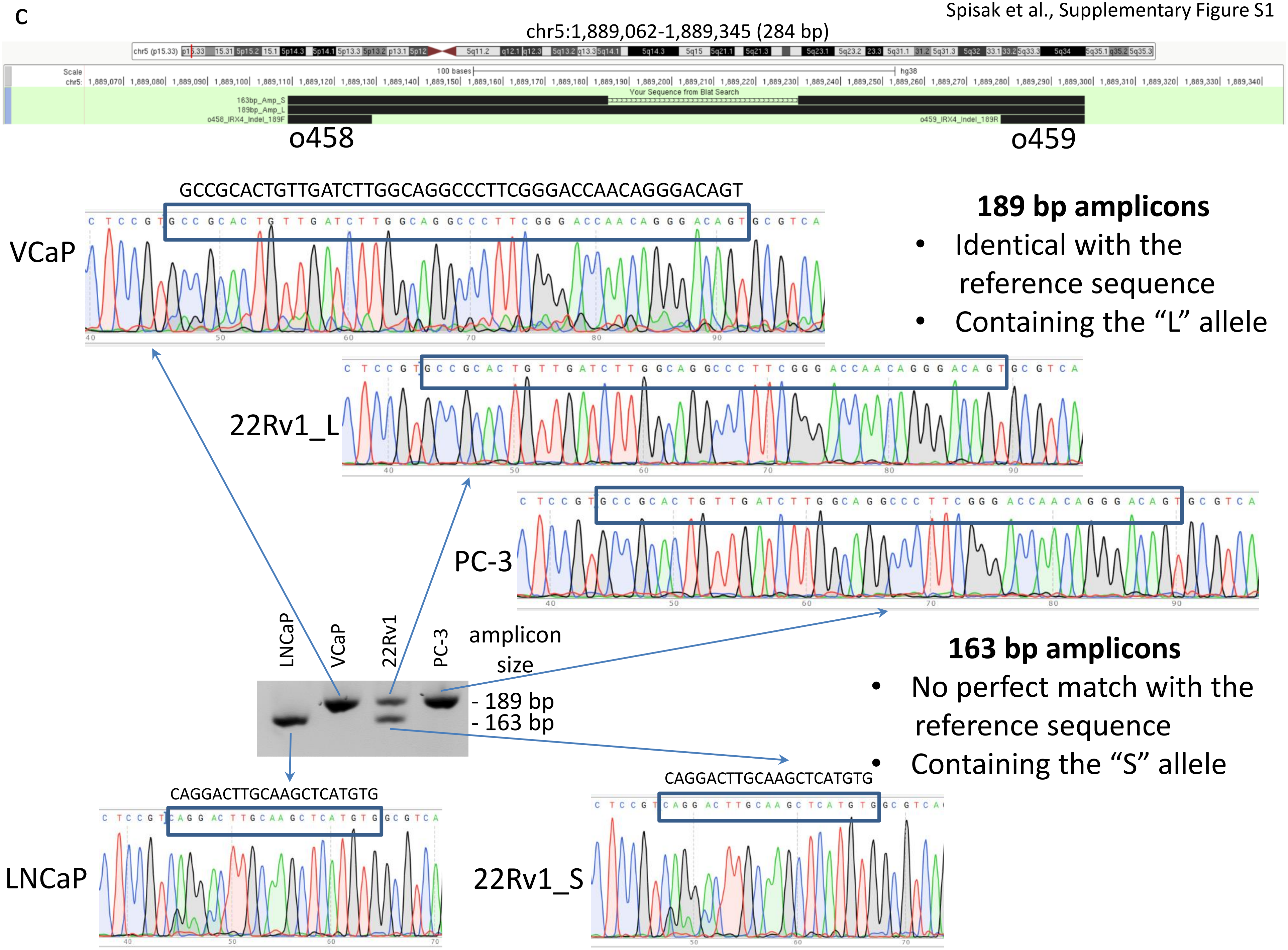
c
chr5:1,889,062-1,889,345 (284 bp)
o458
o459
GCCGCACTGTTGATCTTGGCAGGCCCTTCGGGACCAACAGGGACAGT
VCaP
22Rv1_L
PC-3
189 bp amplicons
• Identical with the reference sequence
• Containing the "L" allele
LNCaP
VCaP
22Rv1
PC-3
amplicon size
- 189 bp
- 163 bp
163 bp amplicons
• No perfect match with the reference sequence
• Containing the "S" allele
CAGGACTTGCAAGCTCATGTG
LNCaP
CAGGACTTGCAAGCTCATGTG
22Rv1_S

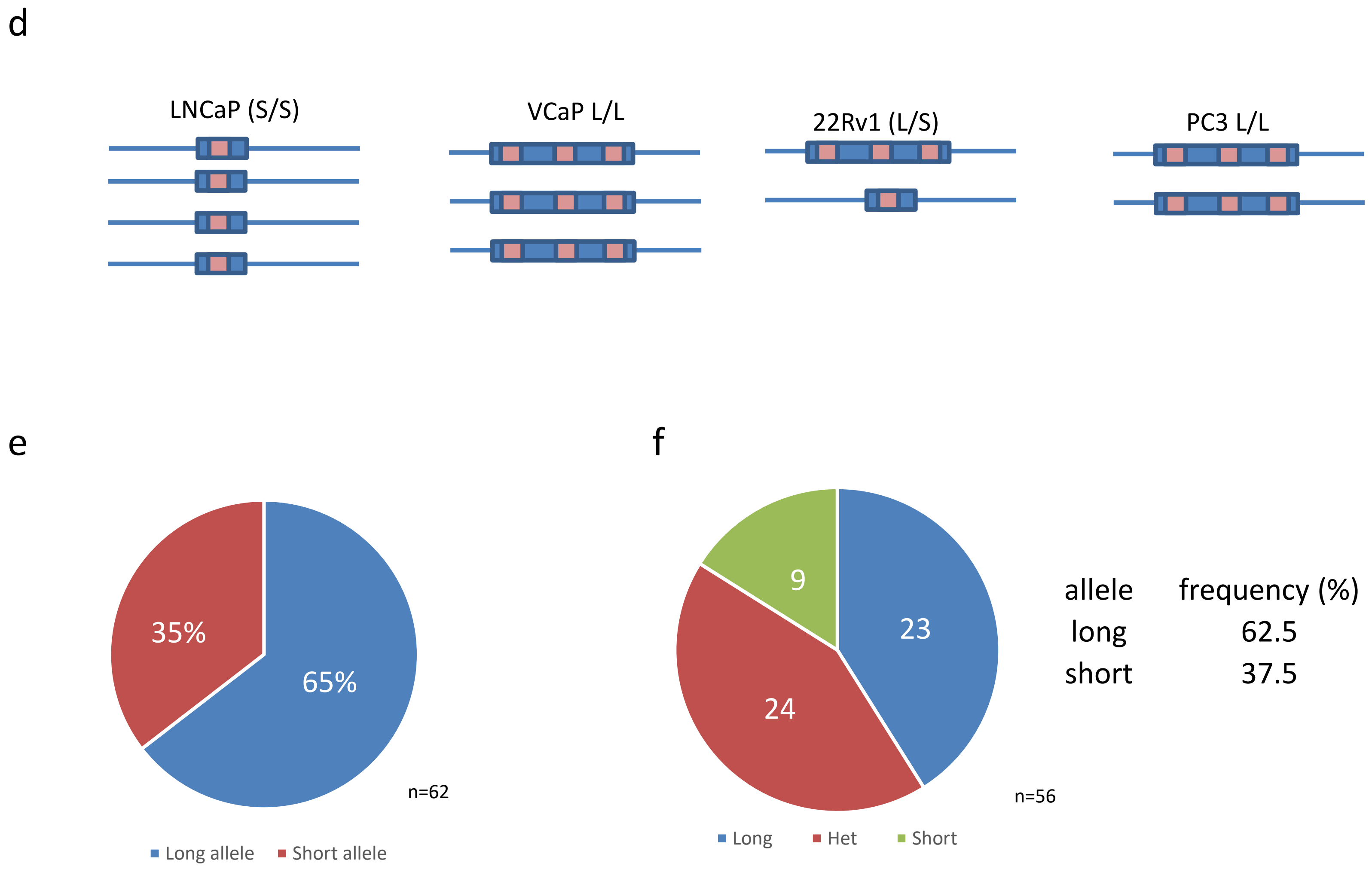
d
LNCaP (S/S)
VCaP L/L
22Rv1 (L/S)
PC3 L/L
e
35%
65%
n=62
Long allele
Short allele
f
9
23
24
n=56
Long
Het
Short
allele frequency (%)
long 62.5
short 37.5

**Supplementary Figure S2. | Genotype correlation, eQTL and chromQTL analysis.**

**a,** Genotype correlation between the MNLP and rs12653946 in all 1310 germline TCGA samples.

**b,** Genotype correlation between the MNLP and rs12653946 in 121 PRAD TCGA samples.

**c,** Genetic correlation analysis revealed linkage between L allele at the MNLP position and C allele at the rs12653946 SNP position. The MNLP S allele linked to the T risk allele at the rs12653946 position.

**d,** Genotype vs. gene expression correlation. Average *IRX4* level for each recombinant group were plotted into a genotype matrix. The genotype matrix was created by plotting the rs12653946 top SNP genotypes (horizontal axis T/T, T/C and C/C) against the MNLP genotypes (S/S, S/L and L/L). Given the significant smaller size (n=121) of the PRAD cohort compared to the 1,310 TCGA samples, the two extreme genotypes (T/T L/L and C/C S/S) are missing from this cohort. There is a clear trend on the gene expression, the T and S allele associates with the low expression, and the presence of the C and L alleles associate with higher *IRX4* levels.

**e,** All observed recombinant genotypes (left panel MNLP genotypes, right panel rs12653946 top SNP genotypes) were taken and average *IRX4* level for each group were plotted against them. Recombinant genotypes show, that the MNLP genotypes have stronger effect on the *IRX4* level than genotypes of the rs12653946 top SNP.

**f,** H3K27ac signals in 27 human tissue PCa tumor samples. Horizontal axis show genetic positions, with the MNLP position marked by vertical stripe. On the y axis clinical samples are listed, within each row normalized H3K27ac signal intensities are plotted. Samples are grouped by genotypes, each MNLP genotype is color coded; L/L=green (n=6), L/S=orange (n=18), S/S=light blue (n=3). The presence of the L allele indicates higher H3K27ac activity.

**g,** Summary plot and statistics for samples on panel f.

**h,** H3K27ac ChIP-seq data visualized (chr5:188900-1890000, hg19) in IGV browser using aligned bam files and coverage plots from a representative heterozygous (S/L) sample.

**i,** Reads from bam file (panel h) were realigned to S reference sequence (**Supplementary Data S1**)

**j,** Reads from bam file (panel h) were realigned to L reference sequence (**Supplementary Data S1).** This analysis confirmed L allele specific chromatin activity using H3K27ac ChIP-seq data.

Data source for panel f-j is listed in **Supplementary Data S5.**

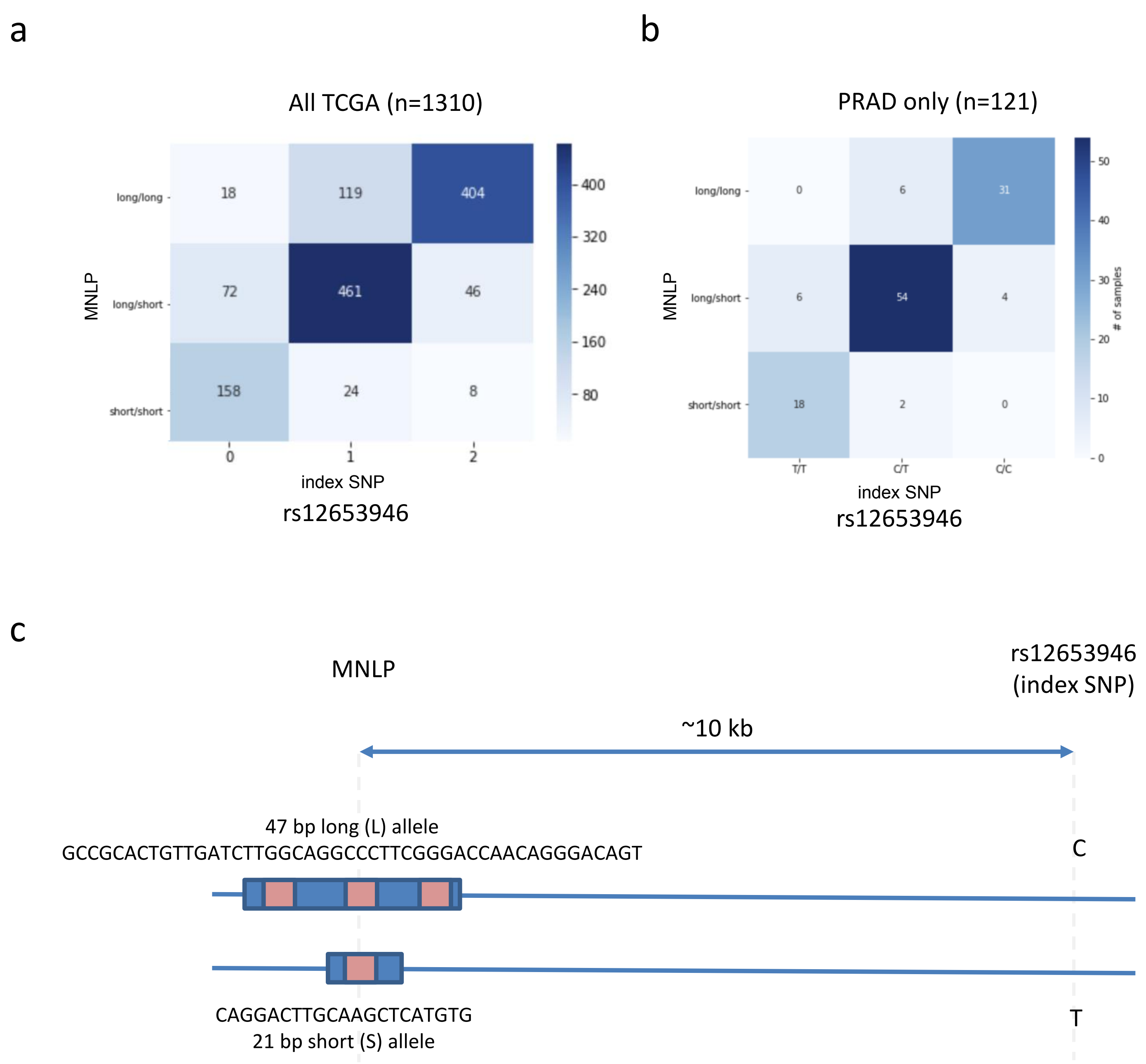
a
All TCGA (n=1310)
MNLP
long/long
long/short
short/short
18
119
404
72
461
46
158
24
8
400
320
240
160
80
0
1
2
index SNP
rs12653946
b
PRAD only (n=121)
MNLP
long/long
long/short
short/short
0
6
31
6
54
4
18
2
0
50
40
30
20
10
0
# of samples
T/T
C/T
C/C
index SNP
rs12653946
c
MNLP
rs12653946
(index SNP)
~10 kb
47 bp long (L) allele
GCCGCACTGTTGATCTTGGCAGGCCCTTCGGGACCAACAGGGACAGT
C
CAGGACTTGCAAGCTCATGTG
21 bp short (S) allele
T

Spisak et al., Supplementary Figure S2

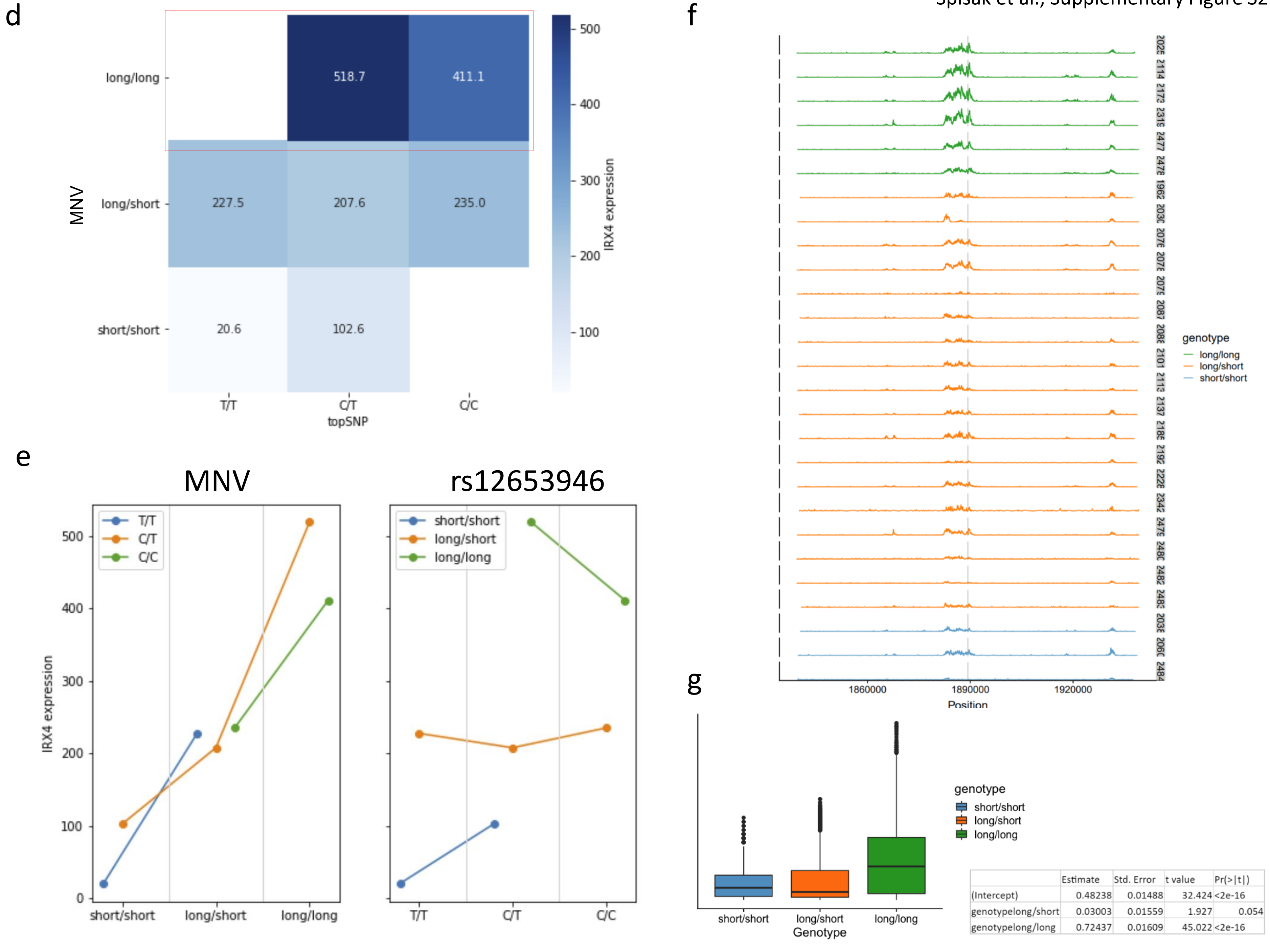


| | Estimate | Std. Error | t value | Pr(>\|t\|) |
|---|---|---|---|---|
| (Intercept) | 0.48238 | 0.01488 | 32.424 | <2e-16 |
| genotypelong/short | 0.03003 | 0.01559 | 1.927 | 0.054 |
| genotypelong/long | 0.72437 | 0.01609 | 45.022 | <2e-16 |

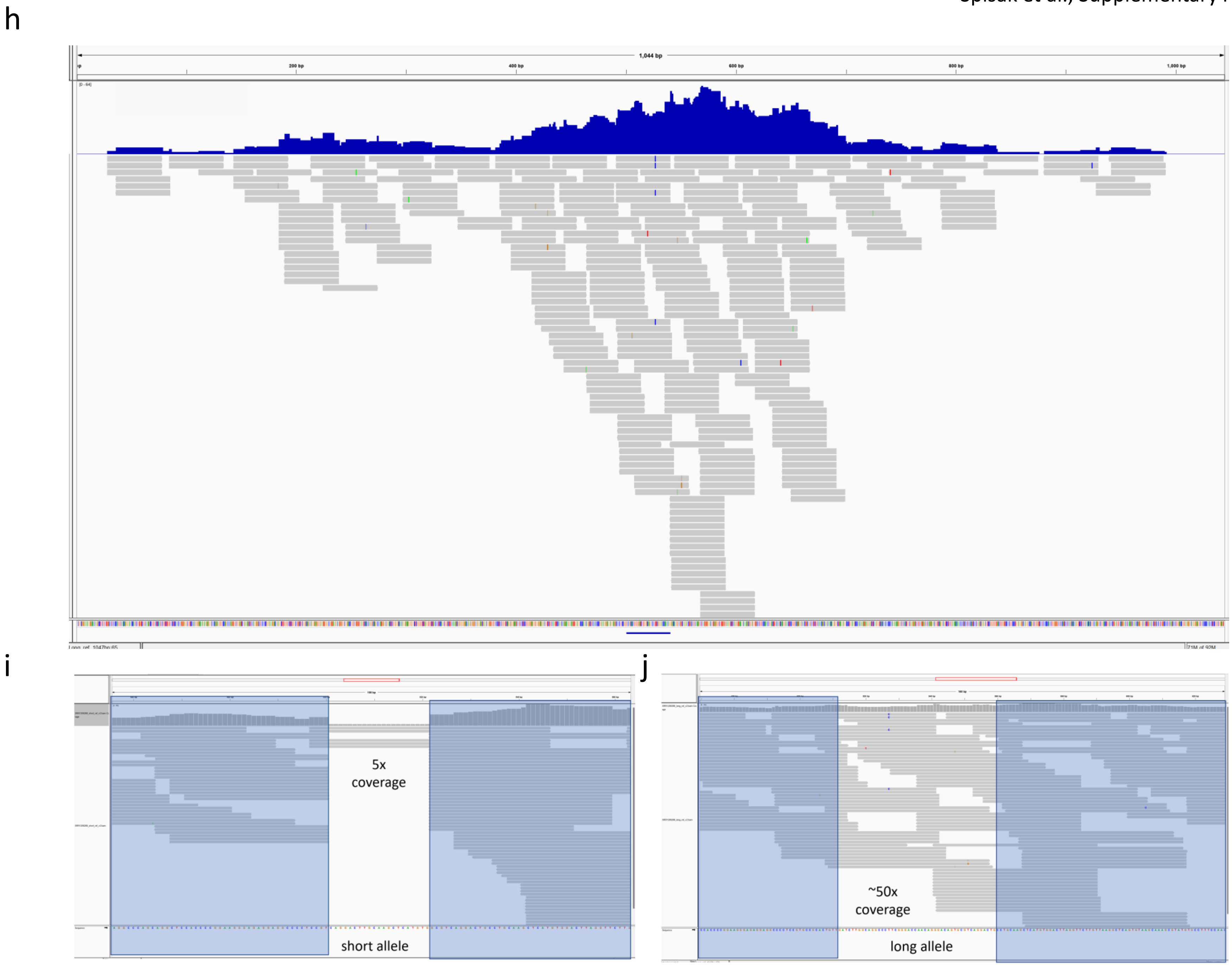
h
1,044 bp
200 bp
400 bp
600 bp
800 bp
1,000 bp
i
5x
coverage
short allele
j
~50x
coverage
long allele

**Supplementary Figure S3. | CRISPRi experimental details.**

**a,** Normalized *IRX4* levels after CRISPR inhibition. Targeting the correlated SNP region with different guide RNAs resulted no alteration in *IRX4* level in both cell lines compared to the non-target control (CTRL1). Only the L allele targeting guides (L1, L2 and L3) showed *IRX4* suppression in VCaP cells, which indicates that the L allele has regulatory potential to influence *IRX4* expression. S allele targeting gRNAs (S1, S2 and S3) showed no effect neither in LNCaP nor in VCaP cell lines indicating the specificity of the experiment and confirming the L allele related higher epigenetic activity. Experiments were independently repeated three times (n=3), and the average values are shown on the bars, while individual values are represented by dots. Error bars indicate the standard deviation of the three biological replicates. A two-sided t-test was used to calculate statistical significance.

**b**, Allele specific MNLP targeting gRNAs. Three S allele specific gRNA (S1, S2 and S3) and three L allele specific gRNAs (L1, L2 and L3) were used compared to non-target control (CTRL1).

**c,** Long and short allele specific gRNA localizations to specifically target MNLP alleles. gRNA sequences are listed in Supplementary Data S6.

**d**, genomic position of the *IRX4* region and the targeted variants.

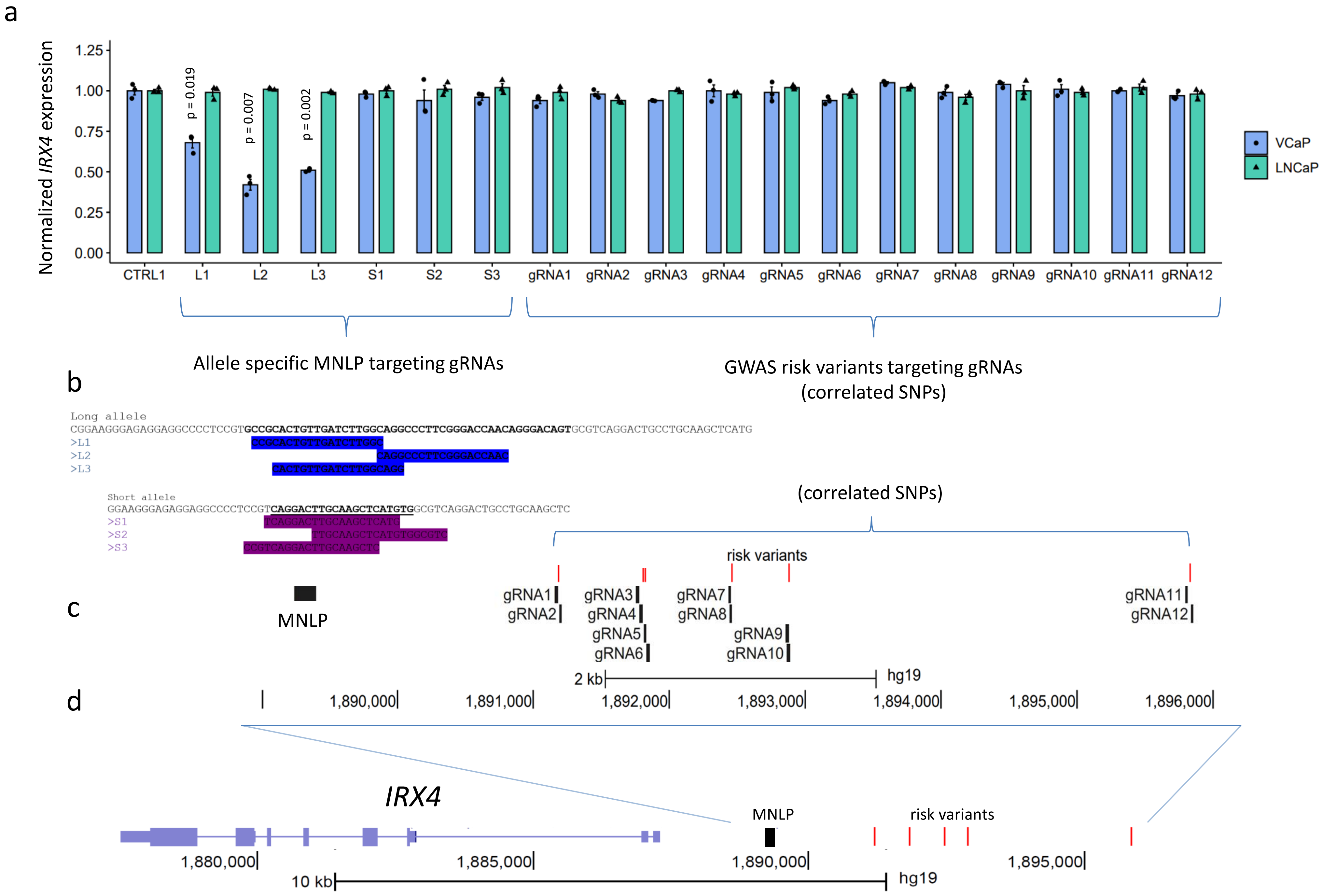
a
Normalized IRX4 expression
1.25
1.00
0.75
0.50
0.25
0.00
p = 0.019
p = 0.007
p = 0.002
CTRL1
L1
L2
L3
S1
S2
S3
gRNA1
gRNA2
gRNA3
gRNA4
gRNA5
gRNA6
gRNA7
gRNA8
gRNA9
gRNA10
gRNA11
gRNA12
VCaP
LNCaP
Allele specific MNLP targeting gRNAs
GWAS risk variants targeting gRNAs
(correlated SNPs)
b
Long allele
CGGAAGGGAGAGGAGGCCCCTCCGTGCCGCACTGTTGATCTTGGCAGGCCCTTCGGGACCAACAGGGACAGTGCGTCAGGACTGCCTGCAAGCTCATG
>L1 CCGCACTGTTGATCTTGGC
>L2 CAGGCCCTTCGGGACCAAC
>L3 CACTGTTGATCTTGGCAGG
Short allele
GGAAGGGAGAGGAGGCCCCTCCGTCAGGACTTGCAAGCTCATGTGGCGTCAGGACTGCCTGCAAGCTC
>S1 TCAGGACTTGCAAGCTCATG
>S2 TTGCAAGCTCATGTGGCGTC
>S3 CCGTCAGGACTTGCAAGCTC
(correlated SNPs)
risk variants
c
MNLP
gRNA1
gRNA2
gRNA3
gRNA4
gRNA5
gRNA6
gRNA7
gRNA8
gRNA9
gRNA10
gRNA11
gRNA12
2 kb
hg19
d
1,890,000
1,891,000
1,892,000
1,893,000
1,894,000
1,895,000
1,896,000
IRX4
MNLP
risk variants
1,880,000
1,885,000
1,890,000
1,895,000
10 kb
hg19

**Supplementary Figure S4. | Schematic figure of the L allele knock-in in LNCaP cells using the CAUSEL pipeline.**

**a**, Chromatogram from Sanger sequencing of parental LNCaP cells confirm that LNCaP is carrying the S allele in homozygous format.

**b**, LNCaP cells carrying the S (4) allele were targeted by allele specific gRNA to cleave the S allele and introduce the L allele by HDR mediated knock-in using ssODN template, carrying the L allele sequence. After genome editing, single cells were regenerated and subjected for genotyping. The L allele carriers were regenerated and subjected for transposase accessibility (ATAC-seq), H3K27ac ChIP-seq and gene expression analysis to determine *IRX4* levels (**Figure 4**). Underlined 21 bp region in the L allele shows the similarity between the L and S alleles. Bold letters are highlighting the identical nucleotides at the corresponding position. Light lower-case grey letters indicate 5 bp left and 5 bp right genomic border sequences on both S and L alleles.

a

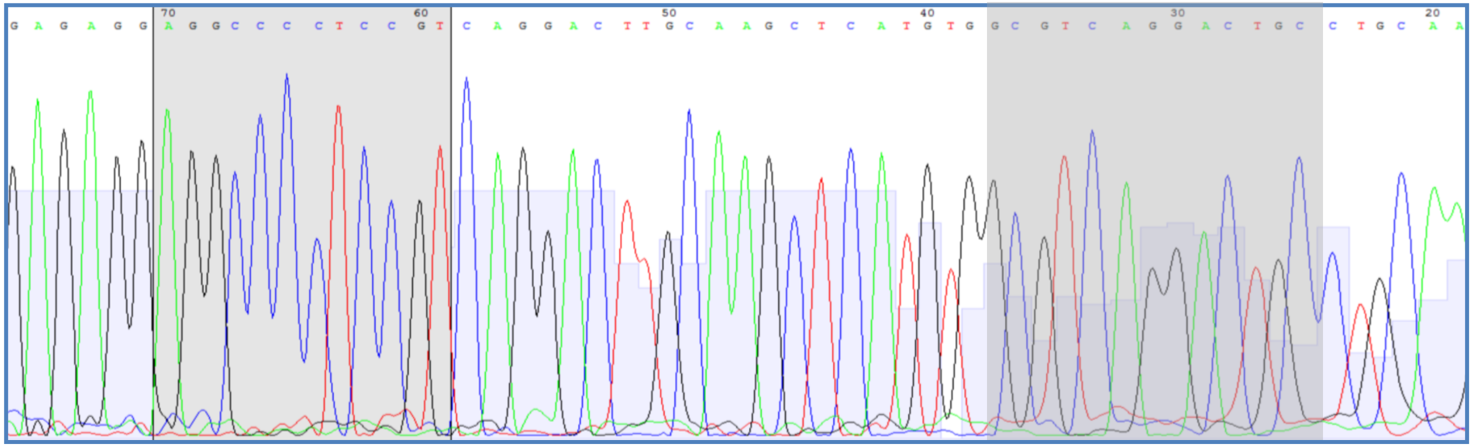

b

LNCaP

tccgtCAGGACTTGCAAGCTCATGTGgcgtc

21 bp short (S) allele

S allele

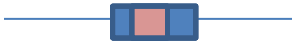

CAUSEL pipeline
(Spisak et. al., 2015 NatMed)

Genome editing (allelic knock in)
Single cell cloning
Genotyping
Functional assays

isogenic clones

tccgtGCCGCACTGTTGATCTTGG**CAGG**CCC**T**T**C**GG**G**AC**CA**ACA**G**GGACAGTgcgtc

47 bp long (L) allele

Knock-in 1
and
Knock-in 2

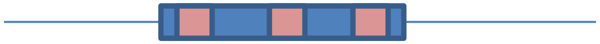

L allele

**Supplementary Figure S5. | TF binding and motif analysis.**

**a,** TF binding analysis using Cistrome db publicly available data set. X axis represents the overlapped peak ratio in your input interval. Y axis represents different factors. Dots in a y axis line means same factor. The MNLP has multiple TF binding, prostate relevant TFs are indicated by arrows. For box plots, error bars are the 95% confidence interval.

**b,** TF motif analysis of the L and S alleles. The S allele has ETV1 motif, the L allele has AR, ETV1 and NKX-3.1 motif predicted.

**c,** Genotype dependent TF binding and epigenetic activity. VCaP cells (L/L) has AR binding at the MNLP position which associates with H3K27ac signal. LNCaP cells have no AR binding at the MNLP position (S allele lack of AR binding motif) only ETV1 binds to the S allele, but there is no H3K27ac signal at the MNLP position. Data source is listed in **Supplementary Data S5.**

Spisak et al., Supplementary Figure S5

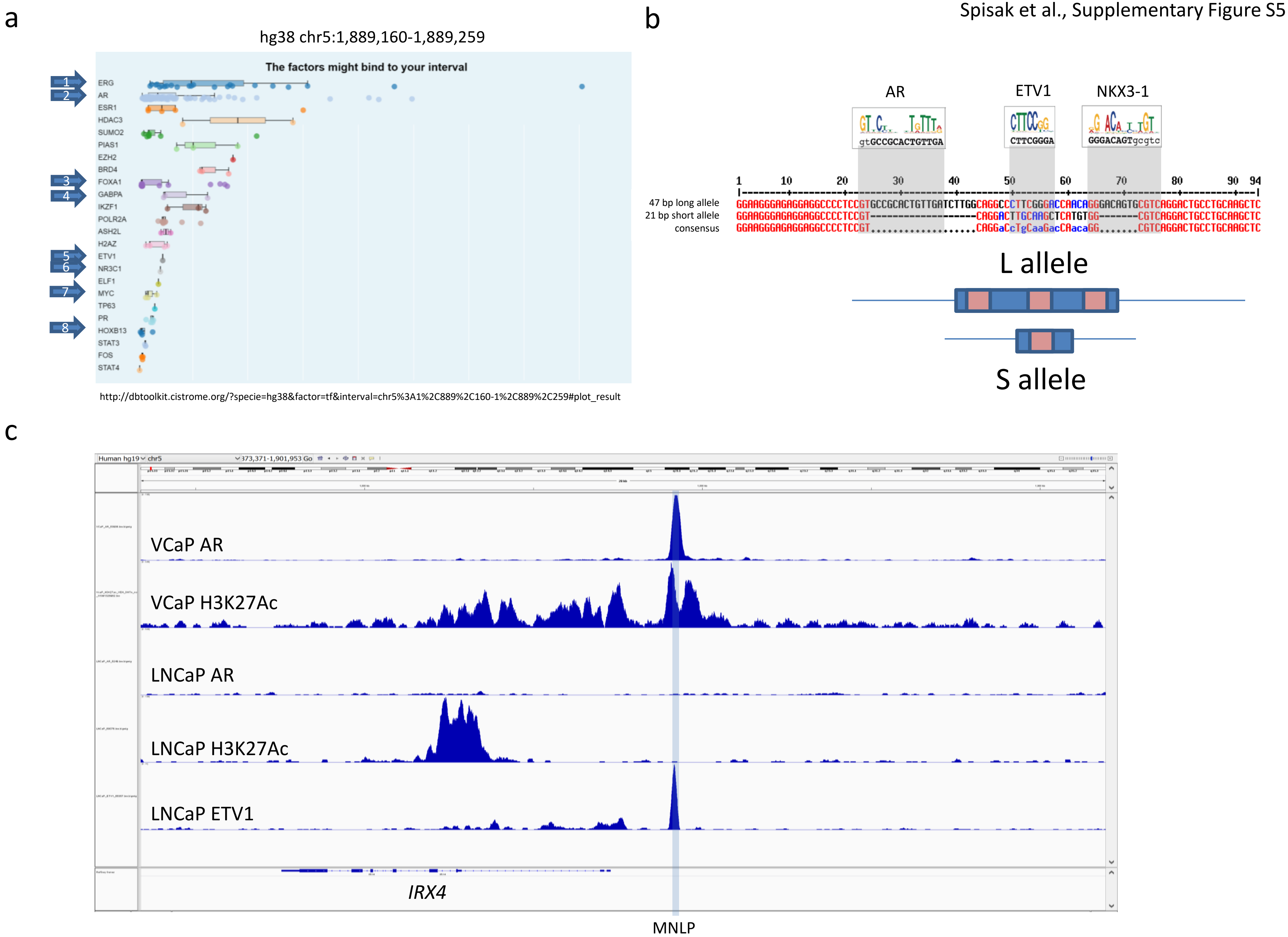

**Supplementary Figure S6. | Four prostate relevant TFs overexpression and KO have no effect on the *IRX4* expression.**

According to the motif and binding analysis four prostate relevant transcription factor (NKX3-1, ETV1, HOXB13, FOXA1) were overexpressed and knocked down in LNCaP cells, enhancer modified clones (Knock-in 1 and Knock-in 2) and VCaP cells followed by measuring the *IRX4* level. Manipulation none of these factors resulted significant *IRX4* level alteration. Each experiment was independently repeated three times (n=3), and the average values are shown on the bars, while individual values are represented by dots. Error bars indicate the standard deviation of the three biological replicates. A two-sided t-test was used to calculate statistical significance. *<0.05.

Spisak et al., Supplementary Figure S6

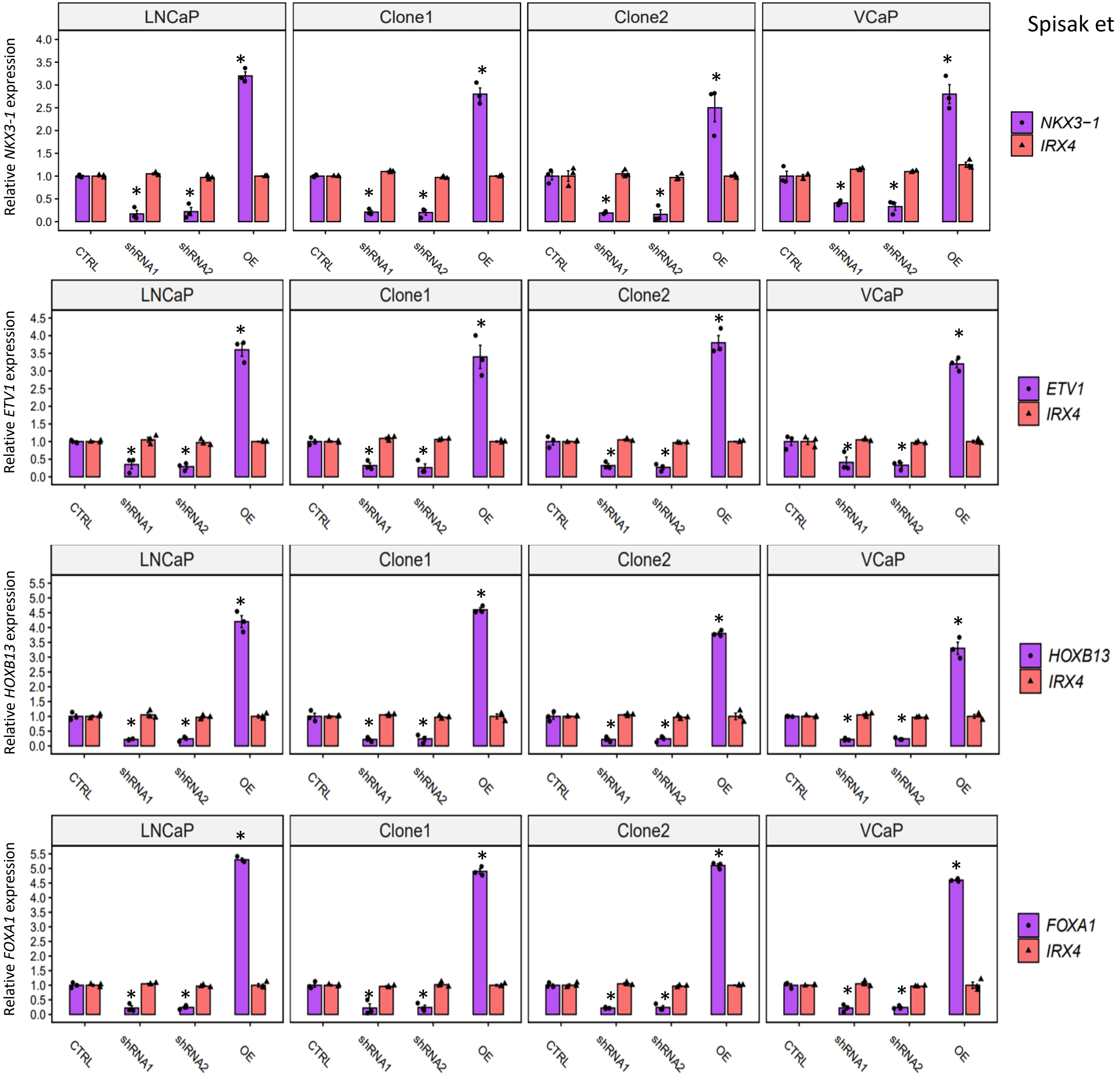


* p < 0.05

**Supplementary Figure S7. | Analyzing the role of AR as a potential *IRX4* regulator using publicly available data and Functional analysis of *IRX4*.**

Data source for both panel a and b are listed in **Supplementary Data S5.**

**a,** Individual datapoints of *IRX4* level from GEO (GSE135879) data set which indicates, that *IRX4* level has response of the induction or inhibition of the AR level in VCaP cells.

**b,** Overexpression of AR in AR negative PC3 cells resulted AR binding to the L allele at the MNLP position.

**c,** *IRX4* shows relatively tissue specific expression. Beside prostate it expressed in skin, esophagus, vagina, heart and salivary gland tissues at relative low level.

**d,** Cell growing assay demonstrate, that altering *IRX4* level have no effect on cell proliferation.

**e,** Competitive cell growth assay showed that there is no competitive advantage on cell growing of manipulation of *IRX4* level.

Dots represent the average of three biological replicates (panel b and c). Error bars indicating the standard deviation. A two-sided Student t-test was applied to calculate statistical significance.

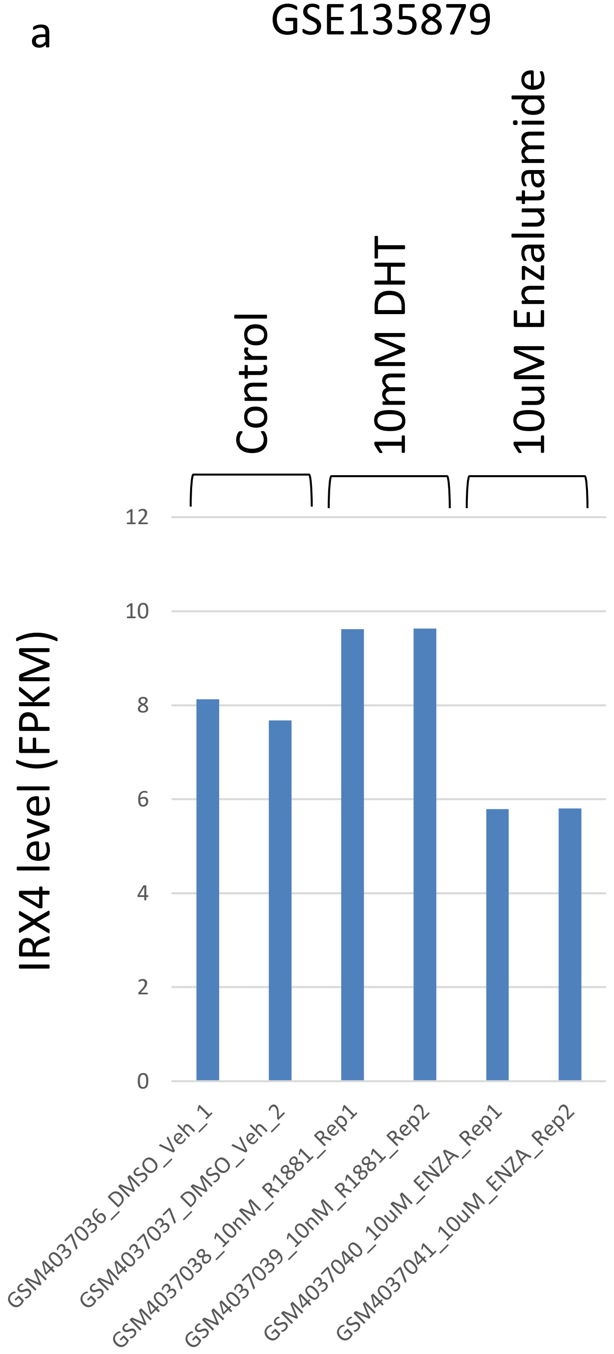
a
GSE135879
Control
10mM DHT
10uM Enzalutamide
IRX4 level (FPKM)
12
10
8
6
4
2
0
GSM4037036_DMSO_Veh_1
GSM4037037_DMSO_Veh_2
GSM4037038_10nM_R1881_Rep1
GSM4037039_10nM_R1881_Rep2
GSM4037040_10uM_ENZA_Rep1
GSM4037041_10uM_ENZA_Rep2

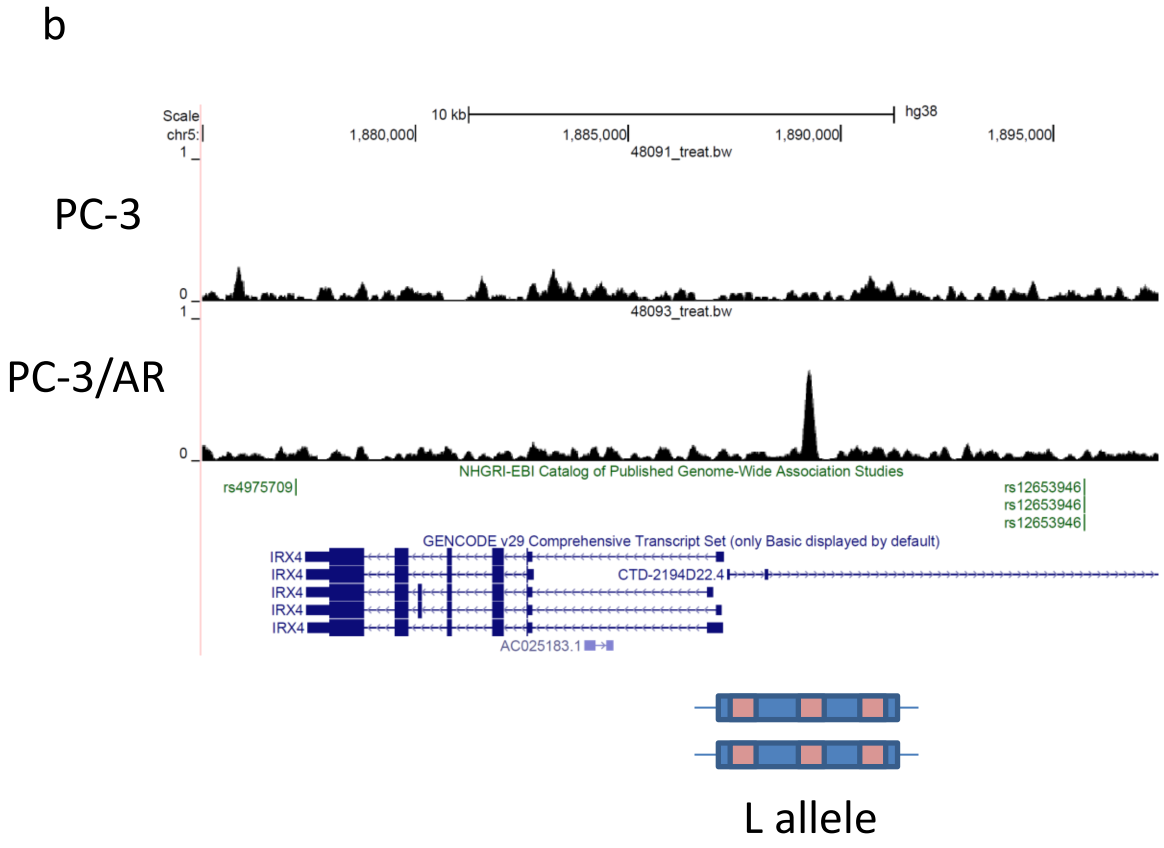
b
Scale
chr5:
10 kb
hg38
1,880,000
1,885,000
1,890,000
1,895,000
48091_treat.bw
PC-3
48093_treat.bw
PC-3/AR
NHGRI-EBI Catalog of Published Genome-Wide Association Studies
rs4975709
rs12653946
rs12653946
rs12653946
GENCODE v29 Comprehensive Transcript Set (only Basic displayed by default)
IRX4
IRX4
IRX4
IRX4
IRX4
CTD-2194D22.4
AC025183.1
L allele

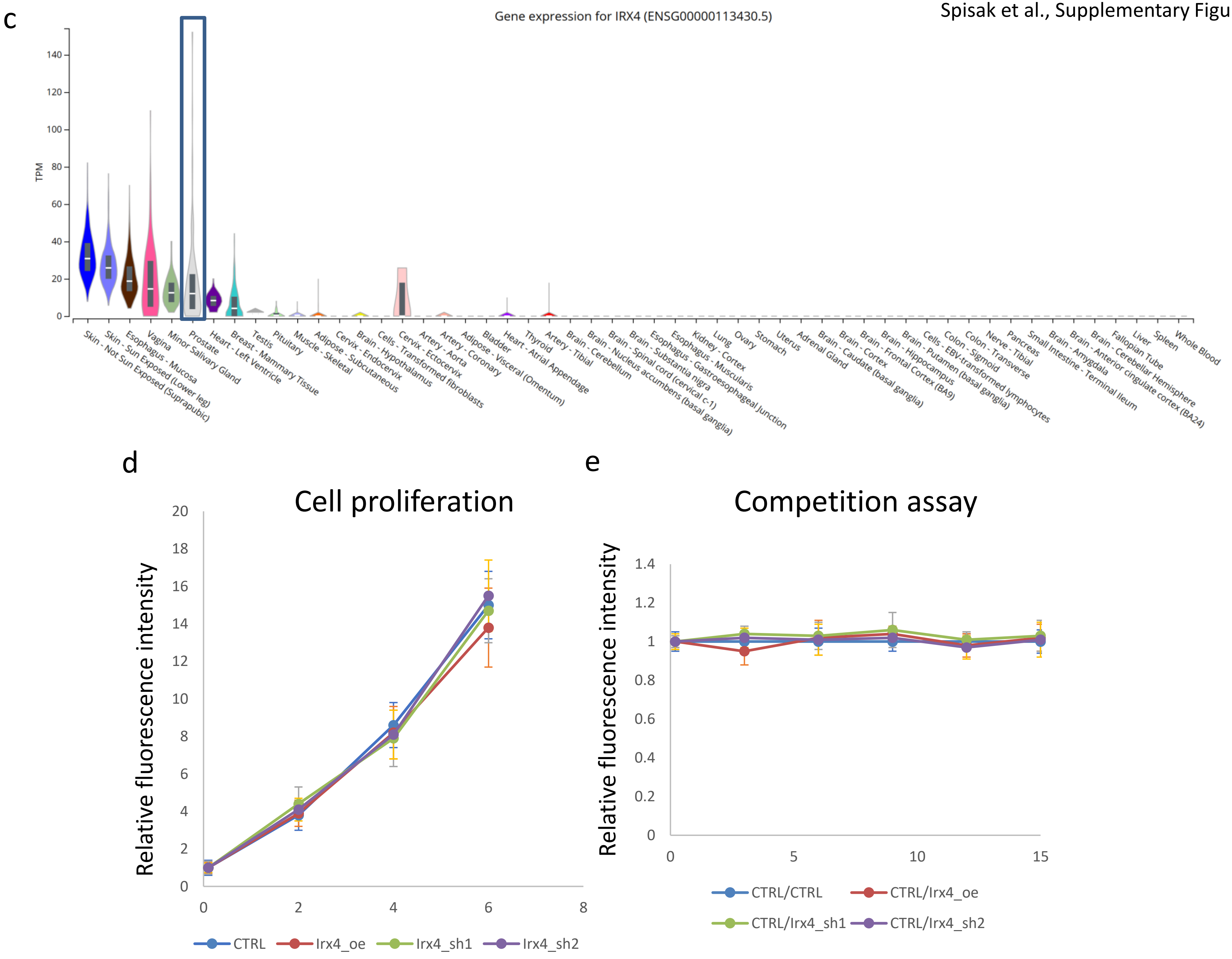
c
Gene expression for IRX4 (ENSG00000113430.5)
TPM
0
20
40
60
80
100
120
140
Skin - Not Sun Exposed (Suprapubic)
Skin - Sun Exposed (Lower leg)
Esophagus - Mucosa
Vagina
Minor Salivary Gland
Prostate
Heart - Left Ventricle
Breast - Mammary Tissue
Testis
Pituitary
Muscle - Skeletal
Adipose - Subcutaneous
Cervix - Endocervix
Brain - Hypothalamus
Cells - Transformed fibroblasts
Cervix - Ectocervix
Artery - Aorta
Artery - Coronary
Adipose - Visceral (Omentum)
Bladder
Heart - Atrial Appendage
Thyroid
Artery - Tibial
Brain - Cerebellum
Brain - Nucleus accumbens (basal ganglia)
Brain - Spinal cord (cervical c-1)
Brain - Substantia nigra
Esophagus - Gastroesophageal Junction
Esophagus - Muscularis
Kidney - Cortex
Lung
Ovary
Stomach
Uterus
Adrenal Gland
Brain - Caudate (basal ganglia)
Brain - Cortex
Brain - Frontal Cortex (BA9)
Brain - Hippocampus
Brain - Putamen (basal ganglia)
Cells - EBV-transformed lymphocytes
Colon - Sigmoid
Colon - Transverse
Nerve - Tibial
Pancreas
Small Intestine - Terminal Ileum
Brain - Amygdala
Brain - Anterior cingulate cortex (BA24)
Brain - Cerebellar Hemisphere
Fallopian Tube
Liver
Spleen
Whole Blood
d
Cell proliferation
Relative fluorescence intensity
0
2
4
6
8
10
12
14
16
18
20
0
2
4
6
8
CTRL
Irx4_oe
Irx4_sh1
Irx4_sh2
e
Competition assay
Relative fluorescence intensity
0
0.2
0.4
0.6
0.8
1
1.2
1.4
0
5
10
15
CTRL/CTRL
CTRL/Irx4_oe
CTRL/Irx4_sh1
CTRL/Irx4_sh2

**Supplementary Figure S8. | RNA-Seq analysis using LNCaP cell lines with manipulated *IRX4* levels.**

RNA-Seq analysis after manipulation the *IRX4* level in LNCaP cells. Volcano plots (**a-c**) and heatmaps (**d and e**) show the differentially expressed genes across three comparisons. Gene ontology analysis (**f and g**) was performed to demonstrate altered biological processes. Related processed data collected in Supplementary Data S2a-e.

Spisak et al., Supplementary Figure S8

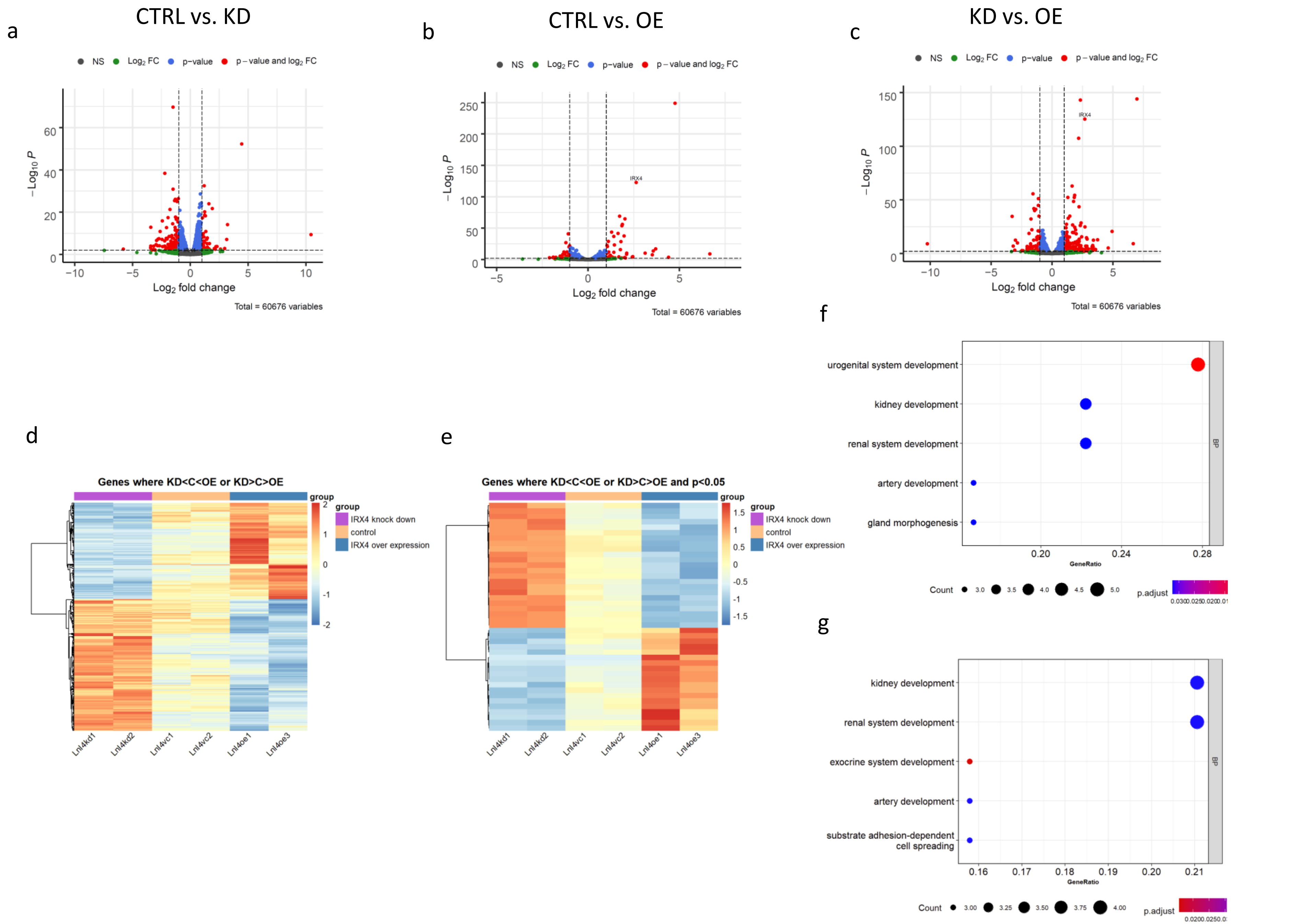

**Supplementary Figure S9. | Index SNPs and complex variant correlation analysis revealed two other complex variant candidates.**

In addition to the *IRX4* MNLP (**a**) two additional GWAS correlated complex variants were identified on chr2 and chr10 which has two allelic variants (**b** and **c**) and show high correlation with the predicted GWAS top SNP (**d, e** and **f**) and show epigenetic activity overlapping by Cistrome db (**g, h** and **i**).

**j,** Statistical summary of the different analysis. Columns: BEST.GWAS.Z: Z score for the most significant GWAS SNP, MODELCV.PV: P-value for the cross-validated predictive accuracy of the complex variant model, GWAS.Z: Z-score for the association of the predicted complex variant with GWAS risk, COLOC.PP3: Probability that the complex variant and GWAS risk are driven by different genetic mechanisms (estimated by COLOC), COLOC.PP4: Probability that the complex variant and the GWAS risk are driven by the same genetic mechanism (estimated by COLOC).

**k,** Overlapping analysis of MNLP14 revealed overlapping with 22 genetic variants in dbSNP151 (**Supplementary Data S3**).

**l**, Overlapping analysis of MNLP14 revealed overlapping with 11 genetic variants in dbSNP151 (**Supplementary Data S3**).

MNLP16 has a clear 25 bp insertion, however in case of MNLP3 and MNLP14 the short and long alleles have unique sequent components as well (**a-c**).

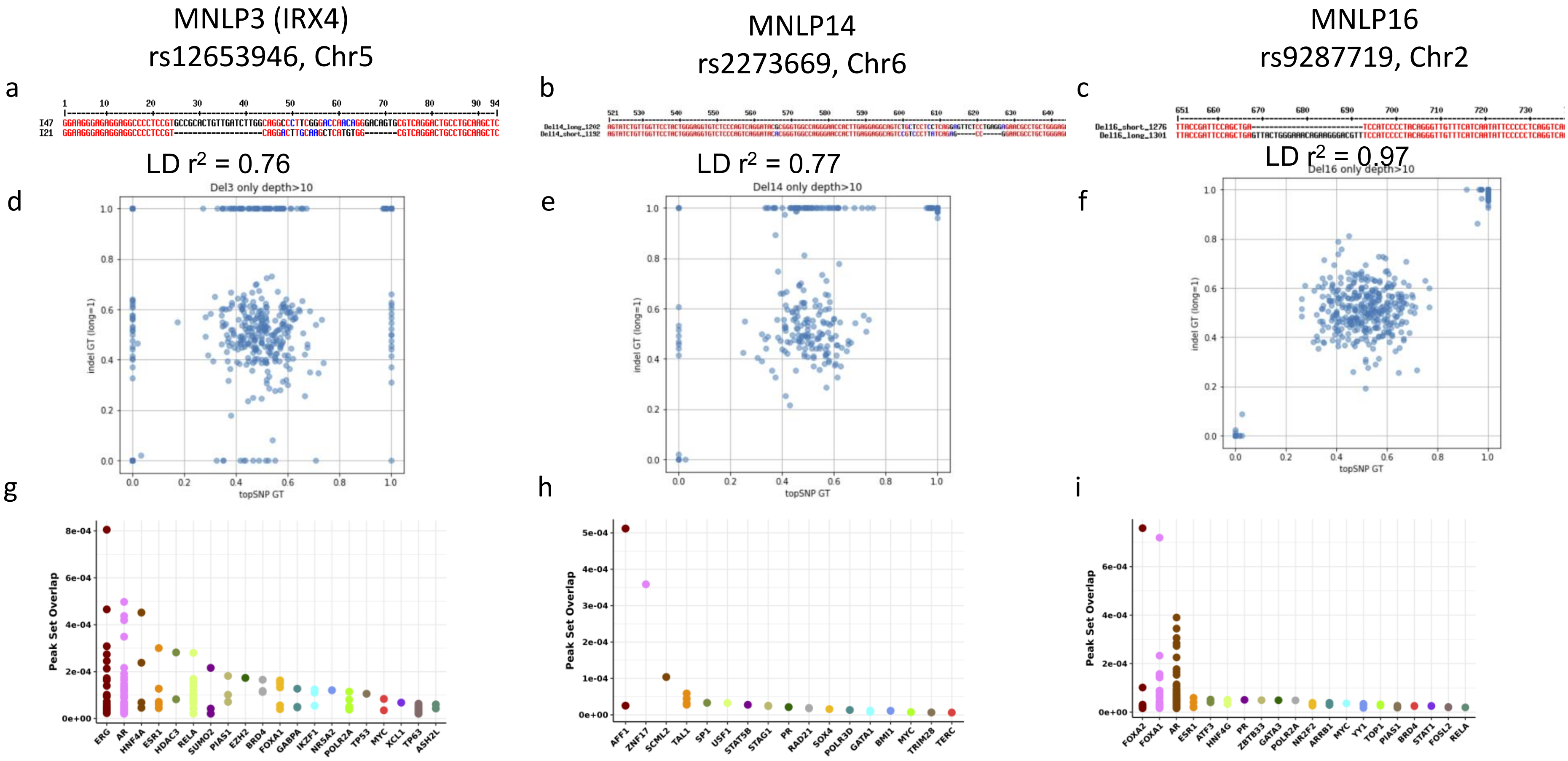


j

| Del ID | Chr | Head SNP | BEST.GWAS.Z | MODELCV.R2 | MODELCV.PV | TWAS.Z | TWAS.P | COLOC.PP3 | COLOC.PP4 |
|---|---|---|---|---|---|---|---|---|---|
| MNLP3(IRX4) | 5 | rs12653946 | 9.59 | 0.74 | 1.00E-176 | -9.93 | 3.00E-23 | 0 | 1 |
| MNLP14 | 6 | rs2273669 | 6.31 | 0.95 | 0.00E+00 | -5.8 | 6.00E-09 | 0.09 | 0.91 |
| MNLP16 | 2 | rs9287719 | -8.56 | 0.97 | 0.00E+00 | 8.39 | 5.00E-17 | 0.02 | 0.99 |

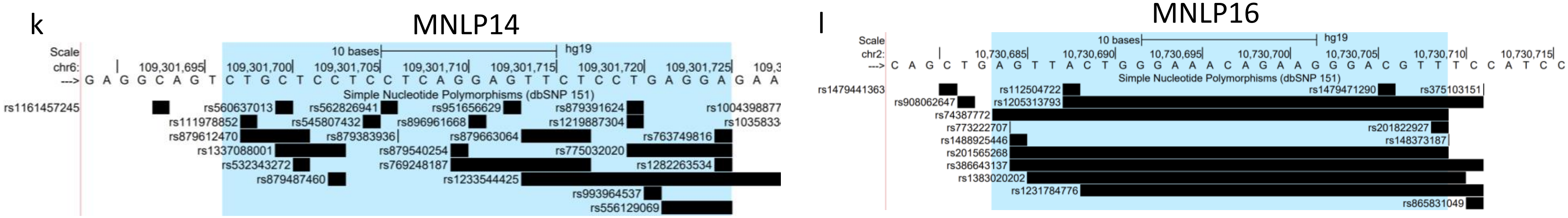

**Supplementary Figure S10. | Amplicon sequencing verification of the predicted MNLP/INDEL complex variant alleles.**

Most frequent read types from amplicon sequencing were aligned against the hg19 reference genome (BLAT) to verify complex variant genotypes and visualized in UCSC Genome Browser. Light blue highlighted regions indicate the predicted complex variant positions (Supplementary Data S4). Most of the predicted regions (5 out of 6) contained INDEL/MNLP complex genetic variants. (F = forward read, R = reverse read, C = combined forward and reverse reads)

a



# MNLP_1

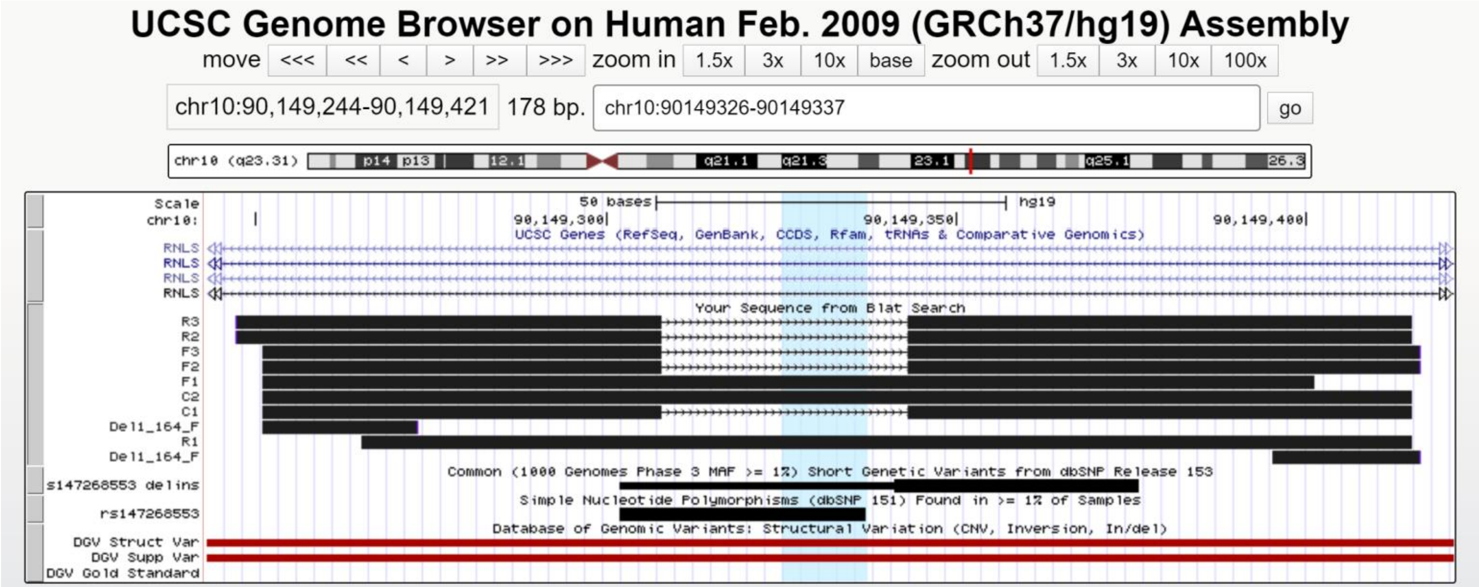
UCSC Genome Browser on Human Feb. 2009 (GRCh37/hg19) Assembly
move <<< << < > >> >>> zoom in 1.5x 3x 10x base zoom out 1.5x 3x 10x 100x
chr10:90,149,244-90,149,421 178 bp.
chr10:90149326-90149337
go
chr10 (q23.31)
Scale
50 bases
hg19
chr10:
90,149,300
90,149,350
90,149,400
UCSC Genes (RefSeq, GenBank, CCDS, Rfam, tRNAs & Comparative Genomics)
RNLS
Your Sequence from Blat Search
R3
R2
F3
F2
F1
C2
C1
Del1_164_F
R1
Common (1000 Genomes Phase 3 MAF >= 1%) Short Genetic Variants from dbSNP Release 153
s147268553 delins
Simple Nucleotide Polymorphisms (dbSNP 151) Found in >= 1% of Samples
rs147268553
Database of Genomic Variants: Structural Variation (CNV, Inversion, In/del)
DGV Struct Var
DGV Supp Var
DGV Gold Standard

b



# MNLP3_IRX4

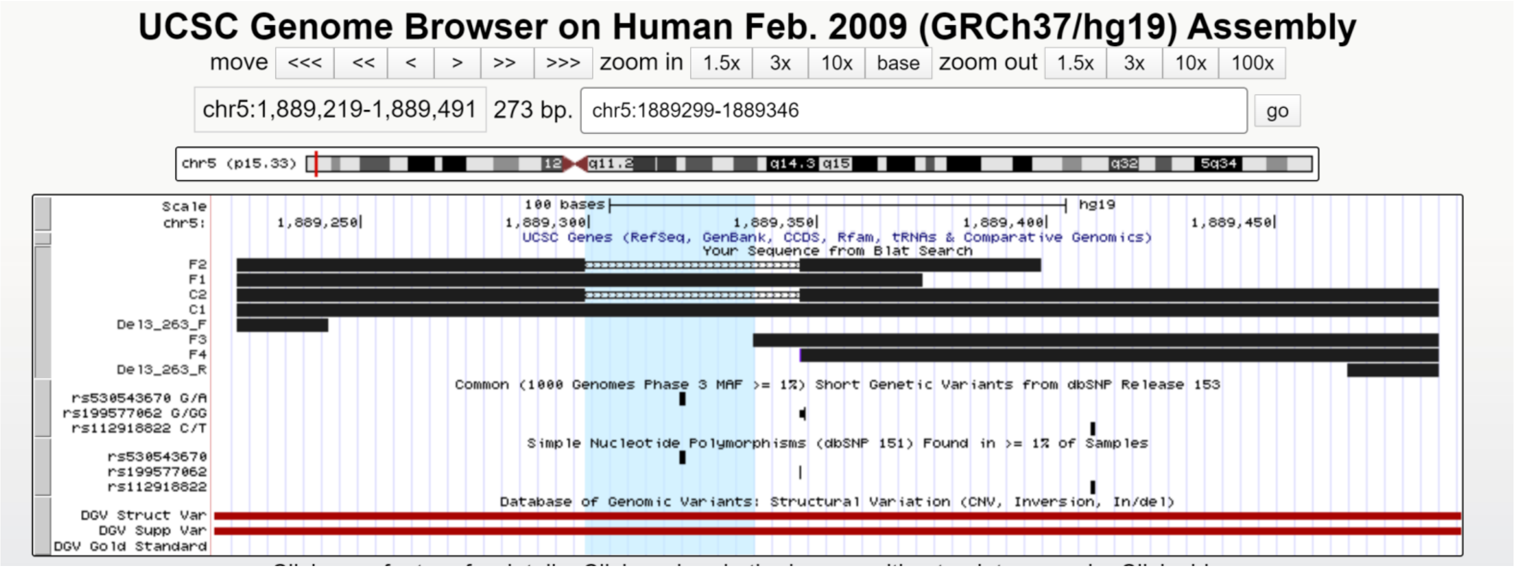
UCSC Genome Browser on Human Feb. 2009 (GRCh37/hg19) Assembly
move <<< << < > >> >>> zoom in 1.5x 3x 10x base zoom out 1.5x 3x 10x 100x
chr5:1,889,219-1,889,491 273 bp. chr5:1889299-1889346 go
chr5 (p15.33)
Scale
chr5:
100 bases
hg19
1,889,250
1,889,300
1,889,350
1,889,400
1,889,450
UCSC Genes (RefSeq, GenBank, CCDS, Rfam, tRNAs & Comparative Genomics)
Your Sequence from Blat Search
F2
F1
C2
C1
Del3_263_F
F3
F4
Del3_263_R
Common (1000 Genomes Phase 3 MAF >= 1%) Short Genetic Variants from dbSNP Release 153
rs530543670 G/A
rs199577062 G/GG
rs112918822 C/T
Simple Nucleotide Polymorphisms (dbSNP 151) Found in >= 1% of Samples
rs530543670
rs199577062
rs112918822
Database of Genomic Variants: Structural Variation (CNV, Inversion, In/del)
DGV Struct Var
DGV Supp Var
DGV Gold Standard

c

# MNLP6

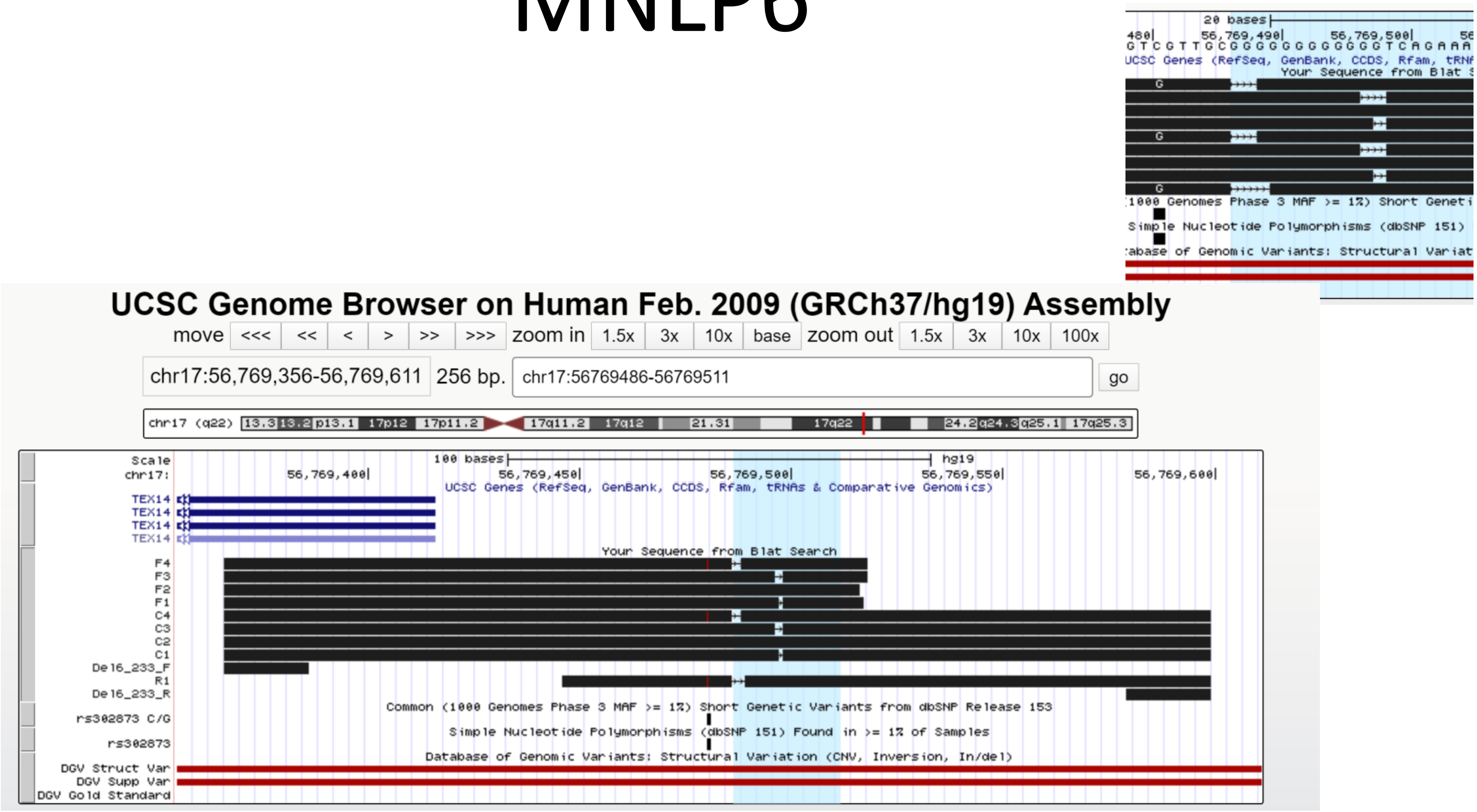
UCSC Genome Browser on Human Feb. 2009 (GRCh37/hg19) Assembly
move <<< << < > >> >>> zoom in 1.5x 3x 10x base zoom out 1.5x 3x 10x 100x
chr17:56,769,356-56,769,611 256 bp.
chr17:56769486-56769511
go
chr17 (q22)
Scale
chr17:
100 bases
hg19
56,769,400
56,769,450
56,769,500
56,769,550
56,769,600
UCSC Genes (RefSeq, GenBank, CCDS, Rfam, tRNAs & Comparative Genomics)
TEX14
TEX14
TEX14
TEX14
Your Sequence from Blat Search
F4
F3
F2
F1
C4
C3
C2
C1
Del6_233_F
R1
Del6_233_R
Common (1000 Genomes Phase 3 MAF >= 1%) Short Genetic Variants from dbSNP Release 153
rs302873 C/G
Simple Nucleotide Polymorphisms (dbSNP 151) Found in >= 1% of Samples
rs302873
Database of Genomic Variants: Structural Variation (CNV, Inversion, In/del)
DGV Struct Var
DGV Supp Var
DGV Gold Standard
20 bases
GTCGTTGCGGGGGGGGGGGGGTCAGAAA

d



# MNLP13

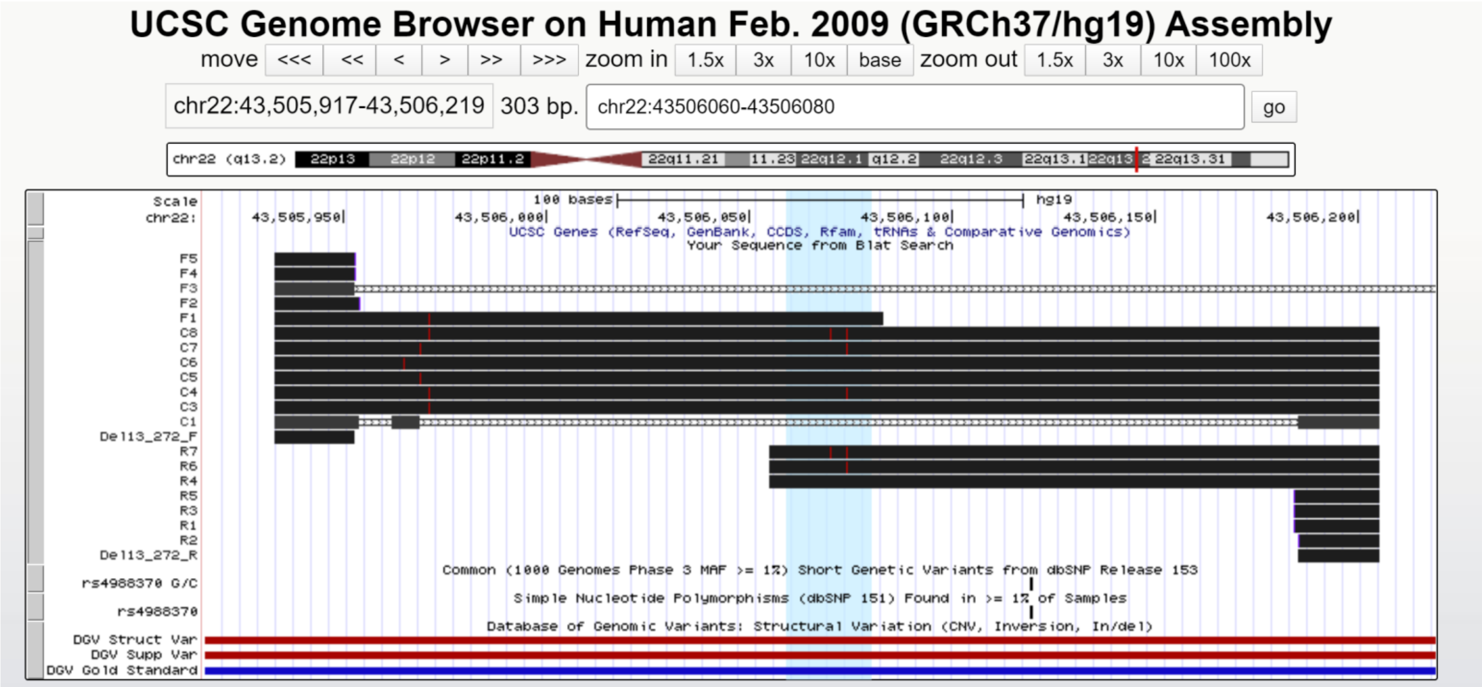
UCSC Genome Browser on Human Feb. 2009 (GRCh37/hg19) Assembly
move <<< << < > >> >>> zoom in 1.5x 3x 10x base zoom out 1.5x 3x 10x 100x
chr22:43,505,917-43,506,219 303 bp.
chr22:43506060-43506080
go
chr22 (q13.2)
Scale
chr22:
100 bases
hg19
43,505,950
43,506,000
43,506,050
43,506,100
43,506,150
43,506,200
UCSC Genes (RefSeq, GenBank, CCDS, Rfam, tRNAs & Comparative Genomics)
Your Sequence from Blat Search
F5
F4
F3
F2
F1
C8
C7
C6
C5
C4
C3
C1
Del13_272_F
R7
R6
R4
R5
R3
R1
R2
Del13_272_R
Common (1000 Genomes Phase 3 MAF >= 1%) Short Genetic Variants from dbSNP Release 153
rs4988370 G/C
Simple Nucleotide Polymorphisms (dbSNP 151) Found in >= 1% of Samples
rs4988370
Database of Genomic Variants: Structural Variation (CNV, Inversion, In/del)
DGV Struct Var
DGV Supp Var
DGV Gold Standard

e



# MNLP14

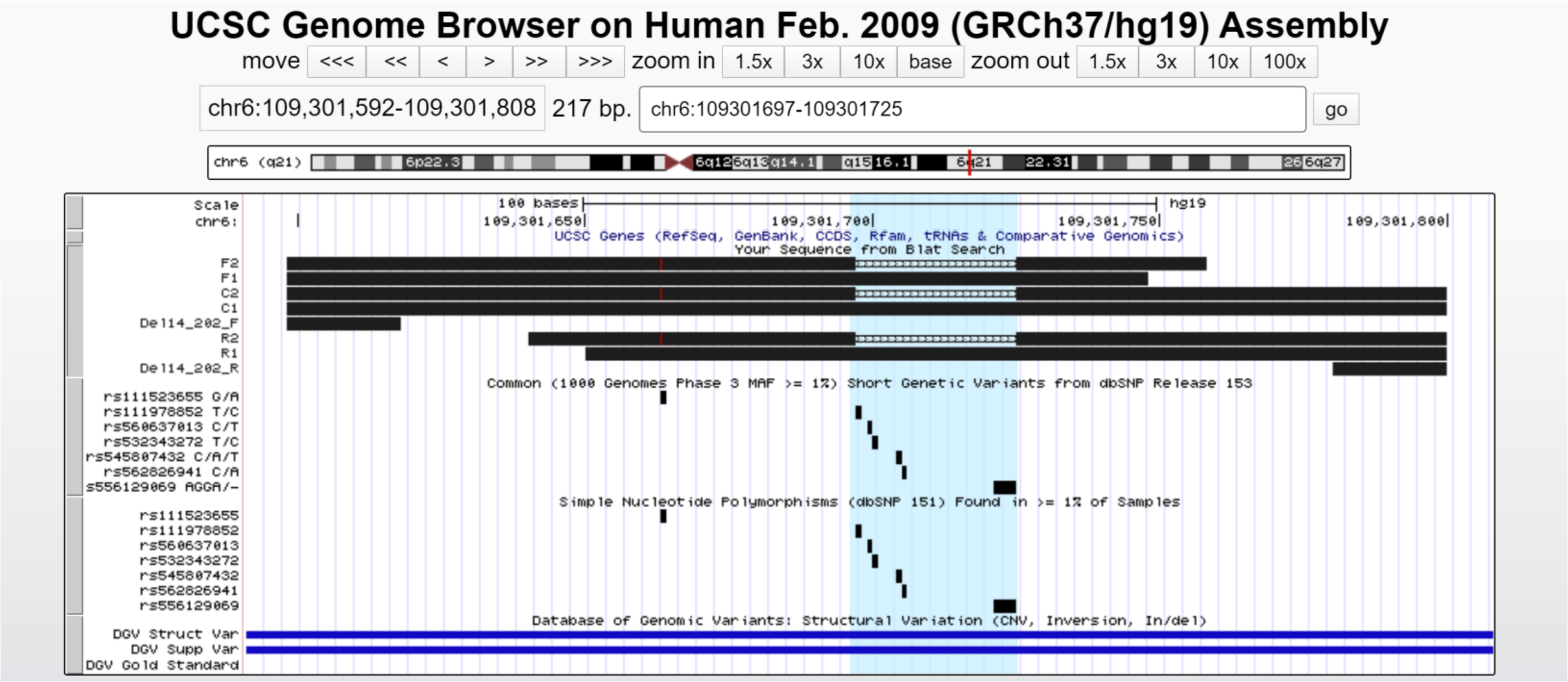
UCSC Genome Browser on Human Feb. 2009 (GRCh37/hg19) Assembly
move <<< << < > >> >>> zoom in 1.5x 3x 10x base zoom out 1.5x 3x 10x 100x
chr6:109,301,592-109,301,808 217 bp.
chr6:109301697-109301725
go
chr6 (q21)
Scale
chr6:
100 bases
hg19
109,301,650
109,301,700
109,301,750
109,301,800
UCSC Genes (RefSeq, GenBank, CCDS, Rfam, tRNAs & Comparative Genomics)
Your Sequence from Blat Search
F2
F1
C2
C1
Del14_202_F
R2
R1
Del14_202_R
Common (1000 Genomes Phase 3 MAF >= 1%) Short Genetic Variants from dbSNP Release 153
rs111523655 G/A
rs111978852 T/C
rs560637013 C/T
rs532343272 T/C
rs545807432 C/A/T
rs562826941 C/A
s556129069 AGGA/-
Simple Nucleotide Polymorphisms (dbSNP 151) Found in >= 1% of Samples
rs111523655
rs111978852
rs560637013
rs532343272
rs545807432
rs562826941
rs556129069
Database of Genomic Variants: Structural Variation (CNV, Inversion, In/del)
DGV Struct Var
DGV Supp Var
DGV Gold Standard

f

# MNLP16

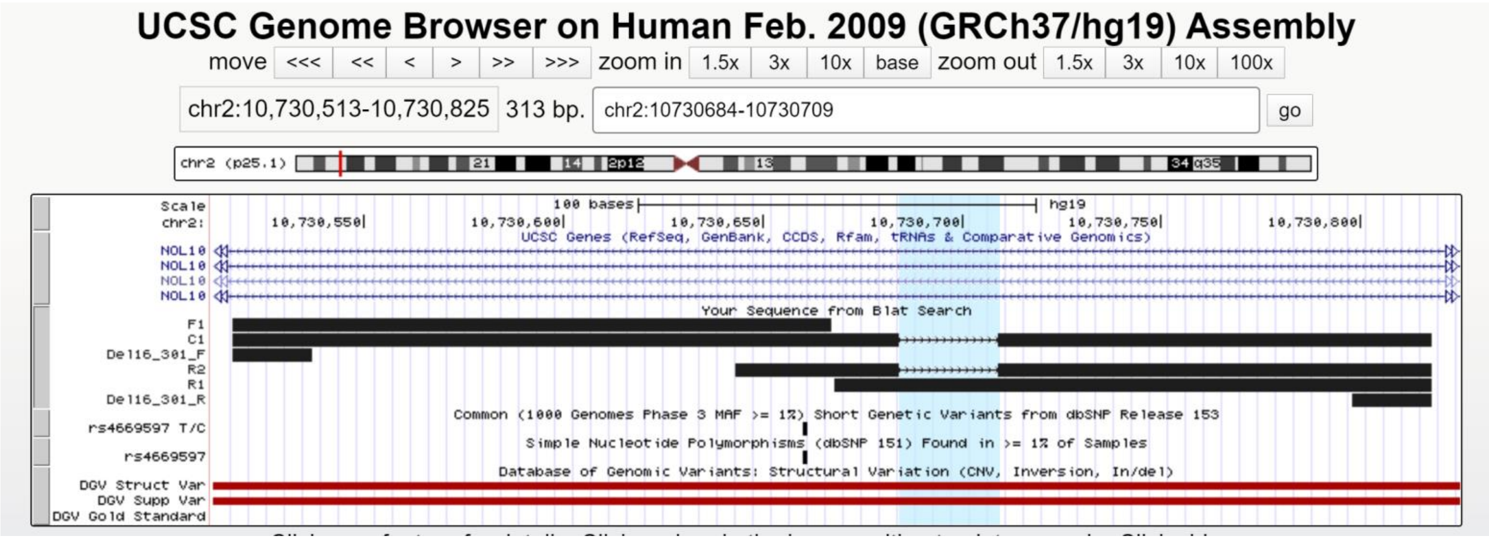

UCSC Genome Browser on Human Feb. 2009 (GRCh37/hg19) Assembly
move <<< << < > >> >>> zoom in 1.5x 3x 10x base zoom out 1.5x 3x 10x 100x
chr2:10,730,513-10,730,825 313 bp.
chr2:10730684-10730709
go
chr2 (p25.1)
Scale
chr2:
100 bases
hg19
10,730,550
10,730,600
10,730,650
10,730,700
10,730,750
10,730,800
UCSC Genes (RefSeq, GenBank, CCDS, Rfam, tRNAs & Comparative Genomics)
NOL10
NOL10
NOL10
NOL10
Your Sequence from Blat Search
F1
C1
Del16_301_F
R2
R1
Del16_301_R
Common (1000 Genomes Phase 3 MAF >= 1%) Short Genetic Variants from dbSNP Release 153
rs4669597 T/C
Simple Nucleotide Polymorphisms (dbSNP 151) Found in >= 1% of Samples
rs4669597
Database of Genomic Variants: Structural Variation (CNV, Inversion, In/del)
DGV Struct Var
DGV Supp Var
DGV Gold Standard

# Supplementary Methods

## Genotyping and verification of the MNLP alleles

The dbSNP Build 151 (2017) database has been described 44 different polymorphisms (19 MNLPs/INDELs and 25 SNPs) at the region encompassing the MNLP position thus highlighting complexity in annotating polymorphisms in this region (**Supplementary Figure 1b**). Based on our genotyping data (**Supplementary Figure 1c-f**) and prior literature [1], we hypothesized that this region harbored a biallelic (L and S alleles) complex (MNLP) variant (**Supplementary Figure 1a**) and that the other annotated polymorphisms were false positive variants. Indeed, the most dbSNP Build 155 (2021) contains only two variants (rs530534670 and rs199577062) (**Supplementary Methods Figure SM1a**). These variants appear not to be actual polymorphisms, but rather represent sequence differences based on the S and L alleles. We used different methods to prove, that the L and S alleles are the real and functional alleles. We determined the relationship between the MNLP alleles and the previously annotated short polymorphisms (rs530534670 and rs199577062).

Genotyping the MNLP in four PCa cell lines by amplicon sequencing confirmed the existence of the S and L alleles (**Supplementary Figure S1c**). This analysis revealed, that LNCaP is homozygous for the S allele, VCaP and PC-3 are homozygous for the L allele, and 22Rv1 is heterozygous (**Supplementary Figure S1d).** We also observed the existence of the S and L alleles in different WGS data collection, including gnomAD browser and our 22Rv1 WGS data (**Supplementary Methods Figure SM1b and c**). This was further confirmed by TCGA data coverage and MNLP genotyping analysis (See: **"Coverage based analysis"** for detailed explanation). To clarify which variant exists in the population, we performed deep amplicon sequencing in pooled human germline samples (n=62) and in an additional cohort of 56 individual clinical samples. These analyses confirmed the existence of a single biallelic MNLP variant with L and S alleles in the human population (**Supplementary Figure S1a**).

Comparison of the L and S alleles at base pair resolution by sequence alignment revealed, that the 21 bp S allele is not an unambiguous derivative of the 47 bp L allele, which can be simply explained by a 26 bp deletion. There is a 21 bp core region within the L allele, which shows 12 nucleotide identity (57%) with the S allele. The two

sequences are similar, but not identical (**Supplementary Methods Figure SM1d**). Therefore, instead of calling this variant as an insertion deletion (INDEL) variant, we used the multiple nucleotide variant (MNLP) term [2, 3].

### Data from 1000 Genomes Project also confirm the existence of the INDEL

To verify the existence of the L and S alleles in 1000 Genomes Project dataset (**Supplementary Table S6**), we downloaded and realigned 20 high coverage bam files to both L and S allele reference sequence (**Supplementary Table S1**). This analysis revealed, that 100% of the reads from homozygous G/G samples at the rs199577062 position aligned (with base pair accuracy) to the L allele reference genome (**Supplementary Methods Figure SM2**). (A representative G/G genotype sample at the rs199577062 position indicated by (*) on the figure shows perfect alignment for the L reference sequence, but not the S reference sequence). Similarly, GG/GG samples at the rs199577062 position are perfectly aligned to the S reference genome, but not to the L reference sequence. (A representative GG/GG sample indicated by (***). In case of G/GG samples at the rs199577062 position, ~50% of the reads perfectly matched to the S reference, the other 50% perfectly aligned to the L reference, indicating, that the real and full-length alleles are the 47 bp L and 21 bp S MNLP alleles. (A representative G/GG sample indicated by (**). All three representative samples alignment summarized on **Supplementary Methods Figure SM3** with detailed explanation for each scenario. **Supplementary Methods Figure SM4-6** are showing the nucleotide level resolution alignment of the three representative individual samples against the L and S reference sequence.

### The rs199577062 variant is a surrogate for the MNLP

Next, we compared rs530543670, rs199577062 and MNLP genotypes from 1000 Genomes Project data to determine their relationship. We used 1000 Genomes Project genotype data at rs530543670 and rs199577062 positions, furthermore MNLP genotypes from the downloaded 1000 Genomes Project bam files after aligned to L and S reference sequences to genotype the MNLP position (**Supplementary Table S1 and S6**). This analysis revealed, that both rs530543670 and rs199577062 variants could accurately distinguish the MNLP genotypes. Our results show that the rs199577062 genotypes correspond to the MNLP genotypes. Base pair level

comparison revealed, that rs199577062 alleles represent parts of the L and S alleles (**Supplementary Methods Figure SM7a**). The “G” allele of rs199577062 variant correspond to the 3` end of the 47 bp L allele, while the “GG” allele aligns to the 3` end of the 21 bp S allele. Thus, the “G/GG” alleles of the rs199577062 are simply proxies for the L/S alleles (**Supplementary Methods Figure SM7b**). We concluded, that rs199577062 genotypes are accurately genotyping the samples, but the real alleles are the S and L alleles.

A recent prostate GWAS study, Dadaev et. al. identified rs530543670 and rs199577062 as the statistically most significant variants influencing the IRX4 gene expression and that they highly correlate with rs12653946 [4]. When we conditioned these variants on the MNLP genotype, the which diminished the effect of rs530543670 and rs199577062 (**Supplementary Method Figure SMF7c**). This observation also clarifies the relationship between the MNLP and rs530543670/rs199577062 genotypes.

**Large scale identification of possible correlated complex variants**

In order to estimate the number of PCa risk loci which has possible correlated complex variants we made a genome wide survey to use all PCa risk loci. We used coverage data information from aligned WGS BAM files as a surrogate to delineate potential correlated complex variant positions within GWAS haploblocks.

**Data source**

We analyzed total 1679 germline TCGA WGS samples and downloaded aligned reads for 147 regions (bam slices) for genotyping, coverage based complex variant analysis and read realignment analysis (detailed here).

In total, 147 PCa risk loci [1] were involved in this analysis. The maximum span of each haploblock were determined (based on SNP correlation) and the corresponding aligned bam file parts were downloaded. Each sample were genotyped and grouped according to the reported leading SNP. The coverage was compared and correlated within the leading SNP based genotypes.

We used 10x coverage filter for each locus at the corresponding leading SNP and complex variant region. This resulted 1310 germline TCGA WGS data (bam slices)

out of the 1679 available samples for the IRX4 region. Only 121 of these samples had gene expression data from PC patients.

**Demonstrating the power of the coverage based complex variant identification analysis from TCGA data at the IRX4 locus**

Our working hypothesis was, that in general, the average coverage pattern is similar (constant) among different random sample groups (**Supplementary Methods Figure SM8a**). However, in case of the MNLP, the S allele containing reads cause significant drop in the coverage (practically close to zero), because the hg19 human reference genome contains the L allele sequence. This scenario is demonstrated on **Supplementary Methods Figure S6** upper panel, in case of the *** NA12777 (S/S) 1000 Genomes Project S allele containing sample which was aligned against the L reference sequence. Therefore, if we group the samples based on their genotype (ie.: leading SNP of a risk loci), which reflects the MNLP status (due to the genetic correlation), we should see differences between the two group coverages at the complex variant position (if the complex variant in high LD with the leading SNP), but not in the surrounding regions (**Supplementary Methods Figure SM8b**). In order to test this hypothesis, we interrogated the IRX4 MNLP region and after we involved all 147 PCa risk loci and validated our findings by short read amplicon sequencing and read alignment against the hg19 human reference genome.

**Coverage based analysis to identify potential correlated complex variants**

Our coverage-based analysis of the IRX4 region revealed that the coverage fluctuation at a certain nucleotide position is relatively constant across different samples. Taking two random group of TCGA germline samples we observed no differences between coverage fluctuations at the IRX4 MNLP region (**Supplementary Methods Figure SM8a**).

However, when we grouped the samples based on their leading SNP (i.e.: rs12653946 statistically most significant SNP) genotype status (“C” reference vs. “T” mutant alleles) as expected we observed huge deviation in coverage at the MNLP position between the two groups (**Supplementary Methods Figure SM8b**).

By comparing the two previous analysis, we concluded, that systematic genotype-based coverage analysis could delineate potential genetically correlated complex variants as we observed at the IRX4 locus (**Supplementary Methods Figure SM8c**).

**Performing the coverage based complex variant (MNLP) screen at whole genome level to include all existing PCa risk loci**

Next, we planned to analyze all existing PCa risk loci for possible correlated complex variants which could explain disease risk association. As of today, GWAS studies have been identified 147 prostate cancer susceptibility loci [5]. However, some of these regions has no fine mapping data available. Therefore, in order to identify possible correlated complex variants, first we determined existing haploblock sizes for each region (**Supplementary Methods Figure SM9 and SM10**).

**Determining haploblock sizes and apply for all 147 interrogated loci**

Using the reported statistically most significant SNPs correlated SNPs were determined by using the data of the 1000 Genomes Project. Squared Pearson correlations were calculated in the +/- 1.5 million base pairs neighborhood of all the 147 leading SNPs with the proxysnp package [6]. The region of interest (ROI) - where potential correlated MNLPs can be located - was calculated, as the widest range that has SNP with squared Pearson correlation, larger than 0.2 at both end (**Supplementary Methods Figure SM9**).

Not to miss any potential correlated MNLPs, at least +/- 100.000 base pairs were considered in both directions even if the correlation was lower at that range (**Supplementary Methods Figure SMF10**).

In this way 147 ROI was obtained around the 147 leading SNPs and BAM coverage data (pileups) were downloaded for these regions in case of 1679 TCGA normal germline WGS samples. Also, VCF files were collected for these patients that made possible genotyping of the leading SNP positions.

**Coverage based genotyping**

For coverage-based genotyping two groups were created based on the leading SNPgenotypes: homozygous reference, and the others (called mutants). Using this grouping strategy, the BAM coverages were summed for each ROIs resulting in reference coverage and mutant coverage. Comparing these coverages differences

variable regions carrying potential correlated complex variants were identified. The signal was calculated as the $\log_{10}$ ratio of the mutant coverage and the reference coverage ($\log_{10}$ (mutant coverage/reference coverage). The median of this signal was calculated in a rolling window of 500 base pairs. For each ROI the standard deviation of the signal was calculated, and variable regions were identified if the difference between the rolling median and the signal was larger than 10 times of the standard deviation for more than 10 consecutive base pairs. In total 135 of variable regions were identified (**Supplementary Table S3**). In order to select functional candidates and reduce the number of potential candidates, the 135 MNLP positions were intersected with the Cistrome database whole human TF dataset which contains more than 230 million TF peaks from multiple cell lines and human tissue samples [7]. This filtering resulted 96 MNLP candidates, which has overlap at least 1 active epigenetic region (TF peaks). **Supplementary Method Table 1** is listing the top 10 MNLP candidates. Column 7 (CistromeTrackNum) shows the number of overlapping TF peaks at the predicted MNLP position. Table also shows potential complex variant coordinates, related leading SNP (headMut), estimated complex variant size and existing variants overlap with complex variants according to the dbSNP151.

**Supplementary Method Table SM1. List of top 10 complex variant candidates shows high correlation with the leading SNP and overlap with active epigenetic regions (Extracted from Supplementary Table S3).**

| $MNLP_{stop}$ | $MNLP_{start}$ | headMut | chr | peaklen | CistromeTrackNum | dbSNP151_entry |
|---|---|---|---|---|---|---|
| 90149337 | 90149326 | rs1935581 | chr10 | 11 | 571 | 4 |
| 43706024 | 43705864 | rs4711748 | chr6 | 160 | 320 | 111 |
| 1889346 | 1889299 | rs12653946 | chr5 | 47 | 241 | 44 |
| 57372701 | 57372682 | rs33984059 | chr15 | 19 | 192 | 8 |
| 108218197 | 108218177 | rs1800057 | chr11 | 20 | 164 | 5 |
| 56769511 | 56769486 | rs2680708 | chr17 | 25 | 147 | 33 |
| 134178170 | 134178112 | rs878987 | chr11 | 58 | 98 | 42 |
| 113083330 | 113083311 | rs7611694 | chr3 | 19 | 93 | 6 |
| 49640020 | 49639994 | rs10875943 | chr12 | 26 | 72 | 8 |
| 108206137 | 108206118 | rs1800057 | chr11 | 19 | 51 | 4 |

**Complex variant discovery and confirmation by amplicon sequencing**

Sixteen out of the 96 INDEL candidate regions were amplified by specific primer pairs to generate 200-320 bp amplicons using Coriell pooled human DNA samples (**Supplementary Table S6**). Amplicons were subjected for deep sequencing using 150 bp paired end sequencing chemistry (150PE). Unique sequence variants were collected, and frequencies were calculated and rank ordered for each variants. MNLP/INDEL positions, where two dominant variants ratio was above 99% of the total variants were considered as biallelic MNLPs/INDELs (**Supplementary Figure S10a-i**).

We identified three INDELs – including the MNLP from the IRX4 region – which showed high correlation with the corresponding leading SNP and biallelic characteristics (**Figure 7 and Supplementary Figure S9**).

Next, we genotyped these INDELs using BAM slices from TCGA WGS samples and artificial reference genomes at the INDEL position created by the most frequent reads from the amplicon sequencing.

**Complex variant genotyping by read realignment from bam slices**

Blood derived normal samples with WGS data were selected from the TCGA legacy archive on the SevenBridges cloud portal. From the BAM files of these 1679 samples the MNLP regions with additional +/-500 flanking nucleotides were downloaded and converted to FASTQ with bamtofastq command of bedtools. The resulting reads were aligned to all the corresponding MNLP variant reference sequences. For genotyping samples with smaller than coverage 10 were discarded from the analysis. MNLP genotypes were called based on the allele ratio of unique nucleotides in each variant.

**Testing disease risk associations of complex variant genotypes**

For each MNLP considered, we used germline genotypes from TCGA samples to train a genetic predictor. MNLP genotype was treated as a “phenotype”; all common (MAF>1%), well imputed SNPs in a mega base around the complex variant were used as features; and a penalized regression LASSO model was trained to predict the phenotype from the SNP genotypes. The LASSO model was highly predictive across all tested MNLPs, as computed by cross-validation of the predictor (mean cross-validation $R^2$ = 0.64; median cross-validation p-value = 1e-128). We then integrated

each predictive model with the PCa GWAS summary data to infer the expected association between the complex variant genotype and PCa risk using an analog of the TWAS test. We used summary-based conditional analysis [8] to condition all SNPs in the GWAS locus on the predicted complex variant and quantify the residual association. As an independent statistical confirmation, we additionally applied colocalization analysis using the COLOC test [9] to evaluate the probability that the complex variant and the GWAS share the same causal variant, using all SNP-MNLP associations in the locus as inputs. This analysis resulted 3 complex variants (including the indel at the *IRX4* region, listed MNLP 3 in **Supplementary Table S4, Supplementary Method Table SM1**) which showed epigenetic activity and can be potential causal variant candidate (**Figure 7** and **Supplementary Figure S9**).

# Supplementary Method References


1. Nguyen, H.H., et al., *IRX4 at 5p15 suppresses prostate cancer growth through the interaction with vitamin D receptor, conferring prostate cancer susceptibility.* Hum Mol Genet, 2012. **21**(9): p. 2076-85.
2. Wakeling, M.N., et al., *Misannotation of multiple-nucleotide variants risks misdiagnosis.* Wellcome Open Res, 2019. **4**: p. 145.
3. Wang, Q., et al., *Landscape of multi-nucleotide variants in 125,748 human exomes and 15,708 genomes.* Nat Commun, 2020. **11**(1): p. 2539.
4. Dadaev, T., et al., *Fine-mapping of prostate cancer susceptibility loci in a large meta-analysis identifies candidate causal variants.* Nat Commun, 2018. **9**(1): p. 2256.
5. Schumacher, F.R., et al., *Association analyses of more than 140,000 men identify 63 new prostate cancer susceptibility loci.* Nat Genet, 2018. **50**(7): p. 928-936.
6. ; Available from: https://github.com/slowkow/proxysnps.
7. Liu, T., et al., *Cistrome: an integrative platform for transcriptional regulation studies.* Genome Biol, 2011. **12**(8): p. R83.
8. Gusev, A., et al., *Transcriptome-wide association study of schizophrenia and chromatin activity yields mechanistic disease insights.* Nat Genet, 2018. **50**(4): p. 538-548.
9. Giambartolomei, C., et al., *Bayesian test for colocalisation between pairs of genetic association studies using summary statistics.* PLoS Genet, 2014. **10**(5): p. e1004383.

# Supplementary Method Figures

**Supplementary Method Figure SMF1.**

**a.** The latest dbSNP Build 155 (2021) contains two variants (rs530534670 and rs199577062) at the MNLP position.

**b.** WGS data collection (gnomAD data base) aligned to the human reference genome (containing the L allele) indicates the existence of the MNLP.

**c.** 22Rv1 PCa cell line (heterozygous at the MNLP position (**Supplementary Figure S1c**) WGS coverage data (aligned to hg38 L allele containing reference genome) indicates the presence of both S and L alleles in this cell line.

**d.** Alignment shows similarity (red letters) and differences (blue and black letters) between the L and S allele, indicating the complexity of these MNLP alleles.

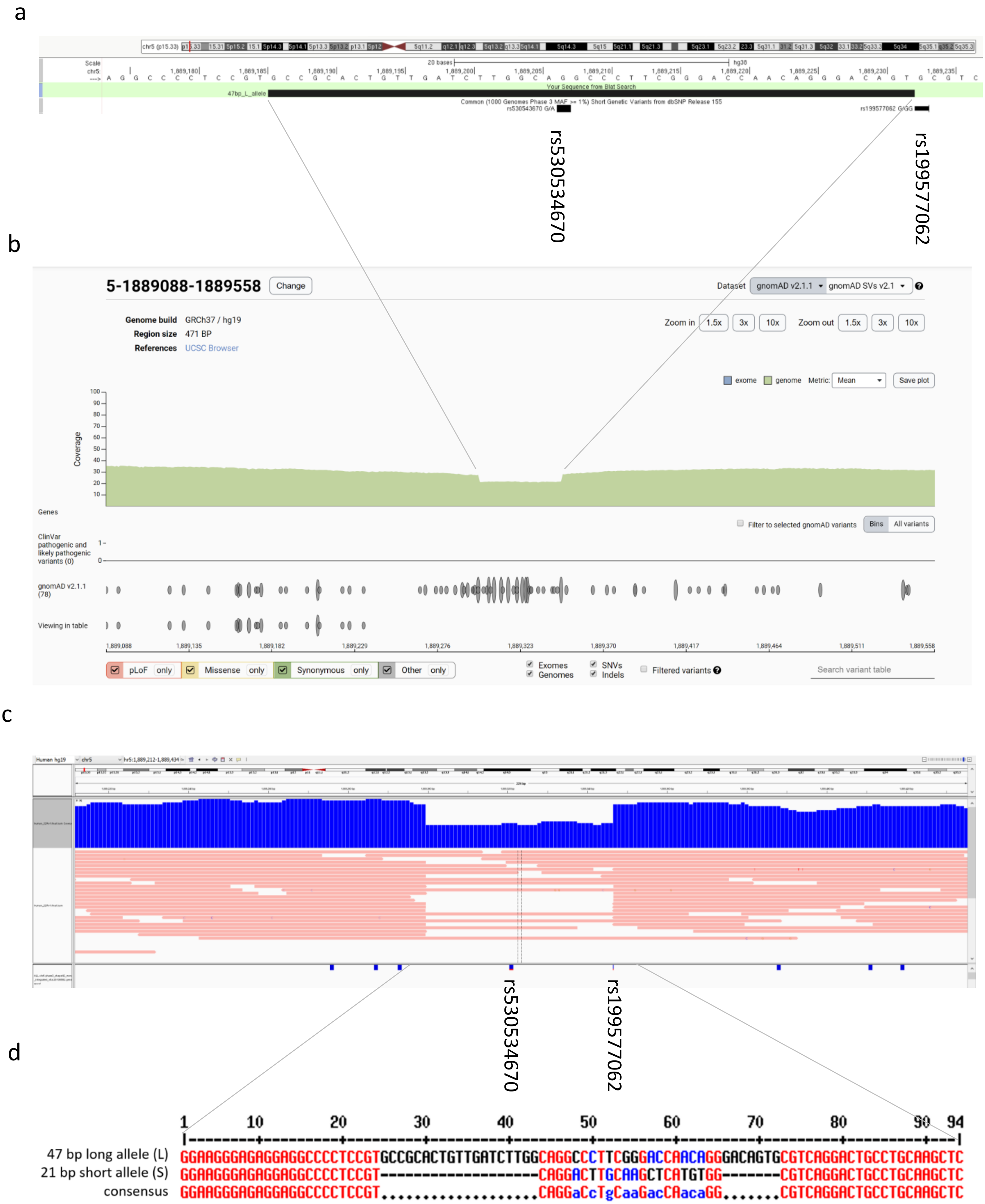
a
Your Sequence from Blat Search
47bp_L_allele
Common (1000 Genomes Phase 3 MAF >= 1%) Short Genetic Variants from dbSNP Release 155
rs530534670 G/A
rs199577062 G/GG
rs530534670
rs199577062
b
5-1889088-1889558
Change
Genome build GRCh37 / hg19
Region size 471 BP
References UCSC Browser
Dataset gnomAD v2.1.1 gnomAD SVs v2.1
Zoom in 1.5x 3x 10x
Zoom out 1.5x 3x 10x
exome genome Metric: Mean
Save plot
Coverage
Genes
ClinVar pathogenic and likely pathogenic variants (0)
gnomAD v2.1.1 (78)
Viewing in table
Filter to selected gnomAD variants
Bins All variants
pLoF only
Missense only
Synonymous only
Other only
Exomes
Genomes
SNVs
Indels
Filtered variants
Search variant table
c
Human hg19
chr5
rs530534670
rs199577062
d
47 bp long allele (L) GGAAGGGAGAGGAGGCCCCTCCGTGCCGCACTGTTGATCTTGGCAGGCCCTTCGGGACCAACAGGGACAGTGCGTCAGGACTGCCTGCAAGCTC
21 bp short allele (S) GGAAGGGAGAGGAGGCCCCTCCGT-------------------CAGGACTTGCAAGCTCATGTGG-------CGTCAGGACTGCCTGCAAGCTC
consensus GGAAGGGAGAGGAGGCCCCTCCGT...................CAGGaCcTgCaaGacCAacaGG.......CGTCAGGACTGCCTGCAAGCTC

**Supplementary Method Figure SMF2.**

Sequence realignment of 20 1000 Genomes Project WGS data using the L and S allele reference sequences. Highlighted samples (red rectangle) show representative examples for the three possible L/L (*), L/S (**) and (S/S (***) MNLP genotypes.

Spisak et al., Supplementary Methods Figure SM2

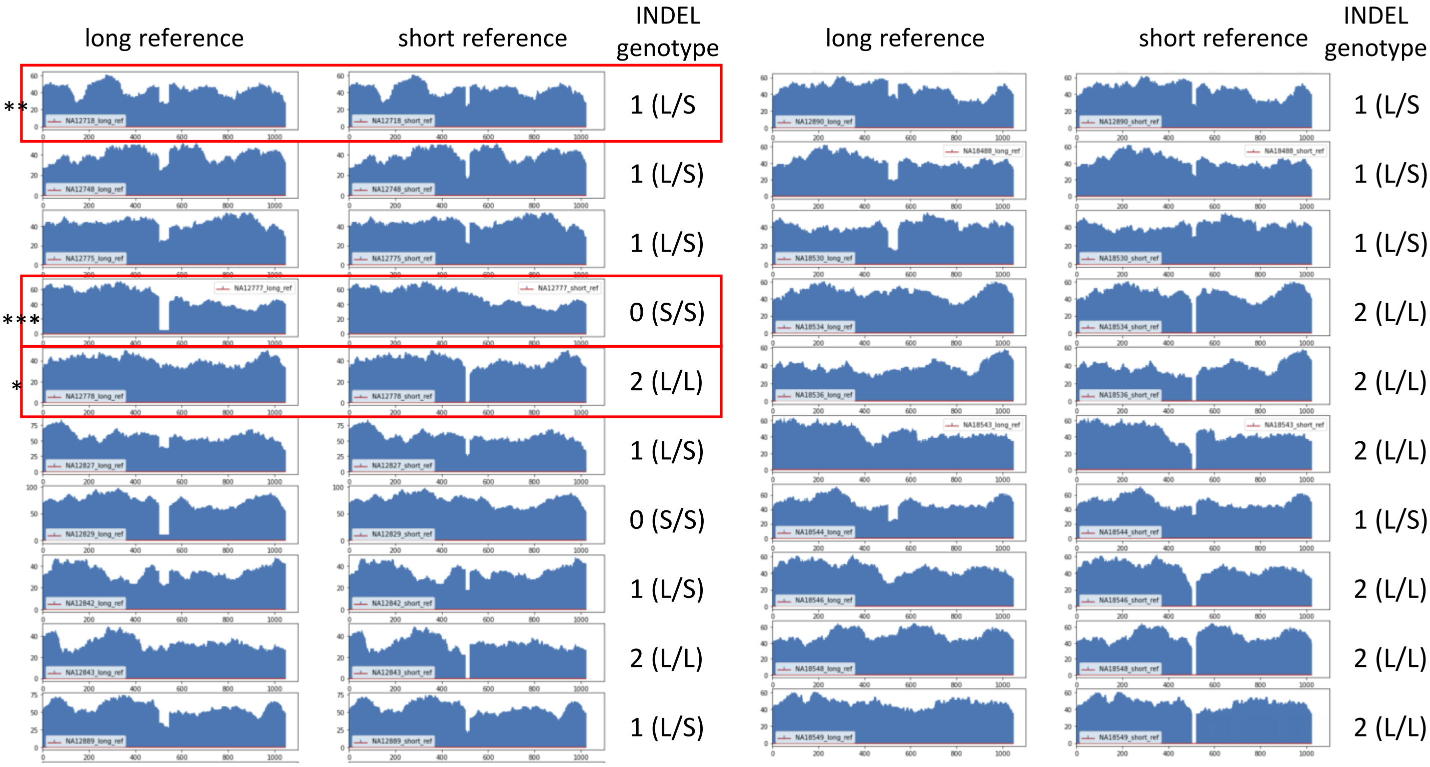

**Supplementary Method Figure SMF3.**

Alignment results of the three selected representative genotypes (* = NA12778 (L/L), ** = NA12718 (L/S), *** = NA12777 (S/S)) against the L (upper panel) and S (bottom panel) reference sequences and the detailed explanation for the alignment outcomes.

* NA12778 (L/L) rs199577062 G/G

** NA12718 (L/S) rs199577062 G/GG

*** NA12777 (S/S) rs199577062 GG/GG

Long reference

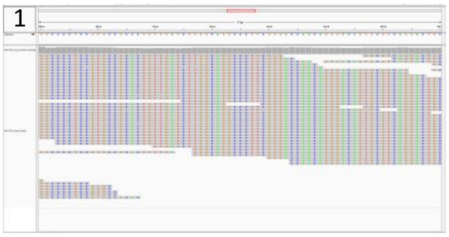


100% of the reads match perfectly to the long reference sequence, **verifying the L/L** genotype

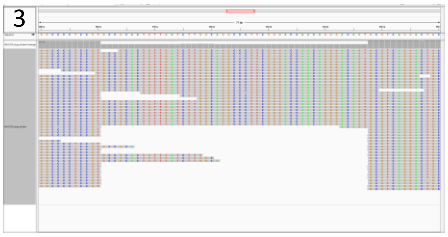


~50% of the reads match perfectly to the long reference sequence, **indicating the L/S** genotype

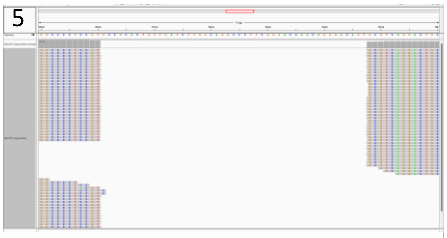


Non of the reads match to the long reference sequence, **suggesting the S/S** genotype. Note, some of the S allele carrying reads could partially fit to the long reference, due to the small homology between the S and L alleles, depending on the alignment parameters.

Short reference

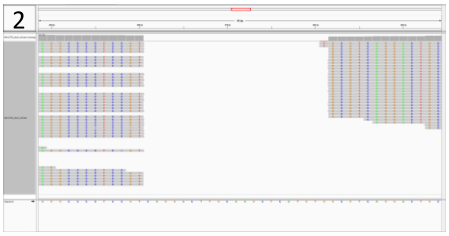


Non of the reads match to the short reference sequence, **supporting the L/L genotype**

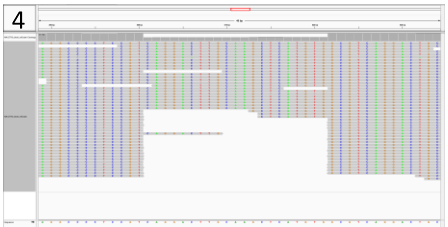


~50% of the reads match perfectly to the short reference sequence, **confirming the L/S** genotype

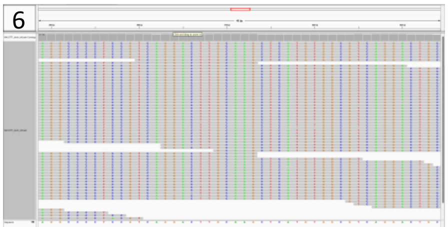


100% of the reads match perfectly to the short reference sequence, **verifying the S/S** genotype

**Supplementary Method Figure SMF4.**

IGV plots of the (* = NA12778) homozygous L allele containing sample after realignment to the L (upper panel) and S (bottom panel) reference sequences.

Spisak et al., Supplementary Methods Figure SM4

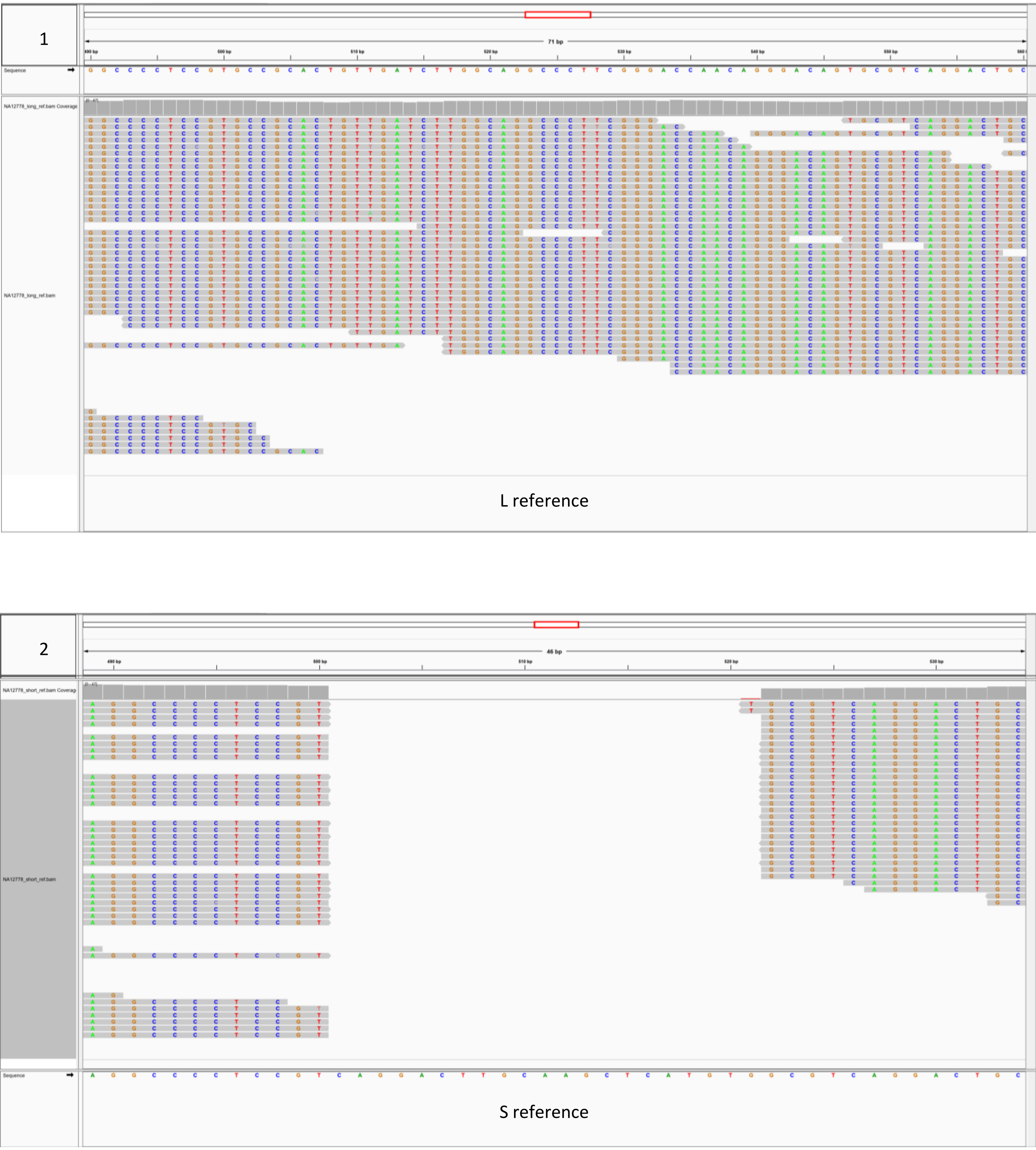

**Supplementary Method Figure SMF5.**

IGV plots of the (** = NA12718) heterozygous sample at the MNLP position after realignment to the L (upper panel) and S (bottom panel) reference sequences.

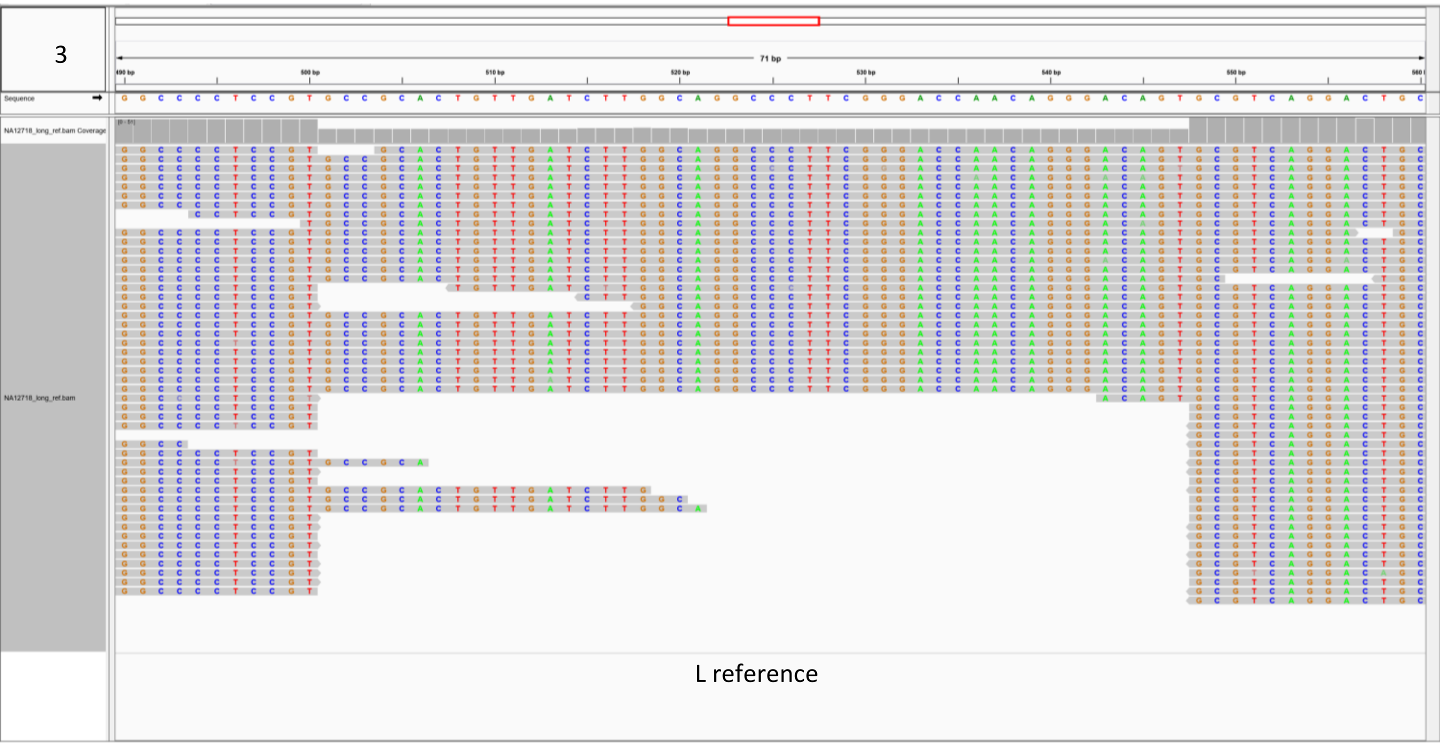


L reference

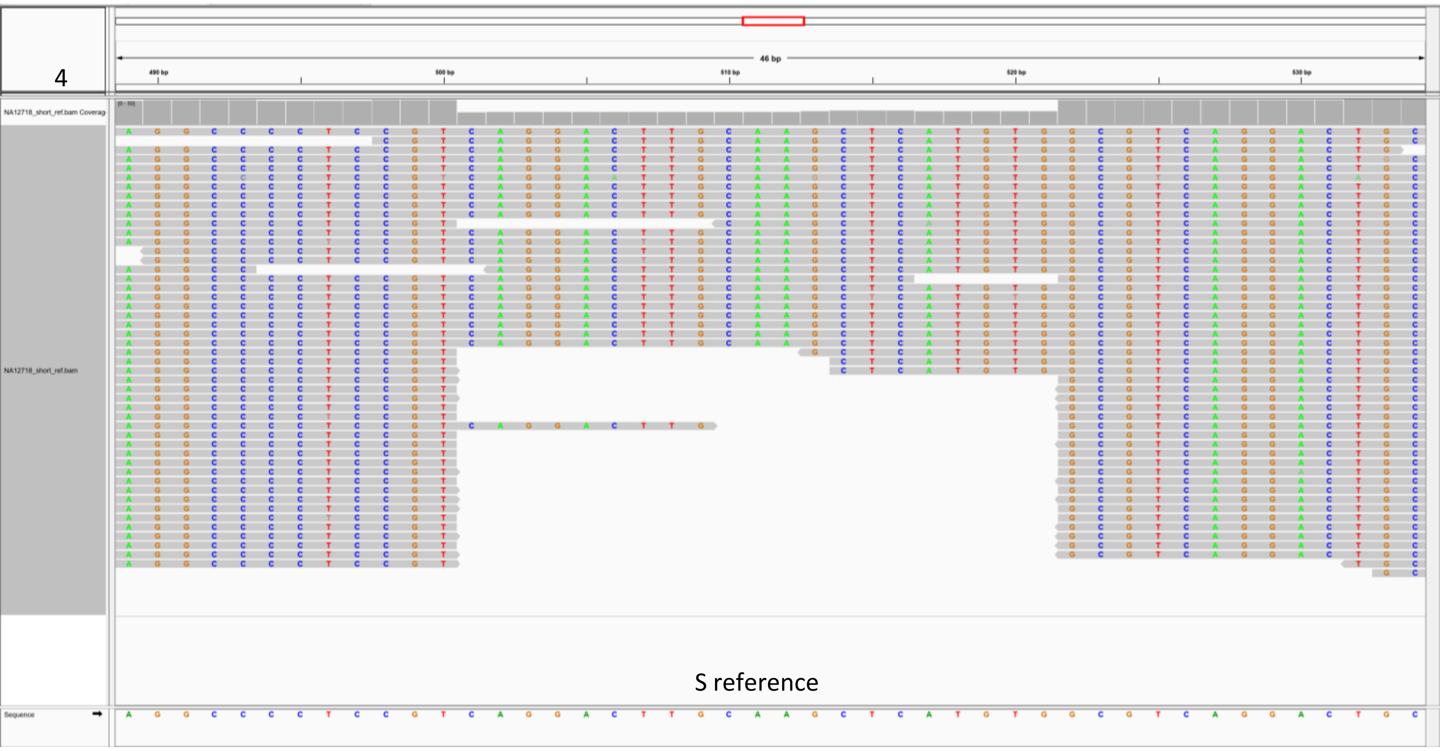


S reference

**Supplementary Method Figure SMF6.**

IGV plots of the (*** = NA12777) homozygous S allele containing sample after realignment to the L (upper panel) and S (bottom panel) reference sequences.

Spisak et al., Supplementary Methods Figure SM6

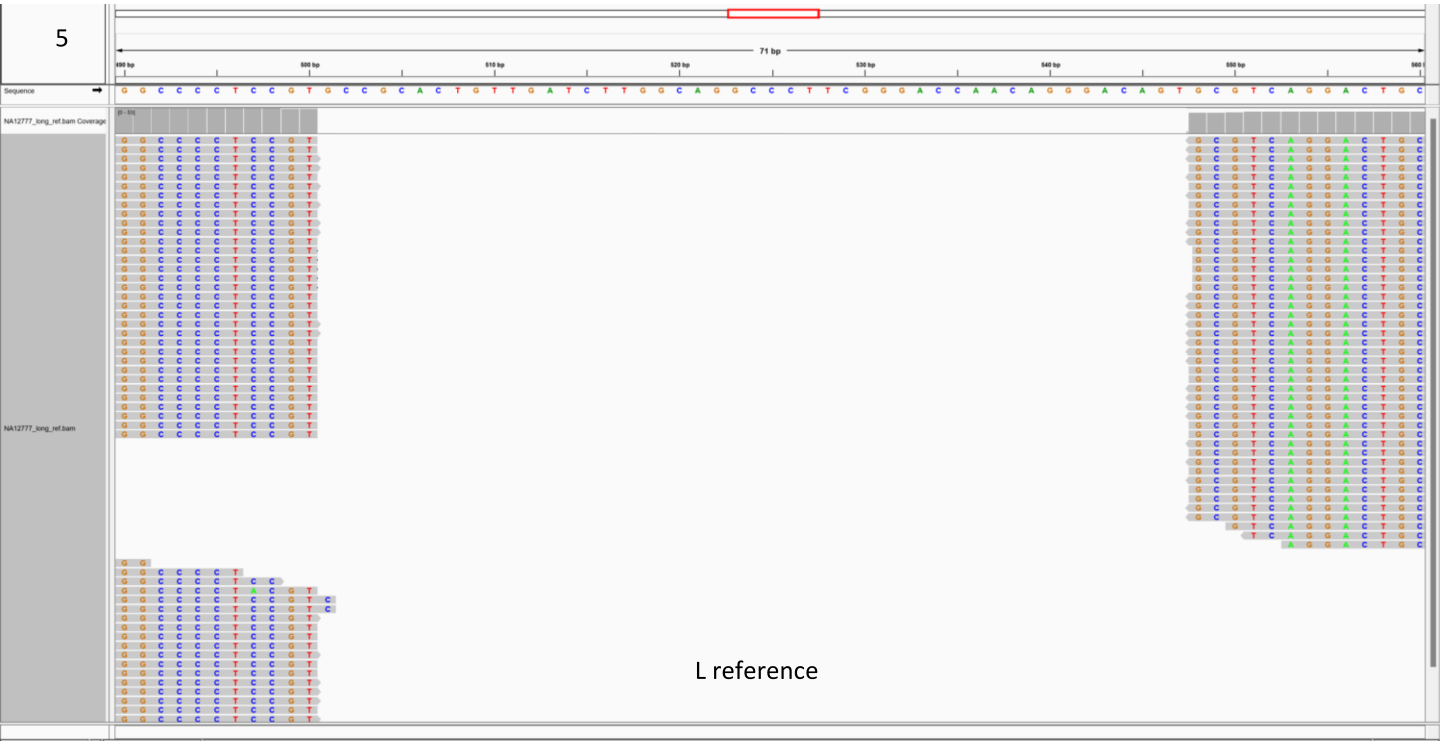


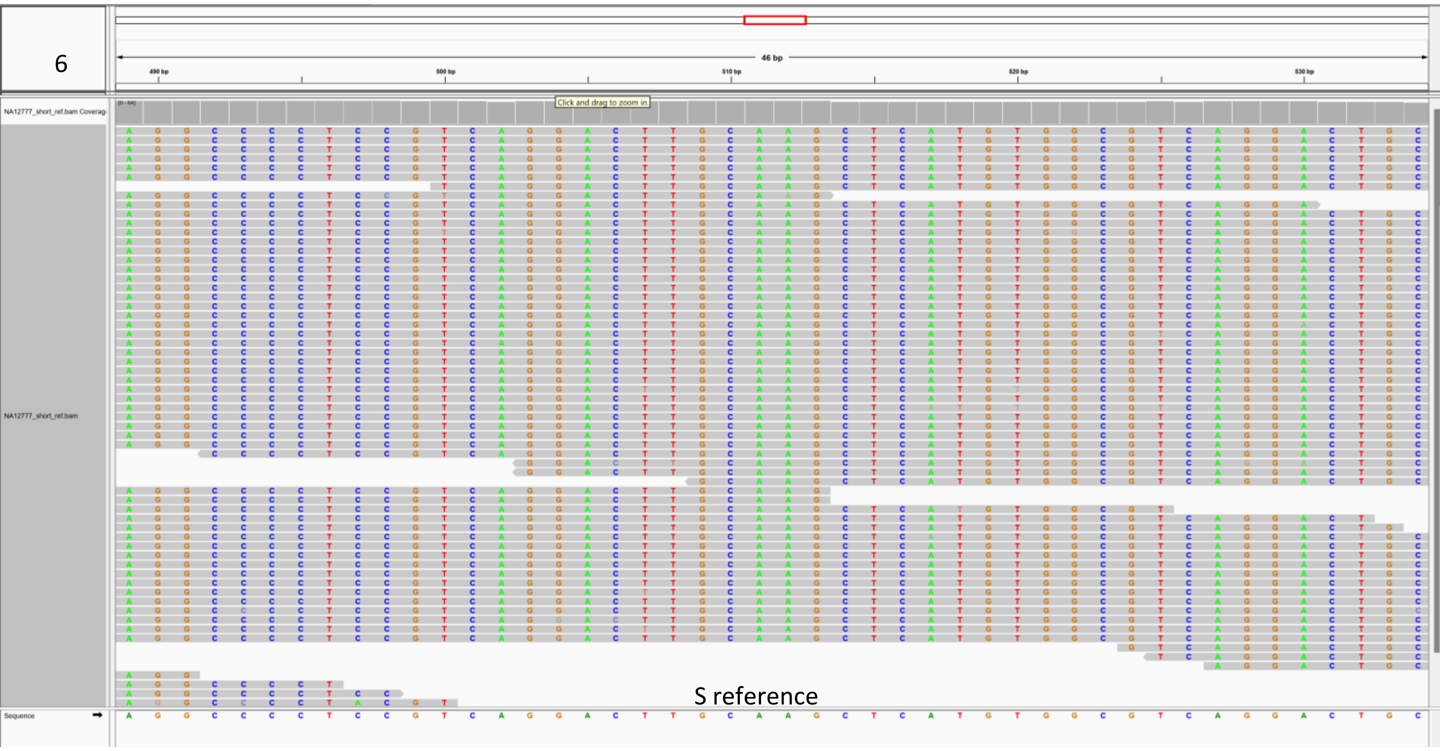

**Supplementary Method Figure SMF7.**

**a.** Genotype summary of the 20 interrogated 1000 Genomes Project sample at the rs530534670, rs199577062 and the MNLP positions.

**b.** Nucleotide level analysis explains the relationship between MNLP and rs199577062 alleles. The GG allele is part of the S allele, while the G allele is part of the L allele.

**c.** GWAS Manhattan plot of the *IRX4* locus. Genome-wide significant associations found by Dadaev et. al., indicated by two associations exceeding the dashed line at $P = 5 \times 10^{-8}$. PCa GWAS data MNLP conditioning neutralizes the rs530534670, rs199577062 effects, demonstrating the MNLP genotypes power. Summary-based conditional analysis was performed to condition all SNPs in the GWAS locus on the predicted complex variant (MNLP) and quantify the residual association. Colocalization analysis was performed using COLOC test to evaluate the probability that MNLP and the GWAS share the same causal variant, using all SNP-MNLP associations in the locus as inputs.

a

| | IID | PAT | MAT | SEX | PHENOTYPE | rs530543670_G | rs199577062_T | INDEL |
|---|---|---|---|---|---|---|---|---|
| ** | NA12718 | 0 | 0 | 2 | -9 | 1 | 1 | 1 |
| | NA12748 | 0 | 0 | 1 | -9 | 2 | 1 | 1 |
| | NA12775 | 0 | 0 | 1 | -9 | 1 | 1 | 1 |
| *** | NA12777 | 0 | 0 | 1 | -9 | 0 | 0 | 0 |
| * | NA12778 | 0 | 0 | 2 | -9 | 2 | 2 | 2 |
| | NA12827 | 0 | 0 | 1 | -9 | 2 | 1 | 1 |
| | NA12829 | 0 | 0 | 1 | -9 | 1 | 0 | 0 |
| | NA12842 | 0 | 0 | 1 | -9 | 1 | 1 | 1 |
| | NA12843 | 0 | 0 | 2 | -9 | 2 | 2 | 2 |
| | NA12889 | 0 | 0 | 1 | -9 | 2 | 1 | 1 |
| | NA12890 | 0 | 0 | 2 | -9 | 1 | 1 | 1 |
| | NA18488 | 0 | 0 | 2 | -9 | 1 | 1 | 1 |
| | NA18530 | 0 | 0 | 1 | -9 | 2 | 1 | 1 |
| | NA18534 | 0 | 0 | 1 | -9 | 2 | 2 | 2 |
| | NA18536 | 0 | 0 | 1 | -9 | 2 | 2 | 2 |
| | NA18543 | 0 | 0 | 1 | -9 | 2 | 2 | 2 |
| | NA18544 | 0 | 0 | 1 | -9 | 2 | 1 | 1 |
| | NA18546 | 0 | 0 | 1 | -9 | 2 | 2 | 2 |
| | [illegible] | [illegible] | [illegible] | [illegible] | [illegible] | [illegible] | [illegible] | [illegible] |
| | [illegible] | | | | | | | |

b

**rs199577062**

21 bp short (S) allele (rs745614767)

GG

tccgt-------------------CAGG-ACTT-G----CAAGCT---CATGTGgcgtc

tccgtGCCGCACTGTTGATCTTGGCAGGCCCTTCGGGACCAACAGGGACA-GT-gcgtc

47 bp long (L) allele (rs386684493)

G

c

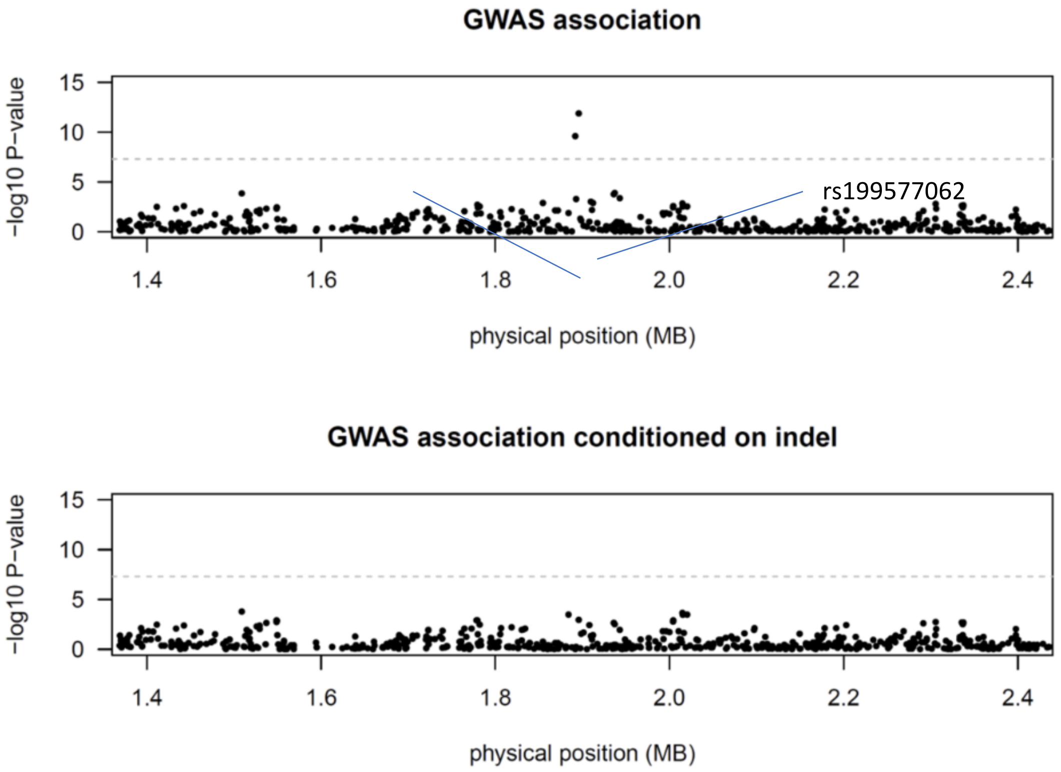

**Supplementary Method Figure SMF8.**

**a.** Coverage analysis of two random group of samples at the IRX4 ~1kb genomic region. Coverage fluctuates around the 0 expected value. MNLP position has significantly different coverage due to the alignment of the S allele to the L allele containing reference genome.

**b.** Genotype driven coverage data at the MNLP position. Reference “C” allele samples (blue line), which carrying the L allele has larger coverage at the MNLP position due the perfect alignment of the L allele carrying reference genome. Recombinant samples (C/S allele) cause the deviation from the expected 0 value. T allele containing samples at the rs12653946 position (red line) associated with the S allele has significantly lower coverage at the INDEL position aligned against the L allele containing reference genome.

**c.** Genotype driven coverage-based difference at the INDEL position. Comparing random (blue line) and systematically genotyped (rs12653946 “C” (ref) and “T”(mut)) (red line) groups coverage ratios revealed rs12653946 correlated deletion.

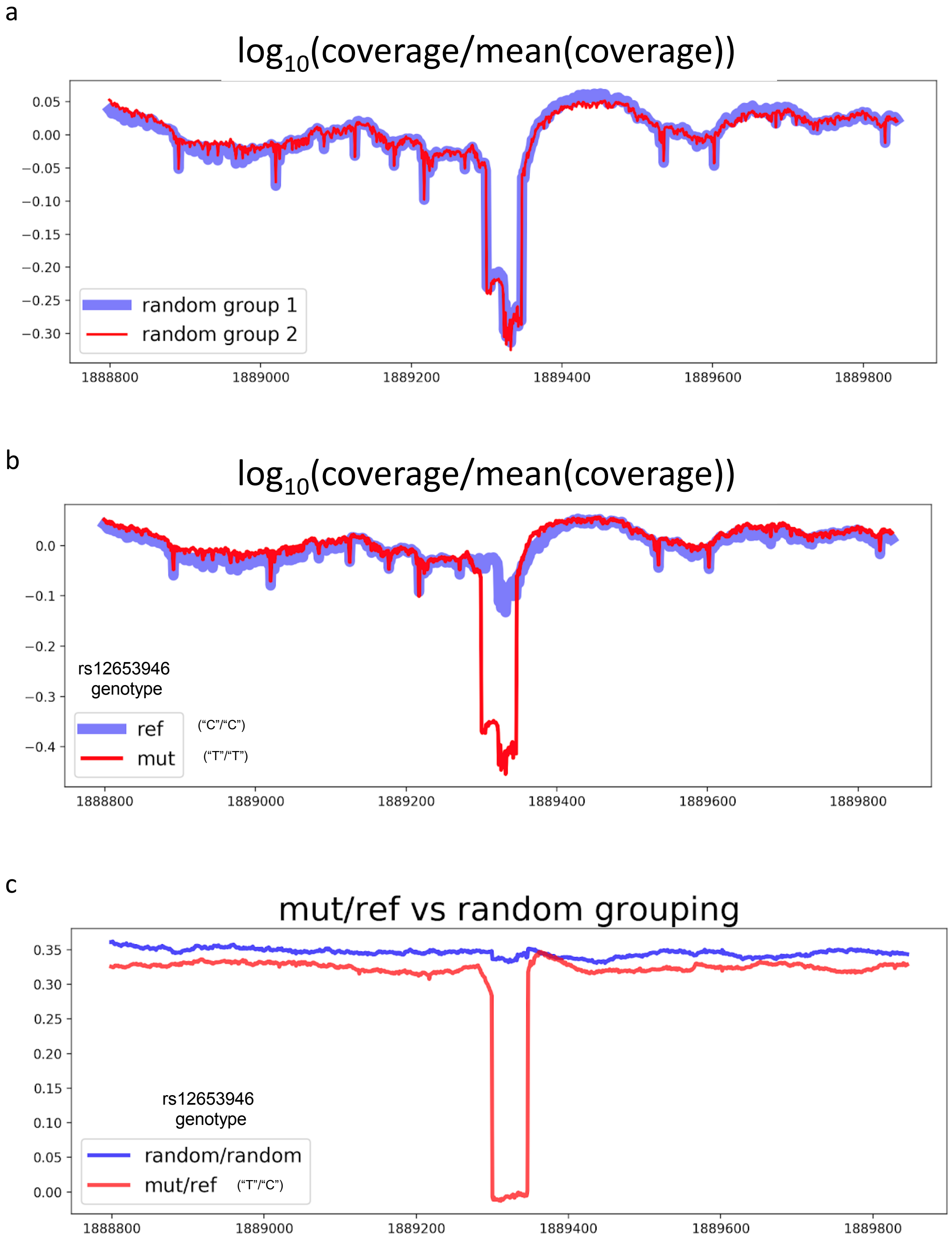
a
log10(coverage/mean(coverage))
0.05
0.00
−0.05
−0.10
−0.15
−0.20
−0.25
−0.30
random group 1
random group 2
1888800
1889000
1889200
1889400
1889600
1889800
b
log10(coverage/mean(coverage))
0.0
−0.1
−0.2
−0.3
−0.4
rs12653946
genotype
ref ("C"/"C")
mut ("T"/"T")
1888800
1889000
1889200
1889400
1889600
1889800
c
mut/ref vs random grouping
0.35
0.30
0.25
0.20
0.15
0.10
0.05
0.00
rs12653946
genotype
random/random
mut/ref ("T"/"C")
1888800
1889000
1889200
1889400
1889600
1889800

**Supplementary Method Figure SMF9.**

A representative example to determine haploblock size to download BAM slices. Correlation coefficient 0.2 (blue line) were applied to determine haploblock start and stop positions (red lines).

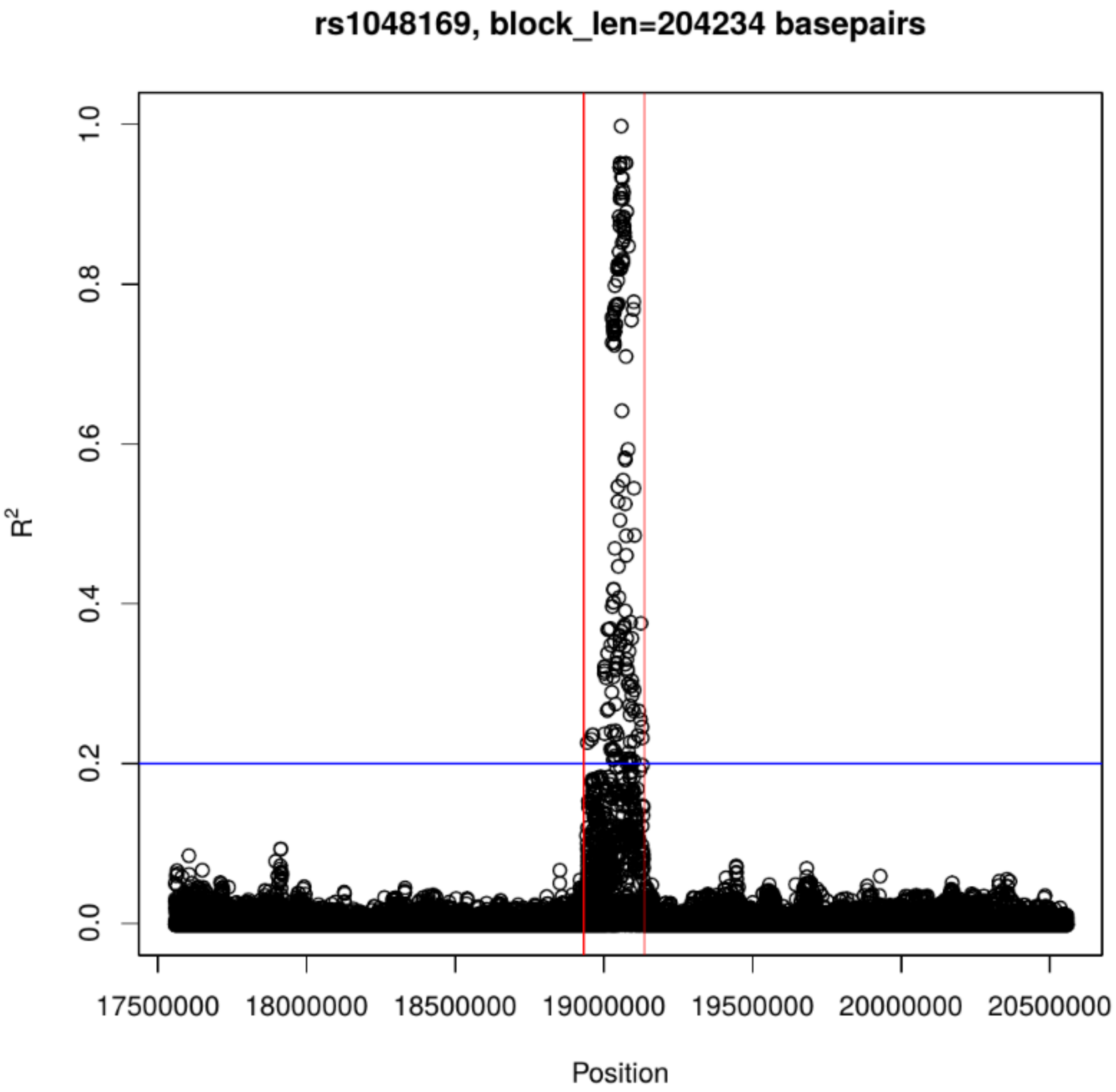
rs1048169, block_len=204234 basepairs
R^2
1.0
0.8
0.6
0.4
0.2
0.0
17500000
18000000
18500000
19000000
19500000
20000000
20500000
Position

**Supplementary Method Figure SMF10.**

Haploblock sizes and positions of the 147 PCa risk regions centered with the leading SNP position.

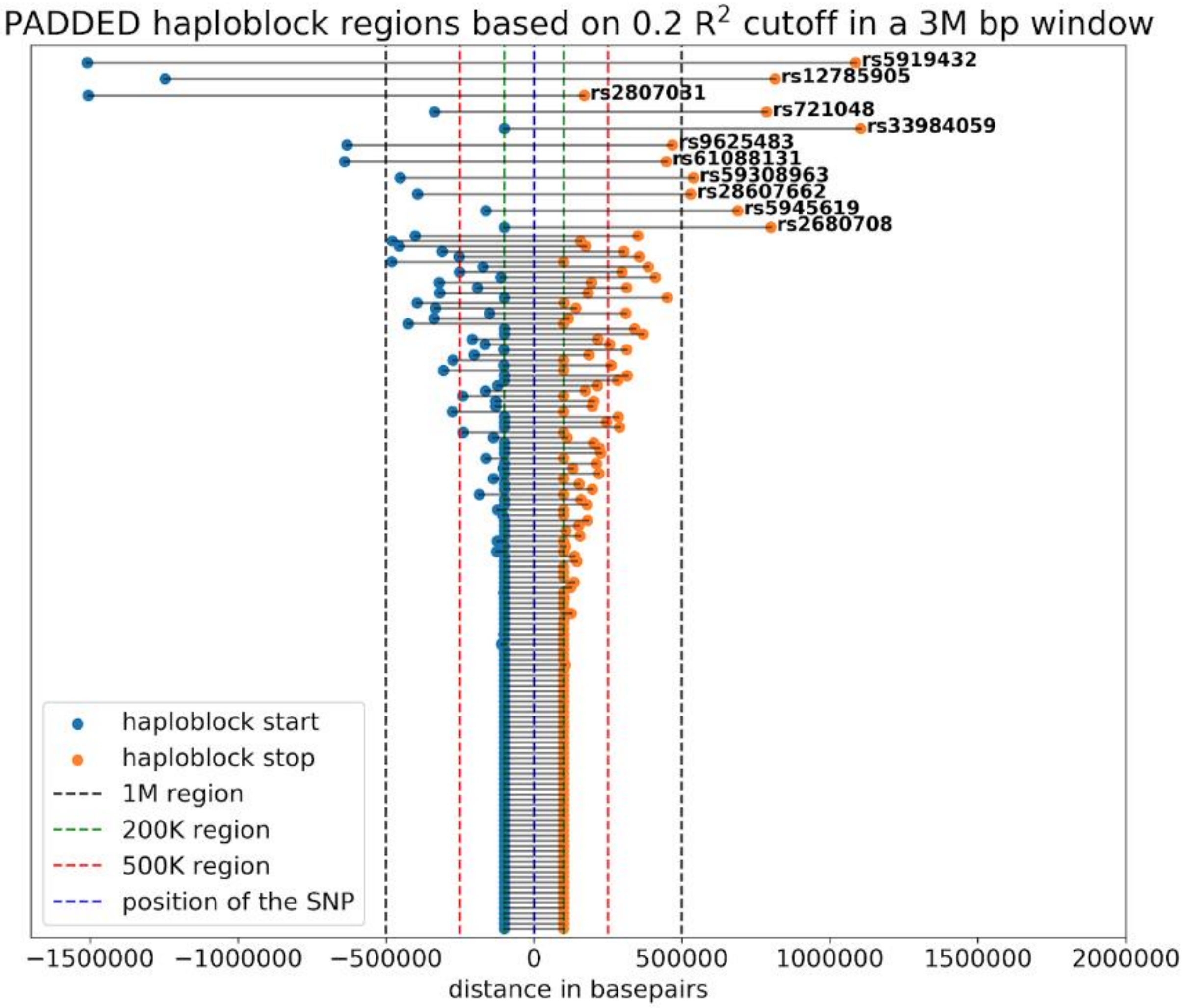

PADDED haploblock regions based on 0.2 R2 cutoff in a 3M bp window
rs5919432
rs12785905
rs2807031
rs721048
rs33984059
rs9625483
rs61088131
rs59308963
rs28607662
rs5945619
rs2680708
haploblock start
haploblock stop
1M region
200K region
500K region
position of the SNP
−1500000
−1000000
−500000
0
500000
1000000
1500000
2000000
distance in basepairs
