# Supplementary material for: A biallelic multiple nucleotide length polymorphism explains functional causality at 5p15.33 prostate cancer risk locus
Source: Nat Commun. 2023 Aug 23;14:5118. doi: 10.1038/s41467-023-40616-z (PMC10447552; doi:10.1038/s41467-023-40616-z)
Supplement: Supplementary file 2 — Description of Additional Supplementary Files [file 41467_2023_40616_MOESM2_ESM.pdf]

Description of Additional Supplementary Files

File Name: Supplementary Data 1
Description: S and L allele specific reference sequences.

File Name: Supplementary Data 2a
Description: Differentially expressed genes (CTRL vs. OE).

File Name: Supplementary Data 2b
Description: Differentially expressed genes (CTRL vs. KD).

File Name: Supplementary Data 2c
Description: Differentially expressed genes (KD vs. OE).

File Name: Supplementary Data 2d
Description: Sequentially altered genes along KD>CTRL>OE or KD<CTRL<OE

File Name: Supplementary Data 2e
Description: Sequentially altered genes along KD>CTRL>OE or KD<CTRL<OE (For both C vs KD and OE vs C $p<0.05$).

File Name: Supplementary Data 3
Description: Identified (135) correlated complex variants.

File Name: Supplementary Data 4
Description: Validated (16) correlated complex variants.

File Name: Supplementary Data 5
Description: Publicly available data sets used in this study.

File Name: Supplementary Data 6
Description: PCR primer, gRNA and shRNA sequences used in this study.
