# Supplementary material for: A biallelic multiple nucleotide length polymorphism explains functional causality at 5p15.33 prostate cancer risk locus
Source: Nat Commun. 2023 Aug 23;14:5118. doi: 10.1038/s41467-023-40616-z (PMC10447552; doi:10.1038/s41467-023-40616-z)
Supplement: Supplementary file 4 — Reporting Summary [file 41467_2023_40616_MOESM4_ESM.pdf]

nature portfolio

Corresponding author(s): Matthew L. Freedman

Last updated by author(s): May 5, 2023

Reporting Summary

Nature Portfolio wishes to improve the reproducibility of the work that we publish. This form provides structure for consistency and transparency in reporting. For further information on Nature Portfolio policies, see our Editorial Policies and the Editorial Policy Checklist.

Statistics

For all statistical analyses, confirm that the following items are present in the figure legend, table legend, main text, or Methods section.

n/a	Confirmed	
☐	☒	The exact sample size (*n*) for each experimental group/condition, given as a discrete number and unit of measurement
☐	☒	A statement on whether measurements were taken from distinct samples or whether the same sample was measured repeatedly
☐	☒	The statistical test(s) used AND whether they are one- or two-sided<br>*Only common tests should be described solely by name; describe more complex techniques in the Methods section.*
☐	☒	A description of all covariates tested
☐	☒	A description of any assumptions or corrections, such as tests of normality and adjustment for multiple comparisons
☐	☒	A full description of the statistical parameters including central tendency (e.g. means) or other basic estimates (e.g. regression coefficient) AND variation (e.g. standard deviation) or associated estimates of uncertainty (e.g. confidence intervals)
☐	☒	For null hypothesis testing, the test statistic (e.g. *F*, *t*, *r*) with confidence intervals, effect sizes, degrees of freedom and *P* value noted<br>*Give P values as exact values whenever suitable.*
☒	☐	For Bayesian analysis, information on the choice of priors and Markov chain Monte Carlo settings
☒	☐	For hierarchical and complex designs, identification of the appropriate level for tests and full reporting of outcomes
☒	☐	Estimates of effect sizes (e.g. Cohen's *d*, Pearson's *r*), indicating how they were calculated

Our web collection on statistics for biologists contains articles on many of the points above.

Software and code

Policy information about availability of computer code

Data collection	Data collection and analyses were performed R version 4.0.1. Raw Illumina output was converted to fastq format using Illumina bcl2fastq v2.18
Data analysis	BWA 0.7.17 (http://bio-bwa.sourceforge.net/) for mapping;<br>MACS2 2.1.2 (http://liulab.dfci.harvard.edu/MACS/) for peak calling;<br>DEseq2 1.28.0 (https://bioconductor.org/packages/release/bioc/html/DESeq2.html) for differen?al analysis;<br>ChiLin2.0 (http://cistrome.org/chilin/) for QC<br>SnapGene Viewer 5.0.2.

For manuscripts utilizing custom algorithms or software that are central to the research but not yet described in published literature, software must be made available to editors and reviewers. We strongly encourage code deposition in a community repository (e.g. GitHub). See the Nature Portfolio guidelines for submitting code & software for further information.

Data

Policy information about availability of data

All manuscripts must include a data availability statement. This statement should provide the following information, where applicable:

- Accession codes, unique identifiers, or web links for publicly available datasets
- A description of any restrictions on data availability
- For clinical datasets or third party data, please ensure that the statement adheres to our policy

Data sets generated in this study have been deposited in the Gene Expression Omnibus (GEO) database under accession code GSE231751 of super series including RNA-seq (GSE231747), ChIP-seq (GSE231747) and ATAC-seq (GSE231750) data. Sequencing reads are aligned to the human genome build hg19. Further information and requests for resources and reagents should be directed to and will be fulfilled by the lead contact, Matthew Freedman (matthew_freedman@dfci.harvard.edu). Sequencing reads are aligned to the human genome build hg19. The datasets generated in this study have been deposited in the Gene Expression Omnibus (GEO) database under accession code (GSE231751; https://www.ncbi.nlm.nih.gov/geo/query/acc.cgi?acc=GSE231751).
The following data/data sets used in this study:
GSM2186479
GSM4648970
GSM4039424
GSM1328982
GSE70079
GSE130408
GSM1328945
GSM1527834
GSM1249447
GSM1145322
GSM4037036
GSM4037037
GSM4037038
GSM4037039
GSM4037040
GSM4037041
GSM1308235
GSM1308237
ftp://ftp.sra.ebi.ac.uk/vol1/run/ERR323/ERR3239480/NA12718.final.cram
ftp://ftp.sra.ebi.ac.uk/vol1/run/ERR323/ERR3239481/NA12748.final.cram
ftp://ftp.sra.ebi.ac.uk/vol1/run/ERR323/ERR3239482/NA12775.final.cram
ftp://ftp.sra.ebi.ac.uk/vol1/run/ERR323/ERR3239483/NA12777.final.cram
ftp://ftp.sra.ebi.ac.uk/vol1/run/ERR323/ERR3239484/NA12778.final.cram
ftp://ftp.sra.ebi.ac.uk/vol1/run/ERR323/ERR3239485/NA12827.final.cram
ftp://ftp.sra.ebi.ac.uk/vol1/run/ERR323/ERR3239486/NA12829.final.cram
ftp://ftp.sra.ebi.ac.uk/vol1/run/ERR323/ERR3239487/NA12842.final.cram
ftp://ftp.sra.ebi.ac.uk/vol1/run/ERR323/ERR3239488/NA12843.final.cram
ftp://ftp.sra.ebi.ac.uk/vol1/run/ERR323/ERR3239489/NA12889.final.cram
ftp://ftp.sra.ebi.ac.uk/vol1/run/ERR323/ERR3239490/NA12890.final.cram
ftp://ftp.sra.ebi.ac.uk/vol1/run/ERR323/ERR3239491/NA18488.final.cram
ftp://ftp.sra.ebi.ac.uk/vol1/run/ERR323/ERR3239492/NA18530.final.cram
ftp://ftp.sra.ebi.ac.uk/vol1/run/ERR323/ERR3239493/NA18534.final.cram
ftp://ftp.sra.ebi.ac.uk/vol1/run/ERR323/ERR3239494/NA18536.final.cram
ftp://ftp.sra.ebi.ac.uk/vol1/run/ERR323/ERR3239495/NA18543.final.cram
ftp://ftp.sra.ebi.ac.uk/vol1/run/ERR323/ERR3239496/NA18544.final.cram
ftp://ftp.sra.ebi.ac.uk/vol1/run/ERR323/ERR3239497/NA18546.final.cram
ftp://ftp.sra.ebi.ac.uk/vol1/run/ERR323/ERR3239498/NA18548.final.cram
ftp://ftp.sra.ebi.ac.uk/vol1/run/ERR323/ERR3239499/NA18549.final.cram

Research involving human participants, their data, or biological material

Policy information about studies with human participants or human data. See also policy information about sex, gender (identity/presentation), and sexual orientation and race, ethnicity and racism.

Reporting on sex and gender	Radical prostatecotmy specimens from adult (≥ 18 years) males were selected from the Dana-Farber Cancer Institute (DFCI) Gelb Center biobank and database. All subjects were enrolled on DFCI Protocol 01-045, approved by the Dana-Farber Cancer Institute/Harvard Cancer Center IRB.
Reporting on race, ethnicity, or other socially relevant groupings	Patients were not recruited or excluded based on age, ethnicity or clinical state of disease.
Population characteristics	This study involved the selection of prostatectomy specimens obtained from adult males aged over 18 years for ChIP-seq analyses.
Recruitment	Cohorts in the biobanking protocols described above were comprised of consecutive series of men undergoing radical

Recruitment	prostatectomy at DFCI.
Ethics oversight	Dana-Farber Cancer Institute/Harvard Cancer Center IRB.

Note that full information on the approval of the study protocol must also be provided in the manuscript.

Field-specific reporting

Please select the one below that is the best fit for your research. If you are not sure, read the appropriate sections before making your selection.

☒ Life sciences ☐ Behavioural & social sciences ☐ Ecological, evolutionary & environmental sciences

For a reference copy of the document with all sections, see nature.com/documents/nr-reporting-summary-flat.pdf

Life sciences study design

All studies must disclose on these points even when the disclosure is negative.

Sample size	Sample size was determined based on previous analyses (Pomerantz et al., Nature Genetics, 2015; Stelloo et al., EMBO Mol Med, 205) demonstrating ample sample sizes for identifying cistromic changes in human tissue.
Data exclusions	No data were excluded from the analysis.
Replication	Experiments were performed using three biological replicates (n=3). All attempts at replication were successful and no data was excluded.
Randomization	N/A - This study not includes human research.
Blinding	N/A - The study involved direct interaction or observation, where blinding was not feasible or practical to implement.

Reporting for specific materials, systems and methods

We require information from authors about some types of materials, experimental systems and methods used in many studies. Here, indicate whether each material, system or method listed is relevant to your study. If you are not sure if a list item applies to your research, read the appropriate section before selecting a response.

Materials & experimental systems

n/a	Involved in the study	
☐	☒	Antibodies
☐	☒	Eukaryotic cell lines
☒	☐	Palaeontology and archaeology
☒	☐	Animals and other organisms
☒	☐	Clinical data
☒	☐	Dual use research of concern
☒	☐	Plants

Methods

n/a	Involved in the study	
☐	☒	ChIP-seq
☒	☐	Flow cytometry
☒	☐	MRI-based neuroimaging

Antibodies

Antibodies used	Sonicated chromatin was incubated overnight with 6 µg of antibody—H3K27ac (Diagenode Cat# C15410196) and bound to protein A and protein G beads (Life Technologies).
Validation	H3K27Ac: reference - PMID 30773341; PMID: 32690948; PMID: 36071171; product datasheet - https://www.diagenode.com/en/documents/datasheeth3k27ac-<br>C15410196

Eukaryotic cell lines

Policy information about cell lines and Sex and Gender in Research

Cell line source(s)	All prostate canacer cell lines (LNCaP, VCaP, 22Rv1 and PC-3) were obtained from ATCC.
Authentication	No further authentication was performed.
Mycoplasma contamination	Cell lines tested negative for mycoplasma contamination in every 3 weeks.

Commonly misidentified lines (See ICLAC register)	No cell lines used in this study are listed in the database of commonly misidentified cell lines maintained by ICLAC.

Plants

Seed stocks	*Report on the source of all seed stocks or other plant material used. If applicable, state the seed stock centre and catalogue number. If plant specimens were collected from the field, describe the collection location, date and sampling procedures.*
Novel plant genotypes	*Describe the methods by which all novel plant genotypes were produced. This includes those generated by transgenic approaches, gene editing, chemical/radiation-based mutagenesis and hybridization. For transgenic lines, describe the transformation method, the number of independent lines analyzed and the generation upon which experiments were performed. For gene-edited lines, describe the editor used, the endogenous sequence targeted for editing, the targeting guide RNA sequence (if applicable) and how the editor was applied.*
Authentication	*Describe any authentication procedures for each seed stock used or novel genotype generated. Describe any experiments used to assess the effect of a mutation and, where applicable, how potential secondary effects (e.g. second site T-DNA insertions, mosiacism, off-target gene editing) were examined.*

ChIP-seq

Data deposition

☒ Confirm that both raw and final processed data have been deposited in a public database such as GEO.

☒ Confirm that you have deposited or provided access to graph files (e.g. BED files) for the called peaks.

Data access links *May remain private before publication.*	https://www.ncbi.nlm.nih.gov/geo/query/acc.cgi?acc=GSE231751
Files in database submission	GSM7300027 LNCaP cells, vector control rep1<br>GSM7300028 LNCaP cells, vector control rep2<br>GSM7300029 LNCaP cells, IRX4 knock down rep1<br>GSM7300030 LNCaP cells, IRX4 knock down rep2<br>GSM7300031 LNCaP cells, IRX4 overexpression rep1<br>GSM7300032 LNCaP cells, IRX4 overexpression rep2<br>GSM7300033 LNCaP_parental, H3K27ac<br>GSM7300034 LNCaP_clone1, H3K27ac<br>GSM7300035 LNCaP_clone2, H3K27ac<br>GSM7300038 LNCaP_parental, ATAC<br>GSM7300039 LNCaP_clone1, ATAC<br>GSM7300040 LNCaP_clone2, ATAC
Genome browser session (e.g. UCSC)	N/A

Methodology

Replicates	Biological replicates (isogenic clones) were used to confirm IRX4 enhancer epigenetic activity gain.
Sequencing depth	Each sample were sequenced at least by 30M PE150 reads.
Antibodies	H3K27ac (Diagenode, Cat# C15410196)
Peak calling parameters	All samples were processed through the computational pipeline developed at the DFCI Center for Functional Cancer Epigenetics (CFCE) using primarily open source programs. Sequence tags were aligned with Burrows-Wheeler Aligner (BWA) to build hg19 of the human genome, and uniquely mapped, non-redundant reads were retained. These reads were used to generate binding sites with Model-Based Analysis of ChIP-seq 2 (MACS v2.1.1.20160309), with a q-value (FDR) threshold of 0.01.
Data quality	We evaluated multiple quality control criteria based on alignment information and peak quality: (i) sequence quality score; (ii) uniquely mappable reads (reads that can only map to one location in the genome); (iii) uniquely mappable locations (locations that can only be mapped by at least one read); (iv) peak overlap with Velcro regions, a comprehensive set of locations—also called consensus signal artifact regions—in the human genome that have anomalous, unstructured high signal or read counts in next generation sequencing experiments independent of cell line and of type of experiment; (v) number of total peaks (the minimum required was 1,000); (vi) high-confidence peaks (the number of peaks that are tenfold enriched over background); (vii) percentage overlap with known DHS sites derived from the ENCODE Project (the minimum required to meet the threshold was 80%); and (viii) peak conservation (a measure of sequence similarity across species based on the hypothesis that conserved sequences are more likely to be functional). Typically, if a sample fails one of these criteria, it will fail many (locations with low mappability will likely have low peak numbers, many of which will likely be in high-mappability regions, etc.).
Software	MACS v2.1.1.20140616 was used for ChIP-seq peak calling.
